# Imaging nanoscopic co-assemblies of α-synuclein and Tau in human samples

**DOI:** 10.1038/s41467-026-77728-1

**Published:** 2026-09-18

**Authors:** Yu P. Zhang, Amber Wallis, Clara Obensa Bertroli, Cherida Zhang, Melanie Burke, Florence Layburn, Evgeniia Lobanova, Martin Furlepa, Shekhar Kedia, Yuhao Cui, Prasanna Suresh, Woo Suk Yang, Annelies Quaegebeur, Tim Bartels, Caroline H. Williams-Gray, David Klenerman

**Affiliations:** 1 Department of Chemistry, University of Cambridge, Cambridge, UK; 2 UK Dementia Research Institute at Cambridge, Cambridge, UK; 3 UK Dementia Research Institute at University College London, London, UK; 4 Department of Clinical Neurosciences, University of Cambridge, Cambridge, UK; 5 Manchester Centre for Clinical Neurosciences, Salford Royal NHS Foundation Trust, Manchester Academic Health Science Centre, Manchester, UK; 6 St. Thomas’ Hospital London, London, UK

**Keywords:** Molecular neuroscience, Neurodegeneration, Single-molecule biophysics, Protein aggregation

## Abstract

α-synuclein (αS), amyloid-β (Aβ), and Tau aggregates are hallmarks of many neurodegenerative diseases, yet the prevalence and nature of their nanoscopic co-assemblies remain poorly understood. Here, we adapt a single-molecule pull-down (SiMPull) assay to profile αS, Aβ, and Tau co-assemblies across post-mortem brain tissue homogenates, soluble brain fractions, human serum, cerebrospinal fluid, and saliva. We show that αS–Tau co-assemblies are abundant across all sample matrices, accounting for ~10–70% of total αS aggregates in both Parkinson’s disease (PD) and control samples. Notably, αS–Tau co-assemblies in soluble brain fractions from controls exhibit higher Proteinase K (PK) resistance than those from PD cases. Biophysically, Tau accelerates αS aggregation in simple in vitro systems, but this effect is suppressed in complex environments. Together, this work establishes a framework for probing co-assemblies, revealing that αS–Tau species possess heterogeneous biophysical features and are unlikely to act as a single, uniformly toxic species in PD pathology.

## Abstract (web summary)

Here the authors employ an optimised single-molecule pull-down (SiMPull) to investigate nanoscopic αS, Tau, and Aβ-containing assemblies in human brain tissue and biofluids. They establish a framework for detecting and comparing nanoscopic co-assemblies.

## Introduction

The abnormal aggregation of α-synuclein (αS) in the human brain is a hallmark of Parkinson’s disease (PD)^1–3^. However, αS aggregates are not the only aggregates detected in PD. Tau aggregates are present in nearly 50% of brains with Lewy body pathology, while Aβ deposition is often associated with cognitive decline in PD^4–8^. Notably, Lewy bodies, the pathological hallmark of PD, have been found to contain Tau in addition to αS^6,9^. Several model systems demonstrate that the co-existence of these proteins can modulate their toxicity. For example, co-expression of Tau and αS in yeast enhances αS toxicity^10^; neuronal co-expression of human Aβ and αS in *Caenorhabditis elegans* induces behavioural deficits that mimic features of Lewy body dementia^11^. More recently, Xiang et al. reported that αS and Tau co-pathology can spread into the brain in gut-inducible SYN103^+/−^ and/or TAU368^+/−^ transgenic mouse, eliciting behavioural defects^12^. These findings suggest that the coexistence of αS, Aβ, and Tau may modify their functions and contribute to the altered pathophysiological outcomes in neurodegeneration.

Direct molecular interactions may underlie this effect. Evidence from in vitro and in vivo models indicates that αS can co-aggregate with both Aβ and Tau, producing co-assemblies with altered toxicity^11,13–19^. Although such co-assemblies have been found in human brains, relevant studies often focused on large inclusions (1–10 μm), which are characteristic of more advanced stages of protein aggregation^6,20,21^. In contrast, smaller soluble species (50–400 nm), which form earlier during aggregation and are thought to be more toxic, remain poorly characterised with respect to their potential to form co-assemblies^2,22,23^.

Studying nanoscopic co-assemblies in human samples may provide insights into early changes in neurodegeneration. The technical barrier is largely due to their nanometre-scale size and low abundance^24^. Conventional detection methods often lack the sensitivity and resolution required, limiting our understanding of their contribution to disease progression. To overcome these limitations, we employ a single-molecule pull-down (SiMPull) assay for relevant characterisation^25–27^. This assay captures and visualises target molecules on a chemically modified surface designed to suppress non-specific binding. (Fig. 2A) It has since been adapted to characterise protein aggregates in human samples, in addition to its original application in studying protein–protein interactions in cellular models^22,25,26,28–32^. We have optimised the surface chemistry to further reduce background signals, achieving improved performance compared with conventional PEG-based SiMPull coatings^33^.

Here, we apply an optimised SiMPull platform, with reduced background signal compared with conventional PEG-based coatings, to investigate nanoscopic αS, Tau, and Aβ-containing assemblies in human brain tissue and biofluids. αS–Tau co-assemblies can be specifically detected across all sample types, whereas other co-assemblies are absent or fall below detection thresholds in certain samples, especially the brain. By integrating complementary antibody-pairing strategies, immunodepletion and proteinase K (PK) resistance assays, we characterise the abundance, structural properties, and biochemical stability of these disease-associated nanoscopic species. These co-assemblies represent a distinct population of protein complexes associated with αS and Tau, with features that vary depending on biological context (e.g. brain homogenate vs soluble fractions) and disease state. By combining these single-molecule imaging-based methods, we establish a framework for detecting and comparing nanoscopic co-assemblies across diverse human samples, providing insights into their role in neurodegeneration.

## Results

### Recombinant αS and Aβ aggregates form stable nanoscopic co-assemblies with Tau aggregates in vitro

**Fig. 1:**
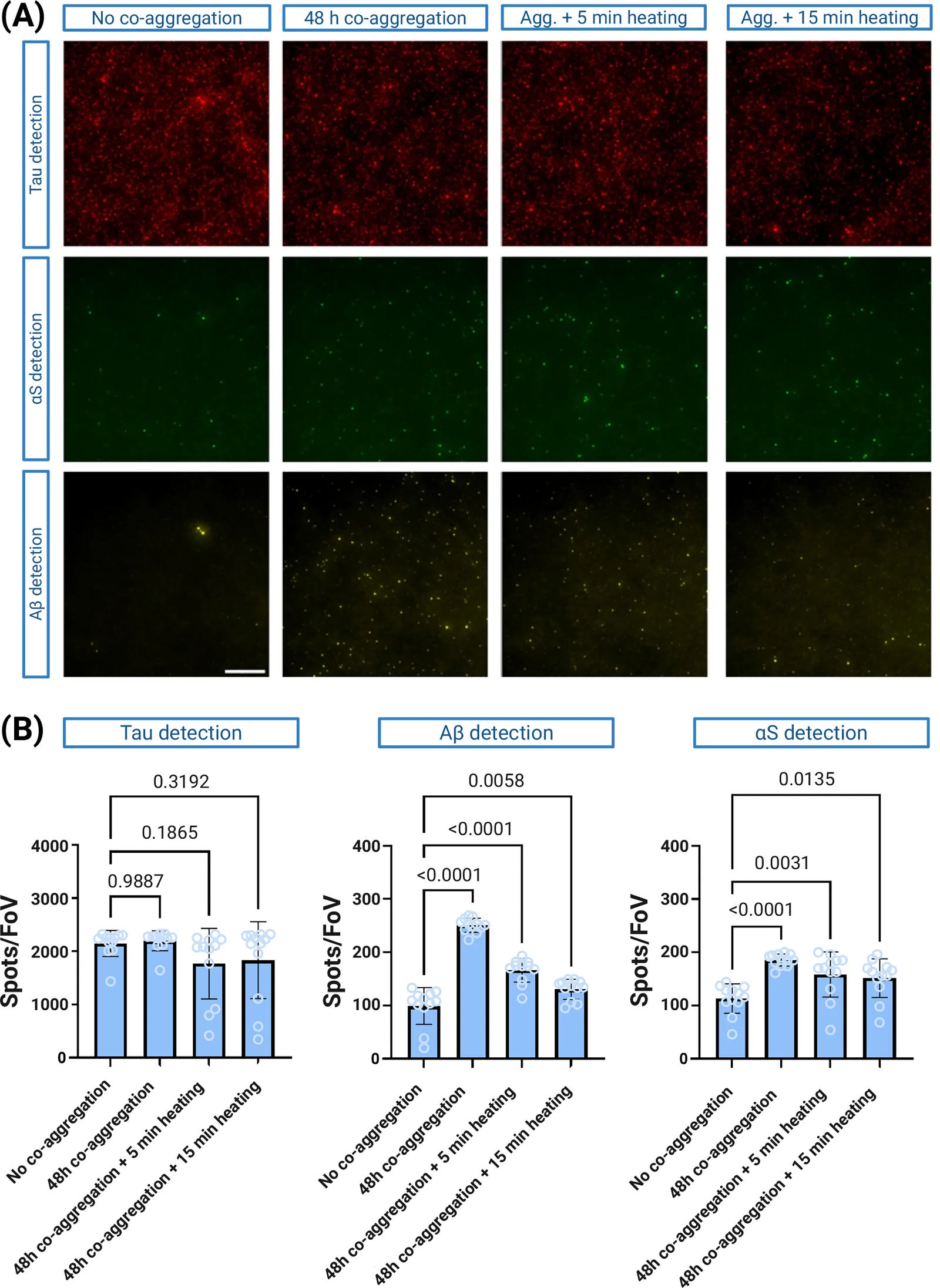
Multicolour single-molecule imaging of Tau–αS–Aβ co-assemblies. Mixed Tau, αS, and Aβ aggregates generated under different co-aggregation conditions or treatments were applied to a SiMPull surface coated with Tau antibodies and imaged with detection antibodies against Tau, αS, and Aβ. **A** Representative images. Tau (AF647–HT7 antibody) is shown in red, Aβ (AF488–6E10 antibody) in green, and αS (AF546–Syn211 antibody) in yellow. Because the surface selectively captures Tau-containing species, little αS or Aβ signal was detected without co-aggregation. Co-aggregation markedly increased the αS and Aβ signals, indicating the formation of stable Tau-associated co-assemblies. Scale bar, 10 µm. **B** Quantification of single-molecule counts for Tau-, αS-, and Aβ-positive species under different conditions. Ordinary One-way ANOVA with post-hoc multiple-comparison correction was used for statistical test. Data represent mean ± s.d.; *n* = 12. From 12 fields of view (FoV). All panels were assembled as Fig. 1 in BioRender. Zhang, Y. (2026) https://BioRender.com/9lbi36n. Source data are provided as a Source Data file.

It has been widely reported that monomeric αS, Aβ, and Tau can form co-assemblies in vitro^11,13–19^. Their direct interaction suggests that pathological protein aggregates may not exist solely as distinct homotypic species but can also form heterotypic assemblies with potentially distinct roles in neurodegeneration^34^. Here, we further demonstrate that such co-assemblies can also arise between pre-formed aggregates at nanometre-scale. Using three-colour SiMPull imaging, we analysed recombinant αS, Aβ, and Tau under different co-aggregation conditions, with each fluorescence channel corresponding to one of the three proteins. (Fig. 1) The imaging surface was functionalised with Tau antibodies and selectively captured Tau-containing species rather than homotypic αS or Aβ aggregates. Control experiments further confirmed that αS and Aβ aggregates do not bind non-specifically to this Tau antibody (Supplementary Fig. 10). The fluorescent readout therefore reflects how αS and Aβ associate with Tau-containing molecules. The following antibodies were used for the measurements: Syn211 for αS, 6E10 for Aβ, and HT7 for Tau. We define aggregates as protein complexes composed of at least two repeating subunits, which represents the minimal structure capable of generating a measurable signal when identical epitope-specific antibodies are applied for both capture and detection^28^.

As shown in Fig. 1, oligomeric Tau formed stable co-assemblies with αS and Aβ after 48-hour incubation. Upon co-aggregation, the αS and Aβ signals increased markedly, confirming the formation of co-assemblies. The resulting assemblies remain nanoscopic rather than forming mature fibrils under our experimental conditions, suggesting that protein co-assemblies can stably exist within the intermediate size range. In contrast, aggregates that were simply mixed without co-aggregation showed minimal αS or Aβ signal, indicating that passive mixing is insufficient to generate stable co-assemblies. The colocalised signal also supports this observation: little colocalisation was detected upon passive mixing, whereas co-aggregation markedly increased the fraction of colocalised αS, Aβ, and Tau signals. (Supplementary Fig. 1) AmyTracker imaging revealed low fluorescence signal from these species, suggesting that the formation of these stable co-assemblies does not necessarily require extensive β-sheet architecture. (Supplementary Fig. 2) Notably, when the samples were heated to 95 °C, most co-assemblies persisted, demonstrating their structural stability. Approximately 80% of Tau aggregates, 80% of Tau-αS co-aggregates, and 50% of Tau-Aβ co-aggregates remain detectable after 15 minutes of heating. Together, these results indicate that stable αS, Aβ and Tau co-assemblies can exist as stable, non-fibrillar, nanoscopic species.

### Limitations of antibody-based multi-colour imaging on nanoscopic protein assemblies

While multi-colour imaging is widely used to assess the composition of molecular assemblies^6,8,9,13^, small protein assemblies could be challenging for antibody-based multi-colour imaging. In contrast to large inclusions, the nanoscale dimensions of these smaller assemblies severely restrict epitope accessibility. Steric hindrance precludes the simultaneous binding of multiple antibodies, even when multiple target epitopes are physically present. Consequently, certain binding events are obstructed, thereby limiting the reliable detection of co-localised signals. To confirm this, we compared single-molecule imaging of αS–Aβ co-assemblies under single- and dual-antibody imaging conditions. Only αS detection antibody was used under the single-antibody condition; whereas both αS and Aβ detection antibodies were applied under the dual-antibody condition. By quantifying αS signal intensities under both experimental conditions, we evaluated whether the presence of an additional antibody alters the readout within the primary αS detection channel. These experiments were performed using recombinant αS–Aβ co-assemblies and pooled human serum.

**Fig. 2:**
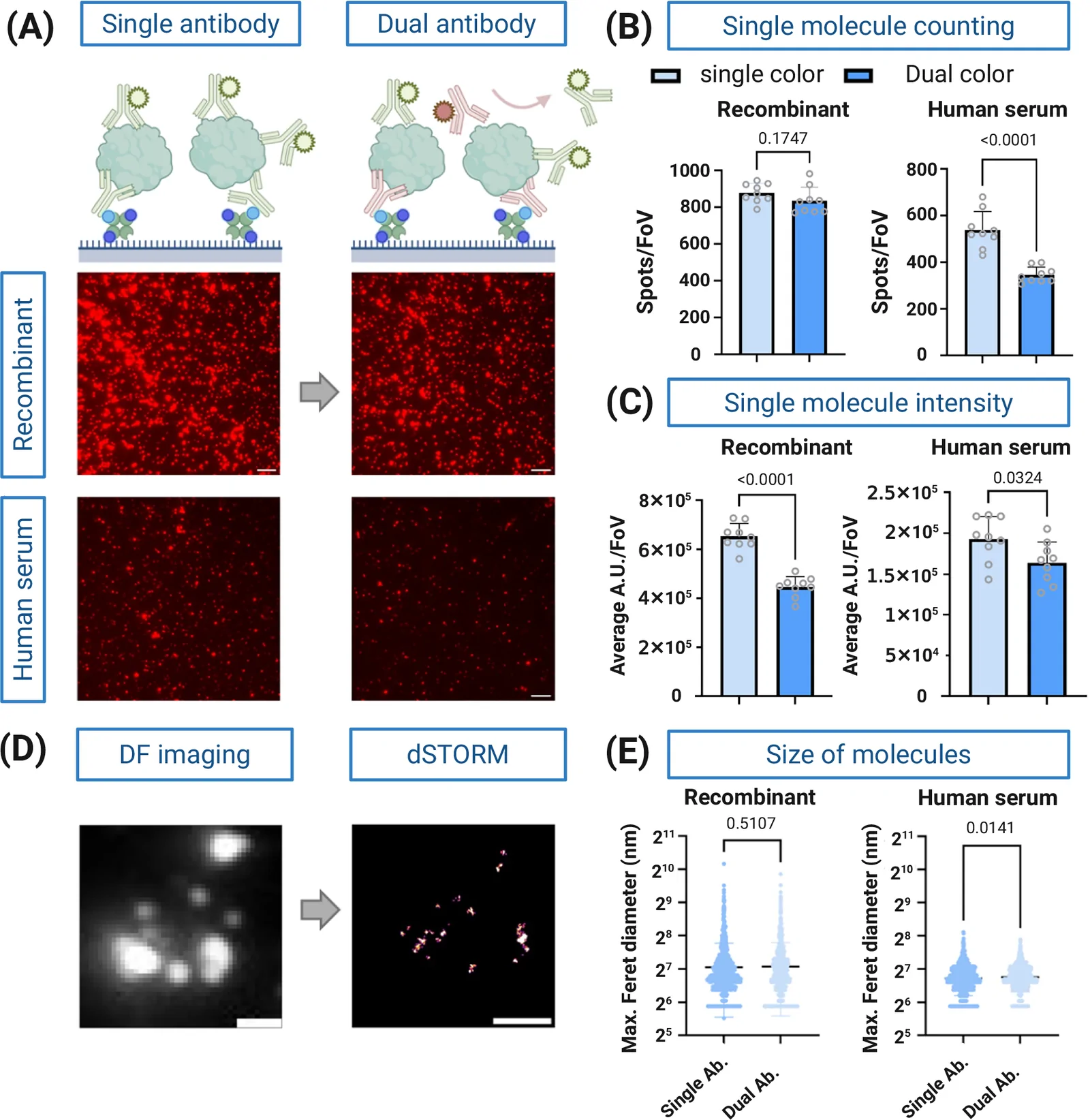
Comparison of the fluorescence from single-antibody and dual-antibody imaging of nanoscopic aggregates. Syn211(against αS) antibodies were used as capture agents (labelled with biotin). In single-antibody imaging, only Syn211-AF647 antibody was used for detection; In dual-antibody imaging, the 6E10-AF488 antibody, which binds Aβ, was also used in addition to Syn211. The illustration was generated using BioRender.com. **A** Schematic illustration demonstrating that additional detection probes can compete with primary antibodies when targeting nanoscopic structures, potentially reducing fluorescence intensity and single-molecule counts from the primary channel. Representative dual-colour images are provided in Supplementary Fig. 3. Scale bar: 5 µm. **B** Comparative single-molecule counts obtained via single- versus dual-antibody imaging conditions. Upon the addition of the 6E10 antibody, αS counts remain unchanged in in vitro co-aggregate samples but exhibit a pronounced decrease in serum samples. s.d. *n* = 9, from 9 fields of view (FoV) taken of each sample. Two-sided Welch’s t-test was used. **C** Single-molecule fluorescence intensity comparison of the primary detection antibody between single- and dual-antibody imaging regimes. A systematic decline in intensity is observed across both experimental conditions in the presence of a second antibody. s.d. *n* = 9, from 9 FoV taken of each sample. Two-sided Welch’s *t*-test was used. **D** Representative dSTORM super-resolution image and the corresponding diffraction-limited profile of recombinant αS-Aβ co-assemblies. The Syn211 antibody was used for both capture and detection. Scale bar: 10 µm. **E** Single-molecule size analysis quantified via the primary detection antibody (Syn211) under single- and dual-antibody conditions. Aggregate size was calculated using the maximum Feret diameter from super-resolved molecules^77,78^. In the recombinant group, the arithmetic mean aggregate size is 133 ± 86 nm for single-antibody imaging (*n* = 966 individual aggregates) and 134 ± 86 nm for dual-antibody imaging (*n* = 681). In the serum group, the mean aggregate size is 105 ± 31 nm for single-antibody imaging (*n* = 1122) and 108 ± 28 nm for dual-antibody imaging (*n* = 741 individual aggregates). Notably, the introduction of the co-detecting 6E10 antibody significantly shifts the size distribution profile of serum aggregates (see Supplementary Fig. 4 for cumulative size distributions; Mann-Whitney test was used due to non-Gaussian data distribution). All panels were assembled as Fig. 2 in BioRender. Zhang, Y. (2026) https://BioRender.com/znnjilz. Source data are provided as a Source Data file.

We found that additional antibodies tend to compete for binding sites, as illustrated in Fig. 2B,C. For recombinant αS-Aβ aggregates, additional detection antibody did not affect the single-molecule counting from the primary detection antibody but resulted in a ~30% reduction in intensity. This suggests that, in this case, most recombinant protein aggregates remain antibody-bound, but with a reduced number of bound antibodies per aggregate. In serum samples, the dual-antibody approach led to a decreased single-molecule counting (~35%) and intensity (~15%). Notably, the signal intensity of serum-derived αS species was roughly 30% that of recombinant αS, suggesting that fewer epitopes or binding sites are accessible. (Fig.2B, C). We also confirmed that increasing the amount of detection antibody did not increase the signal in serum, indicating that the current detection conditions are technically adequate. (Supplementary Fig. 5) This observation implies that heterotypic species with limited epitopes are less likely to be co-labelled by αS and Aβ simultaneously. Binding of Aβ antibodies can sterically hinder subsequent αS antibody binding, resulting in reduced detection counts. In contrast, larger species are still bound by antibodies, but with fewer binding events, leading to an overall reduction in signal intensity. Therefore, co-localisation is predominantly observed in larger complexes (Supplementary Fig. 3). Low co-localised signal was observed in serum. (Supplementary Fig. 3)

We then performed dSTORM super-resolution imaging to cross-validate this observation. Super-resolution analysis of recombinant αS–Aβ co-assemblies revealed no significant size differences between single- and dual-antibody imaging conditions when measured with the primary αS detection antibody. The average sizes were 133 ± 86 nm (single-antibody condition) and 134 ± 86 nm (dual-antibody condition), with both conditions showing similar size distributions. (Supplementary Fig. 4) These results suggest that, although additional detection antibodies can partially inhibit binding of the primary antibody, the remaining bound probes are still sufficient for accurate morphological mapping. The single-molecule localisation analysis, on the other hand, is consistent with intensity analysis. We found an average of 63 effective localisations per molecule in the single-antibody condition and 19 in the dual-antibody condition, where effective localisation refers to single-molecule localisations retained after removal of noise and duplicates. A higher number reflects greater antibody binding. Thus, intensity primarily reflects the number of bound antibodies rather than their spatial coverage on aggregates, which is instead indicated by the size measurement derived from super-resolution imaging.

In serum samples, the dual-antibody condition did not significantly alter the mean molecular size but modulated the size distribution detected by the primary antibody. The average aggregate sizes were 105 ± 31 nm under the single-antibody condition and 108 ± 28 nm under the dual-antibody condition, yielding approximately 9 and 10 effective localisations per molecule, respectively. These localisation frequencies represent only about 15% of those recorded for recombinant species when single-antibody condition was applied, indicating that serum-derived protein aggregates possess substantially fewer accessible epitopes, supporting previous intensity measurements. Consequently, in the presence of competing antibodies, these low-epitope serum species are more prone to escaping detection entirely rather than becoming partially labelled. The reduction in particle counts and fluorescence intensities shown in Fig. 2B and C underscores this distinct pattern of labelling loss: small aggregates with sparse binding sites drop below the detection threshold, while larger structures with a higher absolute number of bound antibodies remain detectable. This artificially shifts the dual-antibody cohort towards a larger apparent size profile (Supplementary Fig. 6). Together, these data indicate that single-molecule intensity correlates more robustly with the number of effective localisations than with physical aggregate dimensions, as both parameters fundamentally reflect probe occupancy.

### Characterisation of co-assemblies via heterologous antibody pairs

Heterologous antibody pairs targeting distinct molecular entities are an alternative strategy to characterise hetero-oligomeric co-assemblies. This approach was previously implemented to screen amylin-Aβ co-aggregates in human serum^35^.In this configuration, a specific capture antibody is immobilised onto a solid substrate, minimising competitive binding interactions with freely diffusing additional detection antibodies targeting other components of the co-assemblies. Unlike conventional multicolour imaging, which infers coexistence via the spatial colocalisation of independent fluorescent channels, this assay architecture relies on a single detection probe to inherently confirm co-assembly. However, because non-specific probe adsorption can mimic true positive signals, stringent validation is necessary to ensure that fluorescence results strictly from specific surface capture. To verify assay specificity, control experiments omitting the capture antibody confirmed the minimal background noise (Supplementary Fig. 7).

To evaluate detection specificity, this screening framework was applied to human brain tissue using a pooled homogenate derived from four subjects (*n* = 4, 2 PD and 2 controls, see SI for full information). We established a combinatorial matrix utilising three antibodies directed against αS (LB509), Aβ (6E10), and Tau (HT7), along with an isotype IgG control. Employing these reagents interchangeably as either capture or detection agents yielded 16 distinct combinations for comprehensive screening. Non-specific signals, arising from the Fc regions of detection antibodies binding to either the capture antibodies or the imaging surface, were carefully monitored using IgG isotype controls. Consistent with previous reports that background binding in complex tissue matrices is minimal on passivated surfaces^2,31,33^; we observed fewer than 20 spots per field of view (FOV) across all isotype control conditions. This confirms excellent assay specificity with negligible background interference (Supplementary Fig. 8).

For clarity, each imaging condition is described by specifying the capture and detection antibodies used. Detection antibody pairs are denoted as superscripts to the aggregate species, with the capture antibody listed first, followed by the detection antibody. For example, αS^211-211^ denotes αS aggregates imaged with both the Syn211 capture and Syn211 detection antibodies, αS-Aβ^211-6E10^ denotes αS-Aβ co-assemblies imaged with both Syn211 capture and 6E10 detection antibodies, while αS-Aβ^6E10-211^ denotes αS-Aβ co-assemblies imaged with 6E10 capture antibody and Syn211 detection antibody. This notation clarifies the specific antibody pairs applied in each condition.

**Fig. 3:**
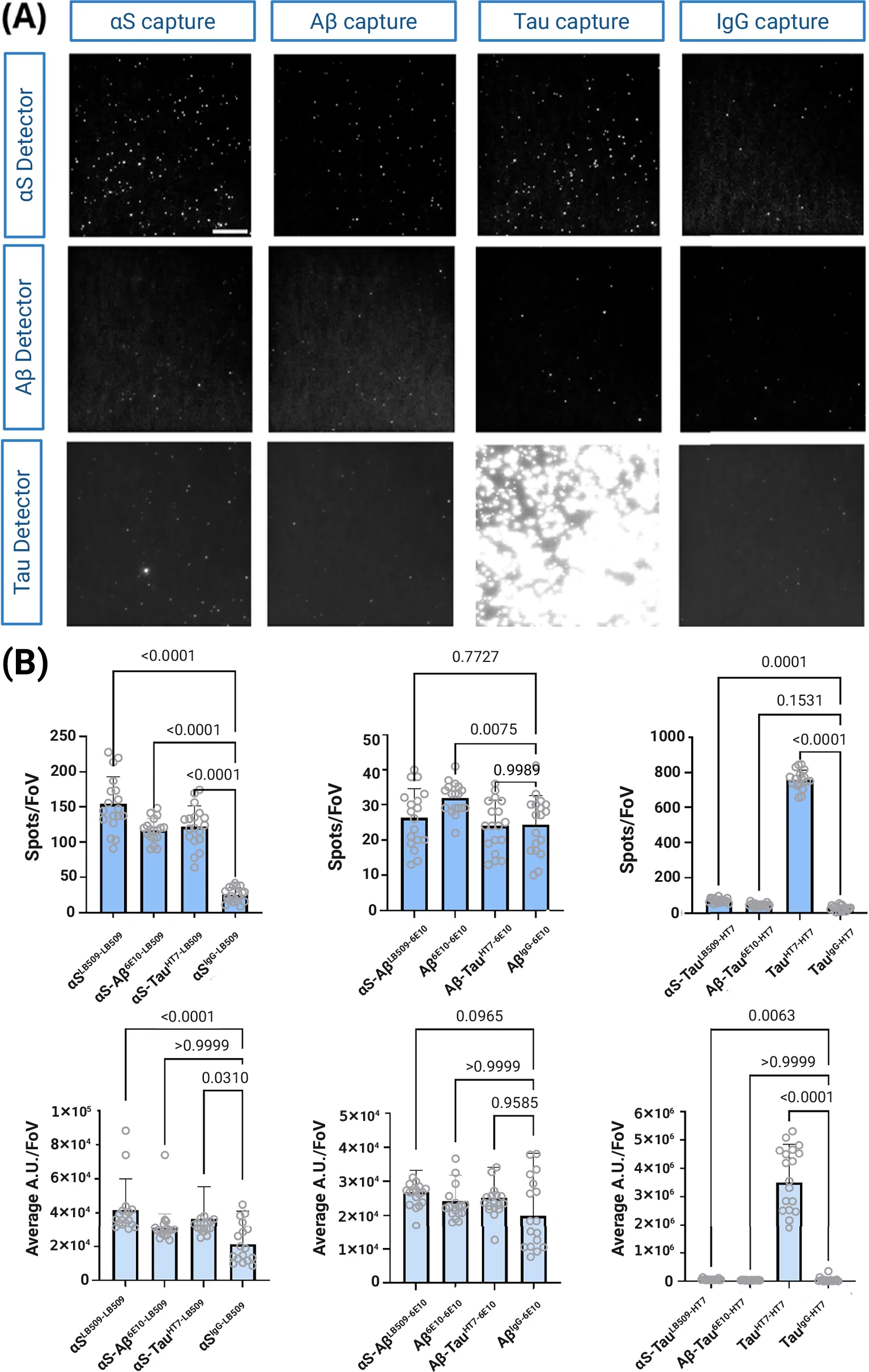
Assessment of detection specificity for co-assemblies in pooled human brain homogenate. Data were obtained from a pooled human brain homogenate sample (*n* = 4, 2 PD and 2 controls, see SI for full information). **A** Representative fluorescence images illustrating the screening outcomes across various combinatorial antibody pairs to validate assay specificity. Target-specific antibodies included LB509 (for αS), 6E10 (for Aβ), and HT7 (for Tau). The negligible signal intensities observed in the isotype IgG control channels confirm the high specificity of the detection framework. To facilitate direct comparison, display contrast was kept uniform across all panels employing the same detection probe. Due to pronounced signal saturation and resultant blurring in the original unadjusted images for the TauHT7-HT7, contrast-optimised panels for this specific group are provided in Supplementary Fig. 9. Scale bar: 10 µm. **B** Single-molecule quantification metrics, including particle counts and fluorescence intensities, derived from the indicated antibody combinations. Control conditions utilising IgG capture antibodies generated no detectable signal above background. Similarly, no significantly higher detection intensity relative to IgG controls was observed when the Aβ capture antibody was employed. Data are expressed as mean ± SD (*n* = 18 FOVs, accumulated from duplicate measurements utilising 9 FOVs per run per pooled sample). Statistical significance was evaluated using an ordinary and non-parametric one-way ANOVA with post-hoc multiple-comparison correction. See statistical method for details. All panels were assembled as Fig. 3 in BioRender. Zhang, Y. (2026) https://BioRender.com/2632ejt. Source data are provided as a Source Data file.

As illustrated in Fig. 3, a screening matrix of 12 distinct antibody combinations was evaluated. This approach aimed to isolate specific capture–detection pairings that yield single-molecule counts and fluorescence intensities significantly above the nonspecific background thresholds established by the IgG isotype controls. For species detected by αS antibodies (αS^LB509-LB509^, αS-Aβ^6E10-LB509^ and αS-Tau^HT7-LB509^), all capture antibodies demonstrated effective binding affinity except for the IgG controls (αS^IgG-LB509^). Notably, species captured using αS and Tau antibodies (αS^LB509-LB509^ and αS-Tau^HT7-LB509^) have significantly higher single-molecule intensity and counts compared to IgG controls (αS^IgG-LB509^). In contrast, αS species captured by Aβ antibodies (αS-Aβ^6E10-LB509^) displayed increased single-molecule counts but showed no difference in intensity relative to IgG controls (αS^IgG-LB509^). When visualised with Tau antibodies (αS-Tau^LB509-HT7^, Aβ-Tau^6E10-HT7^ and Tau^HT7-HT7^), species immobilised by αS and Tau capture antibodies (αS-Tau^LB509-HT7^ and Tau^HT7-HT7^) displayed distinct single-molecule characteristics, appearing with both higher counts and intensities than those captured with IgG controls (αS^IgG-HT7^). However, species visualised with Aβ antibodies (αS-Aβ^LB509-6E10^, Aβ^6E10-6E10^ and Aβ-Tau^HT7-6E10^), exhibited minimal differences across various capture conditions. These observations suggest that the detection of αS and Tau species (αS^LB509-LB509^, αS-Tau^HT7-LB509^, αS-Tau^LB509-HT7^ and Tau^HT7-HT7^) is specific and significantly different from technical controls. On the other hand, species captured and visualised by Aβ antibodies (αS-Aβ^6E10-LB509^, Aβ-Tau^6E10-HT7^, αS-Aβ^LB509-6E10^, Aβ^6E10-6E10^ and Aβ-Tau^HT7-6E10^) closely resembled IgG controls, suggesting that Aβ is less likely to form detectable co-assemblies with αS or Tau in human brain samples using this method. These findings indicate that the assay can specifically identify αS–Tau co-assemblies, as their profiles differ significantly from those of the IgG controls. We also confirmed there is no significant cross-reactivity among the antibodies tested (Supplementary Fig. 10).

### Screening αS-Tau co-assemblies in the human brain homogenate

Following the technical validation with pooled brain homogenate, we screened the αS-Tau co-assemblies present in human brain homogenates from 6 PD cases and 4 controls. Control samples were obtained from donors without neurological symptoms. We used samples derived from the amygdala, as both αS pathology and neuroinflammation in this region are closely associated with the development of PD dementia^36^. The brain tissue homogenate predominantly represents the insoluble protein fraction of the brain, as soluble fraction has been partially extracted prior to the homogenisation^2,22,37^. In addition, Tau-αS co-pathology has recently been identified in this region, in the form of large inclusions containing Tau, αS, and TDP-43^20^.

**Fig. 4:**
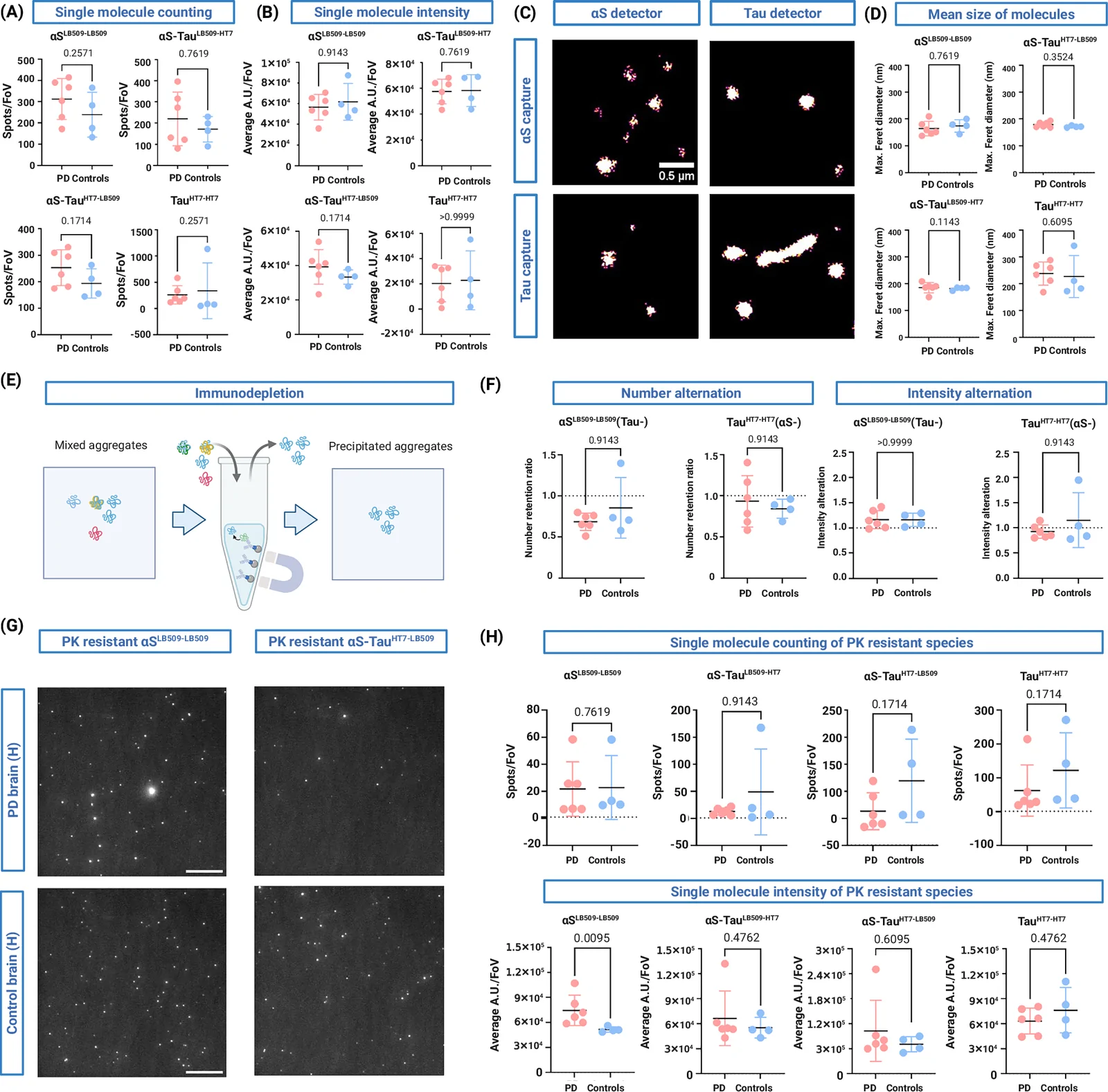
Single-molecule quantification of αS and Tau species in PD and control brain homogenate. *N* = 6 for PD, *N* = 4 for controls. **A** Single-molecule counts in PD and control brain samples demonstrate no significant differences in the concentration of the detected species between the two groups. The αSLB509-LB509, αS-Tau^HT7-LB509^, αS-Tau^LB509-HT7^, and Tau^HT7-HT7^ species are roughly ~310, 250, 220, 260 spot/FoV in PD samples, ~240, 190, 170, 340 spot/FoV in control samples. **B** Single-molecule intensity comparisons between PD and control brain samples demonstrate no significant differences in the average single-molecule intensity of the detected species between the two groups. The αS^LB509-LB509^, αS-Tau^HT7-LB509^, αS-Tau^LB509-HT7^ and Tau^HT7-HT7^ species are roughly 5 × 10^4^, 4 × 10^4^, 6 × 10^4^, 2 × 10^6^ A.U. /FoV in PD samples, 6 × 10^4^, 3 × 10^4^, 5 × 10^4^, 3 × 10^6^ A.U. /FoV in control samples. **C** Representative images of aggregates/co-assemblies characterised by different antibody pairs. Scale bar: 0.5 µm. **D** Comparison of the average size between PD and control brains. No significant difference was observed. *N* = 6 for PD, *N* = 4 for controls. **E** Schematic of immunodepletion. Magnetic beads conjugated with specific antibodies precipitate targeted species from the sample. Comparing aggregate measurements before and after immunodepletion allows quantification of aggregates associated with the corresponding protein. The illustration was generated using BioRender.com. **F** Retention of signal after immunodepletion. Tau depletion effectively reduces small αS aggregates, whereas αS depletion does not efficiently alter intensity profile of Tau. *N* = 6 for PD, *N* = 4 for controls. **G** Representative images of αS^LB509-LB509^ in brain homogenates before and after PK digestion. Bright αS^LB509-LB509^ signals derived from PD brains persist after digestion, whereas such signals are not observed in control brains. Scale bar: 10 µm. **H** Quantification of αS and Tau species in PD and control brain samples following PK digestion. Overall, no significant number differences were detected across the four assemblies. However, the intensity of αS^LB509-LB509^ aggregates was significantly higher in PD than in controls, indicating the presence of PK–resistant aggregates, a characteristic feature of αS pathology in PD. Data in (**A**, **B**, **D**, **F**, **H**) are presented as mean ± SD; each data point represents one brain donor (biological replicate;  *N* = 6 PD,  *N* = 4 control), derived from values averaged across multiple fields of view per donor (technical replicates). Non-parametric Mann-Whitney test was used for all sub-figures due to the relatively small sample size. Data are expressed as mean ± SD. All panels were assembled as Fig. 4 in BioRender. Zhang, Y. (2026) https://BioRender.com/fmrbg6i. Source data are provided as a Source Data file.

As illustrated in Fig. 4A and B, four species αS^LB509-LB509^, αS-Tau^HT7-LB509^, αS-Tau^LB509-HT7^, and Tau^HT7-HT7^, were quantified through single-molecule counting and intensity analyses. No significant differences were observed between PD and control brain homogenate when comparing these metrics. The single-molecule counts for these species were approximately 310, 250, 220, and 260 spots/FoV in PD samples, and 240, 190, 170, and 340 spots/FoV in control samples. The averaged single-molecule intensities for these species were approximately 5 × 10^4^, 4 × 10^4^, 6 × 10^4^, 2 × 10^6^ A.U. /FoV in PD samples, and 6 × 10^4^, 3 × 10^4^, 5 × 10^4^, 3 × 10^6 ^A.U. /FoV in control samples. However, when comparing assemblies captured by different antibodies but visualised with the same detection antibody (e.g., αS^LB509–LB509^ versus αS–Tau^HT7–LB509^), we observed that the αS antibody captured a greater number of αS-positive species (αS^LB509–LB509^) and produced higher intensities than those captured by Tau antibody (αS–Tau^HT7–LB509^) (Supplementary Fig. 11). This trend was evident in 5 out of 6 PD samples and 3 out of 4 control samples (Supplementary Fig. 11). Conversely, Tau species captured by Tau antibodies (Tau^HT7–HT7^) did not show higher single-molecule counts than those captured by αS antibodies (αS–Tau^LB509–HT7^) but exhibited much higher single-molecule intensities. These intensity differences were observed across all samples (Supplementary Fig. 11). Since single-molecule intensity profiles typically do not follow Gaussian distribution, averaged metrics are insufficient to fully characterise these populations^38^. We found that 25%-40% of the total fluorescent intensity was contributed by the brightest 5% of molecules (Supplementary Fig. 12). To address this potential bias, we plotted cumulative histograms of single-molecule intensities^2,22^ (Supplementary Fig. 13). PD samples contained a greater proportion of dim αS^LB509–LB509^ molecules (A.U. < 1.3 × 10^5^), but a higher proportion of bright Tau^HT7–HT7^ molecules (A.U. > 4 × 10^6^). No clear differences were observed between PD and control groups for αS–Tau^HT7–LB509^. We also performed similar measurement with amygdala brain homogenate from AD and PSP patients, revealing that co-assemblies are also detectable in tauopathy samples. Notably, when combining the intensity profiles of αS–Tau^LB509–HT7^ and Tau^HT7–HT7^, three sample groups (PSP, AD and controls) could be distinguished from each other (Supplementary Fig. 14): higher αS–Tau^LB509–HT7^ intensity separated AD and PSP from controls, while Tau^HT7–HT7^ intensity was greater in AD than in PSP.

To further characterise these aggregates, we employed dSTORM super-resolution imaging (Fig. 4C, D). While intensity data provide valuable insights into the number of antibodies bound to targets, they do not directly capture the spatial distribution of antibodies on aggregates, as discussed in Fig. 2. Our analysis revealed no significant difference in the mean size of aggregates between PD and control brain samples. The measured sizes for the four species, αS^LB509-LB509^, αS-Tau^HT7-LB509^, αS-Tau^LB509-HT7^, and Tau^HT7-HT7^, were 164 ± 24 nm, 178 ± 9 nm, 185 ± 17 nm, and 238 ± 24 nm in the PD group, and 174 ± 20 nm, 171 ± 3 nm, 182 ± 5 nm, and 227 ± 68 nm in the control group, respectively. However, cumulative histograms revealed trends similar to the intensity data: PD samples contained a higher proportion of smaller αS^LB509-LB509^ molecules (100–300 nm) and larger Tau^HT7-HT7^ aggregates (200–400 nm) (Supplementary Fig. 13). Interestingly, the average size of Tau^HT7–HT7^ species was approximately 30% larger than that of αS–Tau^LB509–HT7^, yet their intensity was more than 10-fold higher. This suggests that Tau^HT7–HT7^ species possess a higher density of accessible binding epitopes, indicating that they are substantially richer in Tau subunits compared to αS–Tau^LB509–HT7^. A similar pattern was observed for αS species: although αS^LB509–LB509^ and αS–Tau^HT7–LB509^ were comparable in size, αS^LB509–LB509^ exhibited ~50% higher intensity, indicating a greater density of αS subunits.

While our capture strategy enables the characterisation of heterotypic assemblies, it remains challenging to distinguish between small complexes (e.g. one αS and one Tau subunit) and larger aggregates, as the surface captures any species containing at least one marker molecule. To address this, we applied the immunodepletion strategy coupled with aggregate-specific imaging to quantify heterotypic aggregate species^39^. Aggregate-specific imaging employs antibodies targeting identical epitopes for both capture and detection, thereby visualising only aggregated species containing more than two epitopes^2,22^. Quantification of aggregate species before and after immunodepletion allows estimation of the relative abundance of larger co-assemblies containing multiple αS and Tau subunits. This approach provides a complementary method to the direct characterisation of heterotypic assemblies using heterologous capture–detection antibody pairs that target different species: immunodepletion provides a lower-limit estimate of the proportion of co-assemblies, while direct characterisation represents an upper-limit estimate, as it may include small assemblies that are not sufficiently large to be considered true aggregates.

Control experiments were performed using IgG-coupled beads to verify the specificity of the immunodepletion (Supplementary Fig. 15)^39^. As shown in Fig. 4F, Tau depletion effectively reduces the number of αS aggregates, with 9 out of 10 samples exhibiting fewer aggregates and increased mean intensity following Tau removal. These findings indicate that Tau is associated with nanoscopic αS aggregates in brain homogenates, in particular those with a limited number of αS subunits. The increased intensity represents the removal of these species. Notably, this association does not appear to be disease-specific, as there was no difference in signal retention between PD and control samples following depletion. The median molecule retention rate was approximately 70% for both PD and control samples, indicating that roughly 30% of αS aggregates are associated with Tau in brain homogenates. Conversely, αS depletion induced only minor changes in the number of Tau aggregates, with approximately 90% of molecules retained following depletion in both PD and control samples. Furthermore, αS depletion did not alter the intensity profile of Tau aggregates, suggesting that αS does not associate with Tau aggregates of certain sizes present in brain homogenates. The paired comparison further illustrated this trend (Supplementary Fig. 16).

Following these imaging-based characterisations, we assessed the PK resistance of aggregates (Fig. 4G, H) a key characteristic of PD-derived αS^40,41^. Although the overall number of αS^LB509–LB509^ post-digestion did not differ clearly between PD and control samples, brighter αS^LB509–LB509^ aggregates remained in the PD samples after PK treatments (Fig. 4G). However, this disease-specific feature was not observed for heterotypic assemblies or Tau aggregates, indicating there is a structural difference between αS-containing heterotypic assemblies and disease-specific αS aggregates. Because αS^LB509–LB509^ species did not exhibit disease-specific features prior to PK digestion, they likely represent a heterogeneous population of complexes with varying structures and compositions, wherein only the PK-resistant subsets reflect the disease-specific fraction. Notably, the data shown in Fig. 4A–C and Fig. 4F–H were collected from two independent measurements conducted 15 months apart. To ensure comparability, we performed a correlation analysis between the two datasets (Supplementary Fig. 17). The strong correlation observed confirms the reproducibility of the measurements and suggests that the protein co-assemblies are stable over time.

### Screening αS-Tau co-assemblies in the soluble fraction of brain-derived proteins

Soluble protein aggregates, particularly low-molecular-weight oligomeric species, are widely considered to be highly cytotoxic and potent drivers of neuroinflammation^2,22,42^. To further investigate these species, we characterised the soluble protein fraction from an independent, expanded cohort of human brain tissue, comprising 9 controls and 8 PD cases. Following tissue homogenization, the soluble fraction was isolated via ultracentrifugation at 100,000 × *g* for 1 h at 4 °C. For the single-molecule imaging analysis of this cohort, an optimised protocol was implemented, successfully achieving an enhanced signal-to-background ratio by further minimising non-specific background fluorescence (Supplementary Fig. 18).

**Fig. 5:**
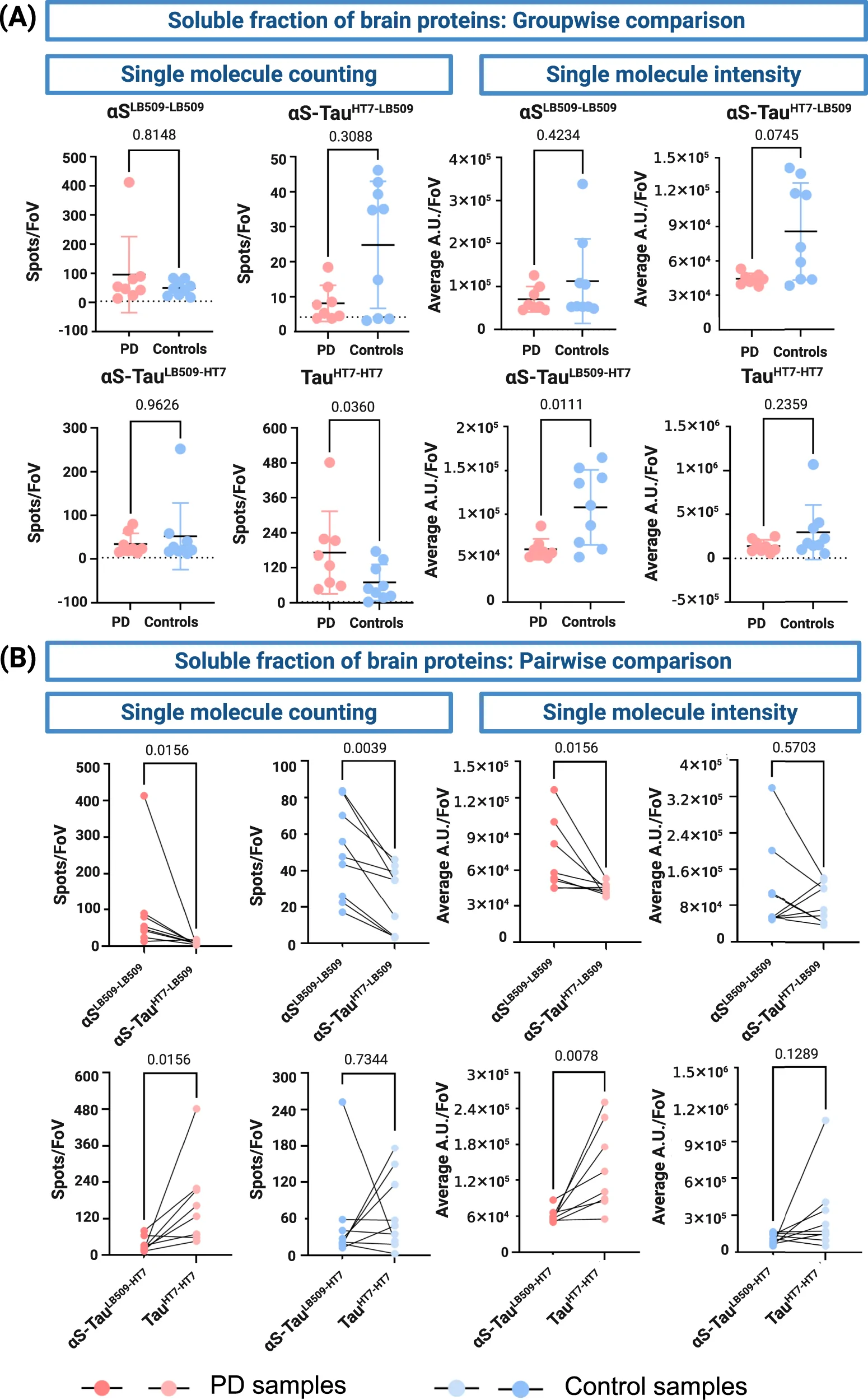
Single-molecule quantification of αS and Tau species in the soluble protein fraction of PD and control brains. *N* = 8 for PD, *N* = 9 for controls. **A** Single-molecule counts in PD and control brain samples indicate that PD brains contain more soluble Tau aggregates. Single-molecule intensities reveal that αS–Tau^LB509–HT7^ species in control brains have higher fluorescent intensity than in PD brains. A non-parametric Mann-Whitney test was used. **B** Paired comparison of protein assemblies captured by different antibodies but visualised with the same detection antibodies. PD samples show more distinct heterotypic assemblies. Both αS^LB509–LB509^ and Tau^HT7–HT7^ exhibit higher single-molecule counts and intensities than αS–Tau^HT7–LB509^ and αS–Tau^LB509–HT7^, respectively. Control samples display less distinct heterotypic assemblies, with only the number of αS–Tau^HT7–LB509^ species being lower than αS^LB509–LB509^. Each data point represents one brain donor (biological replicate; *N* = 8 PD, *N* = 9 control). A paired Wilcoxon test was used for statistics. All panels were assembled as Fig. 5 in BioRender. Zhang, Y. (2026) https://BioRender.com/a6x8sr9. Data are expressed as mean ± SD. Source data are provided as a Source Data file.

As shown in Fig. 5, assemblies in the soluble fraction exhibit more disease-specific features than those in homogenates. Four species, αS^LB509–LB509^, αS–Tau^HT7–LB509^, αS–Tau^LB509–HT7^, and Tau^HT7–HT7^, were quantified by single-molecule counting and intensity analysis. In PD samples, single-molecule counts were approximately 100, 10, 30, and 170 spots per FoV, respectively, compared with 50, 20, 50, and 70 spots per FoV in control samples. Average single-molecule intensities in PD samples were ~7 × 10⁴, 4 × 10⁴, 6 × 10⁴, and 1 × 10⁵ A.U./FoV, compared with 1 × 10⁵, 8 × 10⁴, 1 × 10⁵, and 3 × 10⁵ A.U./FoV in controls. Overall, the soluble fraction contained a relatively low abundance of protein assemblies, with αS–Tau^HT7–LB509^ species approaching the detection limit in approximately 30% of samples. Moreover, the intensity of Tau^HT7–HT7^ in homogenates is approximately 10-fold higher than in the soluble fraction, suggesting that Tau aggregates in the soluble fraction differ from those in homogenates. Notably, αS–Tau^LB509–HT7^ species in control brains displayed higher intensities than in PD brains.

**Fig. 6:**
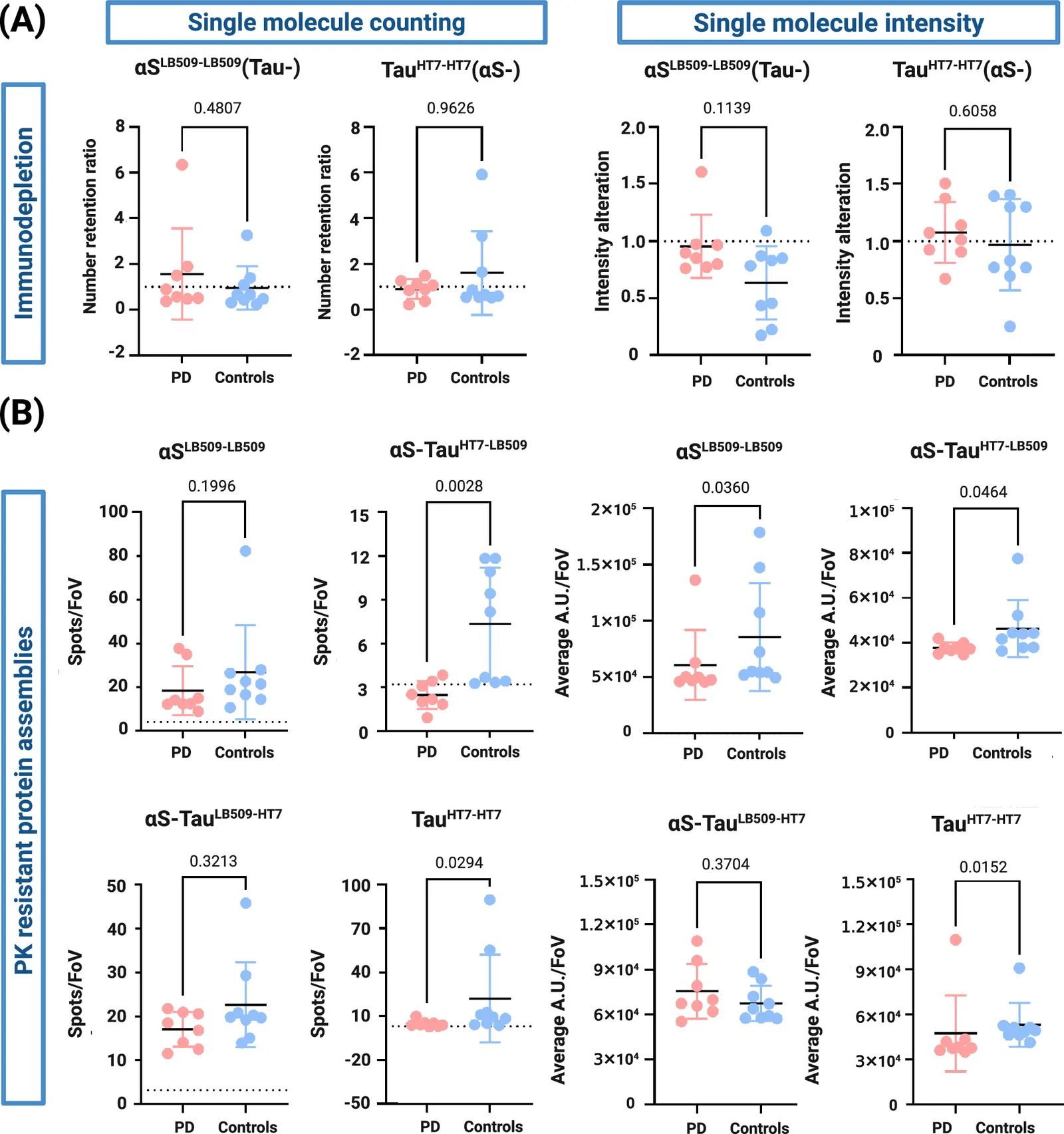
Quantification of αS–Tau co-aggregates and PK-resistant assemblies in the soluble protein fraction. **A** Immunodepletion analysis. Magnetic beads coupled to αS or Tau antibodies precipitated the corresponding species, and αS/Tau aggregate levels were quantified before and after depletion to estimate co-aggregate content. Signal retention varied across samples, with some showing minimal reduction after depletion. No significant differences were observed between the PD and control groups. **B** Quantification of PK-resistant assemblies. Control samples retained higher numbers of αS–Tau^HT7-LB509^ and Tau^HT7-HT7^ species after digestion, and showed elevated intensities for αS^LB509^^-^^LB509^, αS–Tau^HT7-^^LB509^, and Tau^HT7-HT7^ compared with PD. By contrast, most αS–Tau^HT7-^^LB509^ and Tau^HT7-HT7^ signals in PD samples dropped below the detection limit following digestion. Each data point represents one brain donor (biological replicate;  *N* = 8 PD, *N* = 9 control, matching the soluble-fraction cohort in Fig. 5). Non-parametric Mann-Whitney test was used for all sub-figures due to the relatively small sample size. Data are expressed as mean ± SD. All panels were assembled as Fig. 6 in BioRender. Zhang, Y. (2026) https://BioRender.com/huaeme9. Source data are provided as a Source Data file.

Following the groupwise analysis, we conducted a pairwise comparison of the distinct assemblies detected within individual samples (Fig. 5B). This analysis revealed that PD samples harboured heterotypic assemblies with features that were distinctly different from the general aggregate population. Specifically, αS^LB509–LB509^ and Tau^HT7–HT7^ yielded significantly higher single-molecule counts and fluorescence intensities than heterotypic αS–Tau^HT7–LB509^ and αS–Tau^LB509–HT7^. Notably, control samples exhibited less pronounced differentiation amongst heterotypic assemblies; only the abundance of the αS–Tau^HT7–LB509^ species was significantly reduced compared to the αS^LB509–LB509^ baseline. (Fig. 5B) In contrast to the findings from whole tissue homogenates, immunodepletion of the soluble fraction did not significantly modulate the total number of detected protein aggregates (Fig. 6A). The singular exception occurred in control samples, where a reduction in αS^LB509–LB509^ intensity was seen following Tau depletion (Supplementary Fig. 19). This negligible overall shift in the aggregate population following immunodepletion indicates that heterotypic assemblies within the soluble fraction exist predominantly as small, discrete species with limited binding epitopes, rather than the larger, multivalent inclusions characteristic of homogenates.

The PK digestion assays further elucidated disease-specific resistance profiles among soluble protein assemblies (Fig. 6B). Unexpectedly, protein assemblies in control brains showed greater resistance to PK digestion than PD. Control samples retained higher numbers of αS–Tau^HT7–LB509^ and Tau^HT7–HT7^ species after digestion. Control samples also displayed higher intensities for αS^LB509–LB509^, αS–Tau^HT7–LB509^, and Tau^HT7–HT7^ compared with PD samples. By contrast, most αS–Tau^HT7–LB509^ and Tau^HT7–HT7^ signals in PD samples fell below the detection limit following digestion. The improved surface chemistry suppresses the non-specific antibody binding, making it possible to quantify samples with low number of a single molecules (Supplementary Fig. 20).

Notably, the PK resistance profile of αS^LB509–LB509^ species in the soluble fraction diverged from that previously observed in brain homogenates. Similarly, the disease-specific PK resistance patterns for αS–Tau^HT7–LB509^ and Tau^HT7–HT7^ identified in the soluble fraction were also absent in homogenate samples. Although the PK resistance of αS is classically considered a hallmark of Lewy bodies, recent studies indicate that the clearance of PK-resistant αS does not correlate with neuronal survival in viral vector-based PD mouse models^43^. Consequently, the elevated intensity of PK-resistant αS^LB509–LB509^ species in control brains implies that these structures may not be the primary toxic assemblies driving PD pathogenesis. Furthermore, while PD samples initially harboured more Tau^HT7–HT7^ species than controls prior to digestion, this trend inverted post-digestion. This dynamic suggests that the amygdala in PD brains contains a higher abundance of comparatively unstable, soluble Tau aggregates than the amygdala of healthy controls.

### Screening co-assemblies in human biofluids

Although brain-derived pathological protein aggregates are recognised as hallmarks of various neurodegenerative diseases, it is widely reported that protein aggregates are also present in other biofluids, including serum, plasma, CSF, and saliva^28,29,33,39^. Unlike brain homogenates, these biofluids are directly linked to the circulatory system and contain a higher abundance of biomolecules, which may introduce additional interference in measurement assays. To further validate our observations, we extended the study by incorporating two additional antibodies, 4G8 and Tau5. The analysis specifically focused on αS antibody-captured species, as Tau-capture antibodies generally yielded lower signal intensities in human serum samples: the number of Tau aggregates in serum was approximately 20% of the molecules captured by the αS antibody pair (see Supplementary Fig. 21).

Initial technical controls were performed to evaluate the specificity of co-assembly detection in human serum. The assay successfully identified species reactive to αS, Aβ, or Tau detection antibodies following immobilisation with an αS capture antibody. Crucially, omitting the capture antibody yielded a negligible signal (Supplementary Fig. 22), confirming the high specificity of the assay. Furthermore, while the passivated imaging surface does not inherently promote the non-specific binding of protein aggregates, intrinsic components of complex biological matrices can still adhere to the substrate. This non-specific adherence can interfere with the photophysics of adjacent fluorescent probes, potentially introducing bias into intensity quantification^44^. To address this, we systematically investigated the impact of various biological matrices on fluorophore photophysics (Supplementary Fig. 23). Amongst the matrices tested, brain homogenate exhibited minimal interference; conversely, serum, saliva, and CSF significantly perturbed fluorescence intensity. Consequently, intensity-based quantitative analyses were excluded for these specific biofluids.

**Fig. 7:**
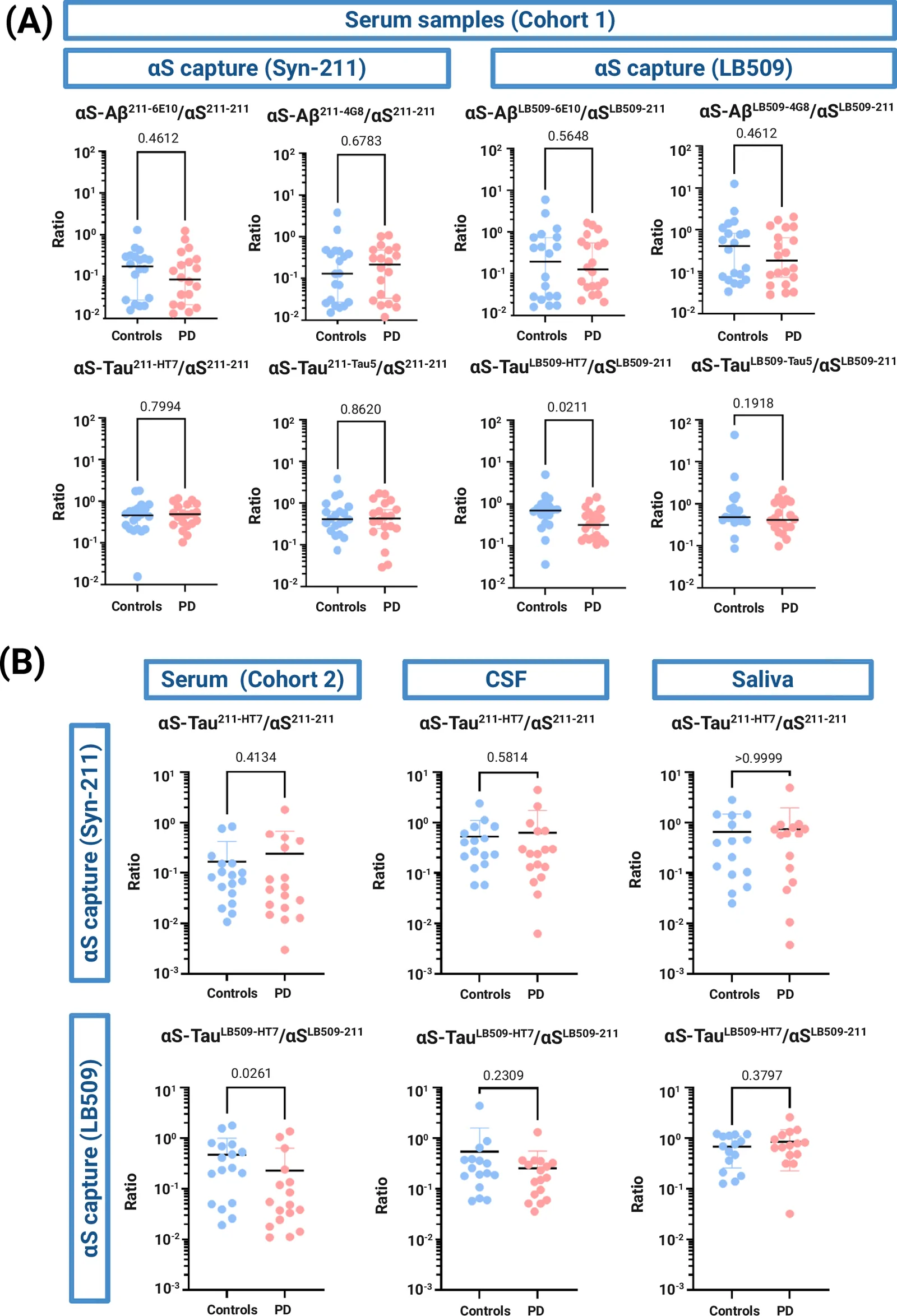
Quantification of co-assemblies in human biofluids. **A** The ratio between the number of co-assemblies and general aggregates. (Cohort 1, 20 PD and 20 control serum) The ratio was determined by comparing the single-molecule counts of species characterised by different antibody pairs. Two capture antibodies (Syn211 and LB509) and five detection antibodies (Syn211, 4G8, 6E10, HT7, and Tau5) were used. Tau was found to have a higher tendency to form co-assemblies with αS compared to Aβ. Most measurements did not display disease specificity, except for the αS–Tau^LB509–HT7^. Two-sided Mann-Whitney test was used due to the non-Gaussian distribution of these datasets. **B** The ratio between the number of αS-Tau co-assemblies and general αS aggregates in different biofluids. For serum samples, *N* = 17 PD and *N* = 17 control. For CSF samples, *N* = 17 PD and *N* = 16 control. For saliva samples, *N* = 15 PD and *N* = 15 control. Two capture antibodies (Syn211 and LB509) and two detection antibodies (Syn211 and HT7) were used in this experiment. No significant disease specificity was observed in CSF or saliva samples. Two-sided Mann-Whitney test was used due to the non-Gaussian distribution of these datasets. Data are presented as median values. All panels were assembled as Fig. 7 in BioRender. Zhang, Y. (2026) https://BioRender.com/q39n507. Source data are provided as a Source Data file.

Two human serum cohorts were analysed: cohort 1 (20 PD, 20 control) and cohort 2 (17 PD, 17 control). The first cohort was used to test different antibody pairs and assess potential disease specificity. Ten antibody combinations were applied to examine αS–Aβ and αS–Tau co-assemblies, and all conditions yielded detectable signals (Supplementary Fig. 24). In contrast to brain homogenate, species captured by αS antibodies in serum could be detected using Aβ antibodies in addition to αS and Tau probes (Fig. 7). This indicates that blood harbours a broader spectrum of co-assemblies than the brain, suggesting that some of these structures may form peripherally.

To estimate the likelihood of αS forming co-assemblies, we calculated the ratio between co-assemblies and general αS aggregates. On average, αS–Aβ species (αS–Aβ^211–6E10^, αS–Aβ^211–4G8^, αS–Aβ^LB509–6E10^, αS–Aβ^LB509–4G8^) accounted for ~10% of general αS aggregates (αS^211–211^, αS^LB509–211^), while αS–Tau species (αS–Tau^211–HT7^, αS–Tau^211–Tau5^, αS–Tau^LB509–HT7^, αS–Tau^LB509–Tau5^) accounted for 10-40%. Limited disease specificity was observed overall, with the exception that the ratio of αS–Tau^LB509–HT7^ to αS^LB509–211^ (αS–Tau^LB509–HT7^/αS^LB509–211^) was significantly higher in controls, suggesting that αS in serum from controls may have a greater tendency to co-assemble with Tau.

To further evaluate this potential disease specificity, we examined a second cohort (*n* = 17 PD, *n* = 17 controls). Similar trends were observed, with a higher ratio of αS–Tau^LB509–HT7^ to αS^LB509–211^ in controls. No differences were detected when Syn211 was used for capture. However, the ratio of αS–Tau^LB509–HT7^ to αS^LB509–211^ was lower in this cohort, at approximately 10%. Co-assemblies were also detectable in CSF and saliva, but without clear disease-specific patterns. Importantly, the ratio of co-assemblies was antibody-dependent: LB509 captured more co-assemblies in serum, whereas syn211 performed better in CSF. In saliva, both antibodies performed similarly, capturing comparable levels of co-assemblies in PD and controls (Supplementary Fig. 25).

The serum data presented in Fig. 7 were acquired from samples diluted in PBST. Heterophilic antibodies (HA) present in human biofluids, particularly serum and plasma, can potentially interfere with such measurements^45,46^. While some assays with diagnostic potential for PD include HA blockers in the diluent^47,48^, there are also reports suggesting PBS alone is sufficient for detecting disease-related αS species^30,39,49,50^. To evaluate whether HA-associated interference affected serum SiMPull measurements, we assessed nonspecific IgG binding under αS-capture conditions in the absence or presence of an HA blocker (Supplementary Fig. 26). In the PBST-only condition, approximately 40% of serum samples showed relatively high IgG-control signals (>100 spots/FoV), with about half of these exceeding 200 spots/FoV. Nevertheless, αS–Tau^LB509–HT7^ signals were significantly higher than the corresponding IgG detection controls under this condition, supporting detectable αS–Tau signal above nonspecific IgG background.

Because the serum SiMPull readouts were highly skewed, we further analysed these data using an Aligned Rank Transform (ART) ANOVA with Detector (HT7 versus IgG) and Blocker (PBST versus HA blocker) as the two factors^51,52^. This analysis was used to test whether HA blocker selectively improved the αS–Tau signal relative to the IgG background while preserving the factorial structure of the experiment. The analysis showed significant Detector and Blocker effects, indicating that HT7 signals were higher than IgG controls overall and that HA blocker significantly altered the serum SiMPull readout. However, the Detector × Blocker interaction was not significant, indicating that HA blocker did not selectively improve the HT7 vs. IgG signal differential.

Aligned Rank Transform Contrasts (ART-C) post-hoc comparisons further showed that αS–Tau^LB509–HT7^ signals remained significantly higher than IgG controls under both PBST-only and HA-blocked conditions. Thus, the αS–Tau signal is unlikely to be attributed solely to nonspecific antibody binding under PBST-only conditions. However, elevated IgG-control signals in a subset of PBST-only serum samples indicate that non-specific matrix effects still contribute to the assay readout. (Supplementary Fig. 26) Although the addition of an HA blocker reduced background IgG reactivity, which suggests improved assay stringency, it concomitantly diminished the specific αS–Tau^LB509–HT7^ signal, lowering the median by approximately 60%. Consequently, HA blocking did not yield a net enhancement of the detectable αS–Tau signal; rather, it indiscriminately suppressed both the background and the true positive signal.

**Fig. 8:**
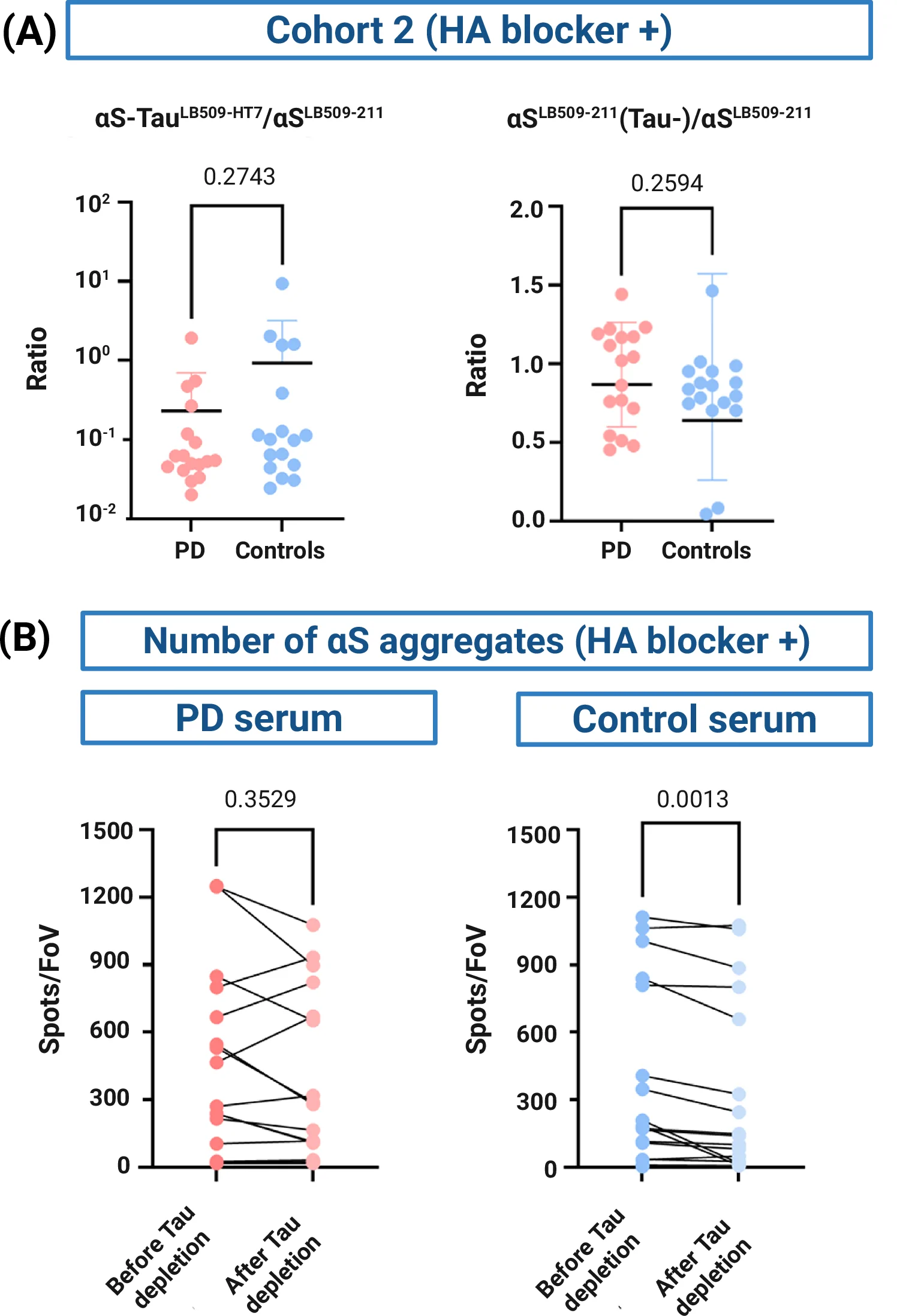
Direct and indirect quantification of αS–Tau co-assemblies in human serum diluted with heterophilic blockers. **A** Direct comparison of the αS–Tau^LB509–HT7^/αS^LB509–211^ ratio and αS^LB509–211^(Tau-)/αS^LB509–211^ between PD and control samples. Non-parametric Mann-Whitney test was used due to non-Gaussian sample distribution. Data are expressed as mean ± SD. **B** Paired comparison of single-molecule counts of αSLB509–211 before and after Tau depletion in individual samples. *N* = 17 for both PD and controls. A paired Wilcoxon test was applied for statistics. All panels were assembled as Fig. 8 in BioRender. Zhang, Y. (2026) https://BioRender.com/eq3jgxi. Source data are provided as a Source Data file.

This signal reduction is consistent with additional experiments showing that HA blocker can suppress not only nonspecific IgG binding but also specific antigen–antibody interactions, with up to a 50% reduction in signal even when recombinant αS was the only target present (Supplementary Fig. 27). Thus, in our SiMPull set-up, HA blocking does not simply remove background noise; it also introduces a loss of analytical sensitivity, likely through excessive blocking or interference with specific binding events. With this sensitivity–stringency trade-off, disease-associated differences between αS–Tau^LB509–HT7^ and αS^LB509–211^ signals in PD and control serum samples were no longer detectable when the HA blocker was applied (Fig. 8A). These findings suggest that HA blocking provides a more stringent but less sensitive serum SiMPull condition by reducing nonspecific serum-associated signal while attenuating part of the detectable specific signal.

We further cross-validated the SiMPull observations by performing immunodepletion experiments. As shown in Fig. 8(B), the post-depletion signals were heterogeneous, similar to the pattern observed in the soluble brain fraction. This heterogeneity is consistent with the possibility that detectable αS–Tau-associated species in serum and soluble brain-derived fractions have variable epitope availability. In the presence of the HA blocker, Tau antibody-coated beads significantly reduced the detectable αS signal in control samples, supporting the presence of αS–Tau co-assemblies. This reduction was less consistent in PD samples. Unexpectedly, some PD samples showed an increased αS signal after Tau immunodepletion. This increase may reflect altered epitope accessibility, release from interfering complexes, or serum matrix effects after depletion, rather than an increase in total αS abundance. When the pre- and post-depletion signal ratios were compared across groups, no clear disease-specific depletion pattern was observed. Thus, the immunodepletion data support the presence of a measurable target-associated component in serum, but also indicate that serum matrix effects and epitope accessibility can influence the apparent SiMPull signal.

### Tau modulates αS aggregation at the nanoscopic scale in a context-dependent manner

We next investigated how the formation of αS–Tau co-assemblies relates to overall aggregation of αS. Although previous studies have shown that αS and Tau can promote each other’s aggregation, most observations were from in vitro systems^16,53,54^. Such two-protein systems may oversimplify the complex brain environment, where abundant proteins such as albumin can modulate aggregation^55,56^. To address this, we tested αS aggregation (0.5 mg/ml) under five conditions: addition of Tau (0.05 or 0.01 mg/ml), addition of bovine serum albumin (BSA) (1 mg/ml), or addition of BSA (1 mg/ml) together with Tau (0.05 or 0.01 mg/ml). BSA was included as a model abundant, soluble, non-amyloidogenic albumin-like background protein to evaluate whether Tau-enhanced αS aggregation is preserved in a protein-rich environment. This choice was motivated by previous studies showing that albumin, the most abundant protein in human serum, can interact with αS and modulate its aggregation in a buffer- and concentration-dependent manner^56^. Human serum albumin (HSA) has been reported to slow αS aggregation at serum concentrations, whereas BSA, which shares substantial sequence similarity with HSA, has also been used in prior studies examining albumin-mediated effects on αS fibrillization^56,57^.

For simplicity, conditions with 0.05 mg/ml Tau are denoted as Tau(H), and those with 0.01 mg/ml Tau as Tau(L), where H and L indicate high and low concentrations, respectively. Samples were incubated at 37 °C for ~80 h, and 0N4R Tau^23^ was used.

**Fig. 9:**
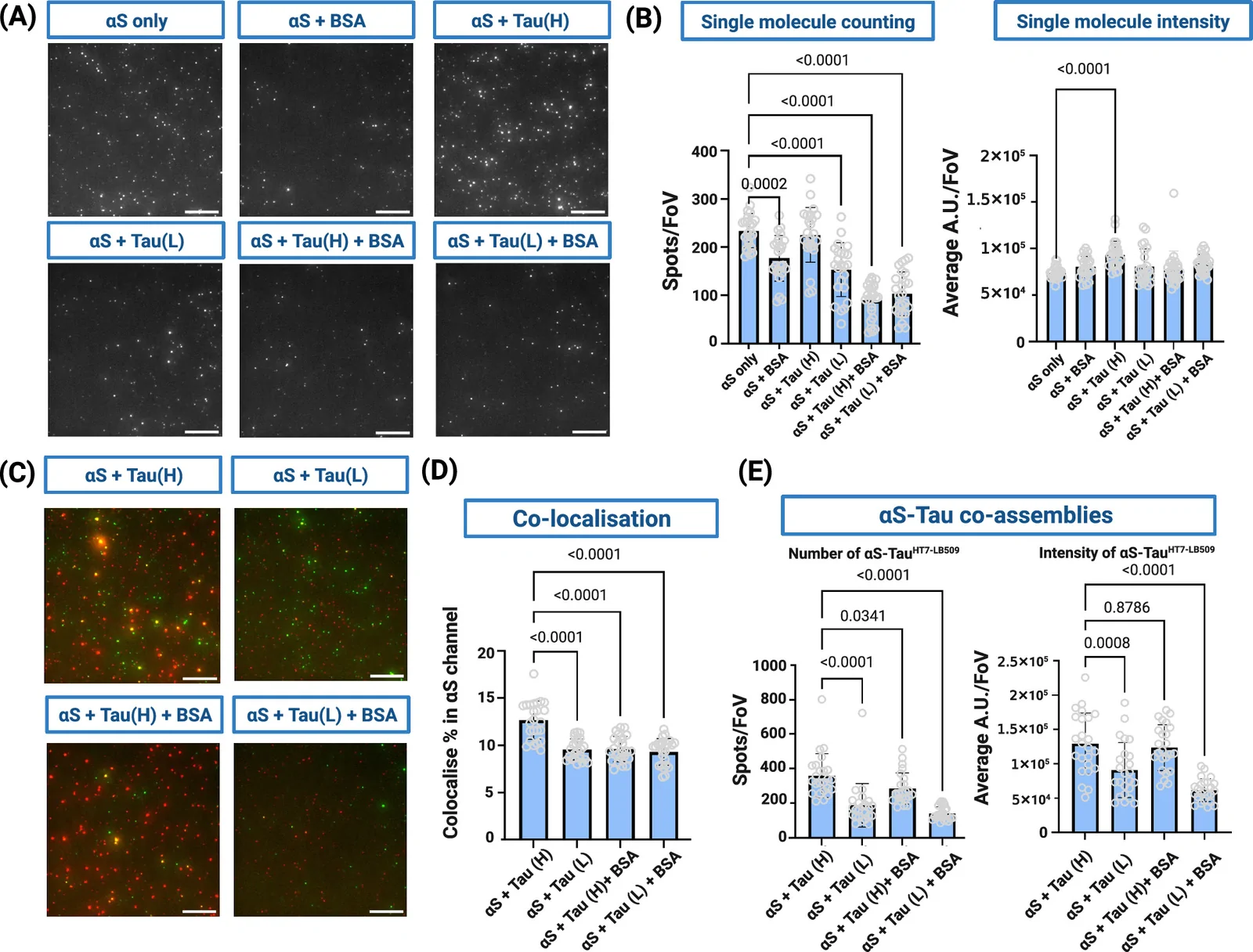
Single-molecule imaging of recombinant αS aggregates under different conditions. **A**. Representative single-molecule images of recombinant αS aggregates formed under six conditions: αS only; αS with Tau (0.05 or 0.01 mg/ml); αS with BSA (1 mg/ml); or αS with BSA plus Tau (0.05 or 0.01 mg/ml). Scale bar, 10 μm. **B** Quantification of general αS aggregates. The addition of Tau at 0.05 mg/ml produced higher mean single-molecule fluorescence intensity without reducing single-molecule counts (s.d. *n* = 24, 24 FoV from two independent experiments). One-way ANOVA was used for statistical test. **C** Representative dual-colour images of αS aggregates (green) and αS–Tau co-assemblies (red). Scale bar, 10 μm. **D** Quantification of αS–Tau co-localisation. Only αS with 0.05 mg/ml Tau showed an increased co-localisation rate, consistent with a stronger αS–Tau interaction. (s.d. *n* = 24, 24 FoV from two independent experiments). One-way ANOVA was used for statistical test. **E** Quantification of αS-Tau co-assemblies. Under the high-Tau condition, addition of BSA significantly reduced the number of αS–Tau co-assemblies by approximately 20% but did not significantly alter their mean fluorescence intensity. (s.d. *n* = 24, 24 FoV from two independent experiments). Ordinary One-way ANOVA with post-hoc multiple-comparison correction was used for statistical test. All panels were assembled as Fig. 9 in BioRender. Zhang, Y. (2026) https://BioRender.com/7iv8482. Source data are provided as a Source Data file.

Thioflavin T(ThT) fluorescence indicated that only αS with 0.05 mg/ml Tau formed ThT-positive species (Supplementary Fig. 28). However, because BSA itself produced strong ThT positivity, ThT assays could not reliably assess αS or Tau aggregation in these conditions^58^. To overcome this limitation, we applied aggregate-specific SiMPull to quantify αS assemblies. The single-molecule imaging indicates that the aggregates formed remain at the nanoscopic scale, rather than developing into micron-scale fibrillar structures. (Fig. 9A) Compared to αS alone, the αS + Tau (H) condition yielded a similar aggregate count but significantly higher averaged fluorescence intensities (Fig. 9B). This indicates that Tau heterogeneously accelerates αS growth within specific aggregate subpopulations rather than promoting overall nucleation. Conversely, lower Tau concentrations or Tau–BSA mixtures produced fewer aggregates, none of which exceeded the intensity of αS-only condition. Interestingly, the αS + Tau (H) + BSA condition exhibited the strongest suppression of αS aggregation, resulting in an approximate 60% reduction in aggregate count. Quantitative super-resolution imaging confirmed that αS aggregates formed under αS + Tau (H) condition were physically larger than those formed by αS alone (Supplementary Fig. 29). Interestingly, assemblies formed under αS + Tau (H) + BSA conditions matched the larger physical size of αS + Tau (H) yet exhibited fluorescence intensities comparable only to αS alone. This dissociation between physical size and signal intensity suggests that BSA induces less compact aggregate structures, expanding aggregate dimensions without increasing accessible αS subunit exposure. Together, these observations demonstrate that Tau heterogeneously accelerates αS aggregation, while complex protein environments like BSA alter structural aggregate packing.

We further performed dual-colour imaging to characterise αS–Tau co-assemblies. These assemblies were large enough to produce co-localised signals. (Fig. 9C) A higher co-localisation rate was observed only in the αS + Tau (H) condition, indicating stronger αS–Tau interactions. Notably, the addition of BSA did not significantly alter the intensity of αS–Tau co-assemblies, resulting in only a minor reduction (~20%) in their overall count. (Fig. 9E) In contrast, this suppressive effect was far more pronounced for the formation of general αS aggregates, which experienced a 60% decrease in number. Electron microscopy further indicated that αS and Tau form complexes with filamentous subunits, whereas BSA forms larger amorphous complexes with αS and Tau. These large complexes may underlie the suppression of αS aggregation, while not substantially affecting the formation of co-assemblies. (Supplementary Fig. 30).

These results suggest that Tau can accelerate αS aggregation in a simplified two-protein system, but this effect is readily suppressed in a more complex environment containing additional proteins such as BSA. By contrast, the formation of αS–Tau co-assemblies appears relatively independent of αS aggregation and less influenced by additional proteins, pointing to potentially distinct biological roles for co-assemblies compared with general αS aggregates.

## Discussion

Although αS, Aβ, and Tau readily form co-assemblies in vitro, the extent to which these heterotypic structures exist in human-derived samples, particularly at the nanoscopic scale, remains poorly defined. Here, we adapted SiMPull to detect nanoscopic co-assemblies of αS, Aβ, and Tau in brain homogenate, soluble brain-derived protein fractions, serum, CSF, and saliva. Using this approach, together with immunodepletion, PK digestion, super-resolution imaging, and in vitro aggregation assays, we provide evidence that αS–Tau co-assemblies are readily detectable across multiple human sample types and represent a distinct subgroup of αS- and Tau-containing protein assemblies.

A central conclusion from this study is that αS–Tau co-assemblies should not be interpreted as a single molecular entity with a uniform biological role and biophysical feature. Rather, the data support a concept in which nanoscopic co-assemblies are heterogeneous populations whose properties vary according to biochemical fraction, tissue environment, antibody-accessible epitopes, and disease context. This distinction is important, as the presence of αS–Tau co-assemblies in both disease and control samples argues against a simple binary model in which their mere presence indicates pathogenicity. Instead, their widespread detection suggests that αS–Tau co-assembly may be a relatively common feature of the human proteostatic environment, while specific subpopulations, structural states, or biochemical contexts may be associated with disease, compensation, or downstream structural evolution.

In brain homogenates from the amygdala, αS–Aβ co-assemblies were below the detection limit, whereas αS–Tau co-assemblies represented a substantial proportion of αS-positive assemblies. Approximately 30–70% of αS aggregates in brain homogenates were associated with Tau, compared with approximately 10–30% in the soluble brain fraction and serum. This predominance of αS–Tau, rather than αS–Aβ, is consistent with the biology of these proteins: αS and Tau are both abundant in neurons and enriched in synaptic compartments, whereas Aβ primarily accumulates extracellularly. The high frequency of αS–Tau co-assemblies in brain extracts is also consistent with previous observations that larger αS–Tau co-aggregates can be present at levels comparable to pure αS aggregates in the Dementia with Lewy Bodies amygdala^9^.

Despite their abundance, αS–Tau co-assemblies displayed properties that were distinct from general αS or Tau aggregates. Super-resolution imaging and single-molecule intensity analysis showed that αS–Tau co-assemblies in brain homogenate had reduced antibody-accessible epitope exposure compared with general αS and Tau aggregates. General αS aggregates were of similar apparent size to αS–Tau co-assemblies but showed approximately 50% higher antibody binding. General Tau aggregates were larger (~30%) than αS–Tau co-assemblies and showed substantially higher antibody binding (up to 100-fold). These findings indicate that αS–Tau co-assemblies are not simply the additive combination of αS and Tau aggregates but instead appear to represent a structurally and/or compositionally distinct subset of protein assemblies.

PK digestion further emphasised this distinction. In brain homogenates, general αS aggregates contained a disease-associated PK-resistant subfraction, whereas αS–Tau co-assemblies did not show the same pattern. In the soluble brain-derived fraction, however, several assembly classes, including αS–Tau^HT7–LB509^, and Tau^HT7–HT7^, showed stronger PK resistance in controls than in PD samples. In addition, the intensity of αS-Tau^LB509–HT7^ was higher in controls than in PD even before PK digestion. These observations indicate that PK resistance is not an intrinsic or uniform property of αS–Tau co-assemblies. Instead, it depends on the assembly subclass and biochemical context. They also caution against assuming that PK resistance, disease association, and toxicity are interchangeable properties. Previous studies have reported that removal of PK-resistant αS does not necessarily improve neuronal survival in a PD mouse model^43^, and that sarkosyl-soluble fractions from human Lewy body dementia brain homogenates can induce CNS pathology in mice, where the induced deposits are not strongly PK-resistant^59^. The disease significance of αS–Tau co-assemblies is therefore unlikely to be resolved by assigning all detected co-assemblies to a single pathogenic or non-pathogenic category. The presence of large numbers of co-assemblies in control samples could indicate that some αS–Tau interactions are physiological, neutral, or compensatory. Conversely, disease-associated differences in intensity, epitope exposure, or protease sensitivity may reflect disease-induced structural evolution, altered clearance, changes in local protein concentration, or shifts in the relative abundance of assembly subpopulations with different biological effects. Although current datasets cannot distinguish between these possibilities, our findings do not claim a direct mechanistic role in disease. Instead, they define a measurable landscape of nanoscopic αS–Tau co-assemblies that differs significantly across biochemical and pathological contexts.

The in vitro aggregation experiments further support this context-dependent interpretation. Tau has often been proposed to contribute to synucleinopathies by promoting αS aggregation^60^. However, we found that BSA suppressed Tau-enhanced αS aggregation more profoundly than αS–Tau co-assembly formation, which experienced only relatively minor suppression. This result indicates that the relationship between Tau binding, αS aggregation kinetics, and co-assembly formation is not linear. αS–Tau interaction does not necessarily imply enhanced αS aggregation, and the outcome of this interaction can be strongly modified by the surrounding protein environment. This may help explain inconsistencies among previous experimental models, including reports that Tau knockout attenuates αS propagation in A53T transgenic mice, whereas reducing endogenous Tau does not affect αS pathology induced by intrastriatal injection of fibrillar αS^60,61^.

These findings also suggest that αS–Tau co-assemblies may occupy multiple positions along a biological spectrum. Some assemblies may be inert by-products of protein proximity, some may reflect compensatory sequestration, and others may represent structurally evolved species with altered propagation, clearance, or toxicity. Our observations in tauopathy samples are consistent with this idea: the intensity profile of αS–Tau co-assemblies distinguished AD from control amygdala homogenates, whereas the same metric did not clearly distinguish PD from controls. Thus, αS–Tau co-assembly formation may be influenced by disease-specific environments rather than being a universal marker of synucleinopathy.

From a more technical aspect, the main message of this work is, therefore, not that it identifies a single disease-driving mechanism, but that it establishes an experimentally controlled framework for detecting and comparing nanoscopic heterotypic assemblies directly in human-derived samples. This is an important technical and conceptual gap. Most studies of protein co-aggregation have focused either on in vitro systems or on larger pathological inclusions, whereas small soluble or partially soluble co-assemblies remain difficult to detect, quantify, and compare. By combining SiMPull with immunodepletion, PK digestion, super-resolution imaging, biofluid analysis, and controlled in vitro perturbation, we show that these assemblies can be measured reproducibly and that their properties are not reducible to those of general αS or Tau aggregates.

Several limitations remain. SiMPull can identify species containing two antibody-recognised components, but it cannot fully resolve assemblies containing multiple additional proteins, post-translational modifications, lipid components, nucleic acids, or other cofactors. Epitope-specific antibody detection may also capture only subsets of assemblies, particularly because αS and Tau can undergo truncation, conformational masking, and disease-associated structural rearrangement. For example, truncated forms of αS lacking the C-terminus have been reported to have distinct pathological properties^62,63^. As aggregation proceeds, epitope exposure is unlikely to remain uniform, making antibody accessibility both a useful structural readout and a limitation. In addition, fluorescence-based detection provides high molecular specificity but cannot provide the atomic or ultrastructural resolution available through electron microscopy, atomic force microscopy, cryo-EM, or mass-spectrometry-based compositional analysis.

Future studies will therefore need to isolate and resolve specific co-assembly subpopulations rather than treating αS–Tau co-assemblies as a single class. Solvent-based enrichment of protein assemblies, combined with immunoprecipitation, proximity-based labelling, crosslinking mass spectrometry, cryo-EM, or AFM, may help define the molecular composition and structure of these species. Comparative analyses across brain regions, disease stages, cellular compartments, and biochemical fractions will also be important, because our findings indicate that co-assembly properties are strongly context-dependent. In parallel, computational approaches for predicting protein–protein interactions and modelling intrinsically disordered protein assemblies may help prioritize candidate interaction interfaces for experimental validation^64–68^.

Overall, our work identifies nanoscopic αS–Tau co-assemblies as previously underexplored human-derived protein assemblies with heterogeneous structural and biochemical properties. The data do not support assigning these co-assemblies a single uniform role in disease. Instead, they indicate that αS–Tau co-assemblies comprise multiple context-dependent populations whose biological consequences may differ across brain region, biochemical fraction, and disease state. By establishing a framework to detect and characterize these species in human samples, this study provides a foundation for future work aimed at determining which, if any, co-assembly subpopulations are toxic, protective, compensatory, or secondary consequences of disease-associated protein remodelling.

## Methods

### Ethical statement

This research complies with all relevant ethical regulations. Multiple ethical approvals were obtained for this study: (1) Soluble fraction of brain extracts - reference 18/LO/0721, Queen Square Brain Bank for Neurological Disorders, University College London. (2) Brain homogenates - reference 16/LO/0508, Cambridge Brain Bank, with approval from the London–Bloomsbury Research Ethics Committee. (3) Saliva samples – Reference 03/303, NRES Committee East of England—Central Cambridge. (4) Serum and CSF – reference 16/EE/0445, East of England–Essex Research Ethics Committee. All samples were voluntarily donated without compensation, and written informed consent was obtained. Biological sex of participants was determined by self-report. Controls were selected to be age- and sex-matched at the group level to participants with disease.

### Sample collection and preparation

1. Aggregation of αS: Monomeric wild-type αS was obtained from *Escherichia coli* (*E. Coli*) according to an existing protocol^69^. Monomers at a concentration of 70 μM were supplemented with 0.01% NaN₃ in PBS and incubated for 96 hours under constant shaking at 200 rpm at 37 °C. Sonicated αS aggregates were generated using pulse sonication (QSonica, Q125, 1/8-inch tip). Each cycle consisted of a 5-s on phase followed by a 15-s off phase at 40% power, with a total of three cycles performed.
2. Aggregation of Aβ: Monomeric Aβ_1-42_ was purchased commercially (Bio Trend, AS-24235) and purified according to the supplier’s protocol. Monomers at a concentration of 20 μM were supplemented with 0.01% NaN₃ in 20 mM sodium phosphate (NaP), 10 mM NaCl, and 180 μM EDTA, then incubated for 48 hours at 37 °C^70^. Sonicated Aβ aggregates were generated using pulse sonication (QSonica, Q125, 1/8-inch tip). Each cycle consisted of a 5-s on phase followed by a 15-s off phase at 40% power, with a total of three cycles performed.
3. Aggregation of Tau: The item is commercially obtained. (Stressmarq, Human Recombinant Tau-441 (2N4R) Wild-Type Oligomers, Catalog No. SPR-497). See ThT fluorescence assay section for co-assemblies used in Fig. 9.
4. Recombinant Tau-αS-Aβ co-aggregates: 5 μM αS aggregates, 1 μM Tau aggregates and 10 μM Aβ aggregates were mixed and co-aggregated at 37 °C for 48 h. The heat treatment was performed with a heating block.
5. Recombinant αS-Aβ co-aggregates: 10 μM αS aggregates and 10 μM Aβ aggregates were mixed at a 1:1 ratio and incubated for 8 hours under constant shaking at 200 rpm at 37 °C.
6. Human serum: Serum, saliva and CSF samples were collected from people with idiopathic PD and similarly aged control participants (without neurological disease) attending the Cambridge Parkinson’s Research Clinic. All participants provided informed consent, and ethical approval was granted by the East of England-Essex Research Ethics Committee (16/EE/0445) and the East of England – Central Cambridge Research Ethics Committee (03/303). Blood was drawn via venepuncture using S-Monovette tubes (7.5 mL). Samples were left to clot for 15 min at room temperature, then centrifuged at 2200 × *g* for 15 min. The supernatant (serum) was collected and stored at −80 °C. See Supplementary Tables 3 and 4 for details of patient information.
7. Human CSF: CSF was obtained via lumbar puncture at the L3/4 interspace using a sterile technique with 1% lidocaine as a local anesthetic. Samples were centrifuged at 300 × *g* for 10 min at 4 °C, and the supernatant was stored at −80 °C. See Supplementary Table 5 for details of patient information.
8. Human saliva: Participants fasted for at least one hour before collection. Exclusion criteria included dental or oral disease, smoking within four hours, or alcohol consumption within 12 hours before sampling. Samples were collected between 10:00 AM and 11:30 AM using the passive drool method, with participants allowing saliva to pool in their mouths before drooling into a sterile container without stimulation. Samples were processed on ice, and protease inhibitors (Sigma P, 10 μL/mL of saliva) and phosphatase inhibitors (sodium orthovanadate, 3 μL/mL of saliva) were added. Samples were centrifuged at 2600 × *g* for 15 min at 4 °C, followed by a second centrifugation at 15,000 × *g* for 15 min at 4 °C. The final supernatant was aliquoted (100 μL) into cryotubes and stored at −80 °C. Upon receipt, 100 μL aliquots were thawed, subdivided into 11 μL portions, and stored at −80 °C without additional freeze-thaw cycles. See Supplementary Table 6 for details of patient information.
9. Post-mortem brain extracts: For PD and Tauopathy homogenates, post-mortem brain tissues were obtained from the Cambridge Brain Bank with approval from the London–Bloomsbury Research Ethics Committee (16/LO/0508, Supplementary Tables 7 and 9). All brain samples were voluntarily donated without compensation. Brains were flash-frozen and stored at −80 °C. Fresh-frozen brain tissue was homogenised at 4 °C using a VelociRuptor V2 Microtube Homogeniser (Scientific Laboratory Supplies, Cat. No. SLS1401) in 10 volumes of homogenization buffer (10 mM Tris-HCl, 0.8 M NaCl, 1 mM EGTA, 0.1% Sarkosyl, 10% sucrose; pH 7.4) supplemented with complete™ Protease Inhibitor and PhosStop™ Phosphatase Inhibitor. The homogenate was centrifuged at 21,000 × *g* for 20 min at 4 °C, and the upper 90% of the supernatant was collected. The remaining pellet was resuspended in an additional five volumes of homogenization buffer, homogenized again, and centrifuged under the same conditions. The upper 90% of this second supernatant was pooled with the first, aliquoted, and stored at −80 °C for subsequent experiments. For single-molecule experiments, brain homogenates were diluted 1:3 in ELISA diluent (ab193972).

For preparation of the soluble brain fraction, samples were collected under the protocol RA032611/1 (REC reference 18/LO/0721, Queen Square Brain Bank for Neurological Disorders, University College London). Frozen brain pellets were manually homogenised in 1× PBS supplemented with protease and phosphatase inhibitors (Sigma; Thermo Fisher Scientific). The tissue suspensions were lysed by sonication (either Sonic Dismembrator Model 300, setting 40 for 15 s at 4 °C, or Fisher Scientific Model 705, amplitude 5% for 15 s at 4 °C). Homogenates were ultracentrifuged at 100,000 × *g* for 1 h at 4 °C, and the resulting supernatant was collected as the soluble protein fraction^71^. Sample details are summarised in the Supplementary Table 8.

### Preparation of SiMPull coverslips

Coverslips were prepared following our previous protocol with minor modifications. Briefly, glass coverslips (26 × 76 mm, thickness #1.5, VWR, Cat. MENZBC026076AC40) were sequentially cleaned in a bath sonicator using MQ water (10 min), acetone (10 min), and methanol (10 min). After cleaning, a 50-well polydimethylsiloxane (PDMS) chamber gasket (GRACE Bio-Labs, Cat. 103350) was affixed to the coverslip. Each well was then filled with 6 µl of a 1:1 mixture of Rain-X (Rain-X Rain Repellent) and isopropanol, which was allowed to dry naturally. The Rain-X–isopropanol mixture was filtered (Millex, SLGV004SL) before use. Prepared coverslips were stored at room temperature and used within four weeks.

### Preparation of antibodies

The details of antibodies used in this work can be found in SI. Biotinylation and dye-labelling were performed on some antibodies. Briefly, we used a commercial kit for dye labelling (Invitrogen, Z11235) and biotinylation (Thermo, 90407). The processing was completed according to the manufacturer’s protocol with 2 additional purification steps. Plain antibodies were purified using Amicon filters with 3 buffer exchange cycles before processing. The subsequent products were then purified using the same method.

### Protocol of SiMPull assay

Each well was rinsed twice with PBS before applying 10 µl of NeutrAvidin (0.1 mg/ml, Thermo Scientific, 31000) diluted in PBS. The solution was incubated for 15 min, then removed, followed by three washing steps with PBS. Washing was performed by pipetting buffer in and out of the imaging wells. Passivation was carried out by adding 10 µl of F-127 (Invitrogen, P6866, 1% diluted in PBS, filtered 0.22 µm) and incubating for 60 min. The solution was then removed, followed by three additional washing steps with PBS-T (0.05% Tween 20, Fisher BioReagents™ in 1X PBS, Gibco™). Next, 10 µl of capture antibody was added to each well and incubated for 15 min, followed by removal and another three washing steps with PBS-T. Samples (10 µl) were then added to each well, with dilution and incubation times specified in Supplementary Table 2 (see SI). After sample incubation, 10 µl of detection antibody was added to each well and incubated for 15 min, followed by removal and two final washing steps with PBS-T. The imaging well was then filled with 10 µl of PBS to prevent drying during image acquisition. See SI for the details and working conditions of antibodies used in the work.

For the experiments shown in Figs. 5, 6, 8, and 9, along with the associated supporting data, an improved protocol was applied. Briefly, a mixture of 1% F-127 (spiked with biotinylated F-127 at a 1:50 ratio) was incubated on Rain-X–coated surfaces, followed by three washes with PBST. Next, 10 µl of NeutrAvidin (0.1 mg/ml, Thermo Scientific, 31000) diluted in PBST was applied to the surface. All subsequent steps were carried out identically to the original protocol.

### Proteinase K digestion experiments

Thermolabile Proteinase K (New England Biolabs, P8111S) was used for all PK digestion experiments. Briefly, PK was added to undiluted samples at a 1:100 ratio and incubated for 30 min at room temperature. Digestion was terminated by adding Halt™ Protease Inhibitor Cocktail (100X) at a 1:100 ratio, followed by a 20-min incubation before further dilution with sample diluent.

### Immunodepletion experiments

Two types of streptavidin-coated magnetic beads (Dynabeads MyOne Streptavidin C1 and T1, mixed at a 1:1 ratio) were washed three times with PBST before incubation with the relevant biotinylated antibodies. A total of 20 μg of antibody was incubated with 100 μL of beads for 30 min, followed by three additional PBST washes. The antibody-coupled beads were then diluted into the sample diluent (matched to the corresponding sample buffer) at a 1:10 ratio. This bead-containing diluent was mixed with samples at the desired dilution factor for 60 min, and beads were subsequently isolated using a magnetic rack.

### ThT fluorescence assay

Under the conditions specified in the main text, samples were diluted in running buffer and loaded into 96-well half-area black polystyrene plates with a clear bottom and polyethylene glycol coating (Corning). Each 15 mL of buffer contained 15 µL of 1 M HEPES, 30 µL of 0.5 M EDTA, 150 mg of NaCl, 1.13 µL of 10% SDS, and 14.95 mL of Milli-Q water. Aggregation assays were performed at 37 °C under quiescent conditions using a CLARIOstar plate reader (BMG Labtech). ThT fluorescence was monitored through the bottom of the plate every minute using a 440 nm excitation filter and 480 nm emission filter.

### Optical setup for diffraction-limited and super-resolution imaging

Both diffraction-limited and super-resolution imaging were performed using the same optical setup. Briefly, 488 nm (iBeam-SMART, Toptica), 561 nm (Cobalt Jive, Cobalt) and 638 nm (Cobolt MLD 638, Cobolt) lasers were coupled into a TIRF objective (UPLSAPO, 100×, TIRF, Olympus) mounted on an inverted Ti-E Eclipse microscope (Nikon, Japan) via a multimode optical fibre. A built-in Perfect Focus system (PFS) was used to maintain the focal plane during imaging. Fluorescent emission was collected by the same objective, separated from the excitation laser using a dichroic mirror (Di01-R405/488/561/635, Semrock), and directed to an EMCCD camera (Evolve 512, Photometrics) through a 1.5× beam expander. Emission filters (LP02-568RS25, Semrock, and FF01-587/35–25, Semrock) were used to refine the signal. Each pixel on the camera chip corresponded to 102.3 nm. Data acquisition was carried out using MicroManager.

For diffraction-limited imaging, 9–16 fields of view (FoV) were acquired per imaging well, with 50 frames captured at an exposure time of 50 ms per frame. Each FoV covered an area of approximately 2500 µm².

For dSTORM imaging, an appropriate imaging buffer was applied. Two different buffers were used: a commercial buffer (Everspark, Idylle) for experiments in Fig. 2, and a custom buffer (50 mM PBS-Tris, 0.5 mM glucose, 1.3 μM glucose oxidase, 2.2 μM catalase, and 50 mM MEA) for the remaining dSTORM experiments. To prevent oxygen penetration, imaging wells were carefully sealed with nail polish. For each imaging well, four FoV were acquired, with 5000–8000 frames captured at an exposure time of 30 ms per frame.

### Electron microscopy

Samples were washed twice with Milli-Q water (2 × 10 μL) and negatively stained with 2% (w/v) uranyl acetate (10 μL, 2 min), followed by two additional Milli-Q water washes (2 × 10 μL). TEM grids were glow-discharged using a Quorum Technologies GloQube system at 25 mA for 60 s. Imaging was performed on a Thermo Scientific (FEI, Hillsboro, OR) Talos F200X G2 transmission electron microscope operated at 200 kV. Images were recorded with a Ceta 4k × 4k CMOS camera.

### Data analysis

Diffraction-limited single-molecule counting was performed using a single-molecule localization engine in ThunderSTORM^72^. The images were processed using a wavelet filter, and the localizations were identified using a hybrid threshold of 0.5 × std(Wave.F1) + 0.1 × mean(Med. F). This arrangement gives a higher resistance to noise by combining the adaptive threshold and the mean-value threshold. The statistics were performed using GraphPad Prism 10.0. The principles of statistical tests follow our previous reports. Briefly, samples with a small cohort size (<9) were examined with the non-parametric two-sided Mann-Whitney test. Larger data sets needed to pass the normality test (Kolmogorov-Smirnov test) for the parametric *t*-test, otherwise, a non-parametric two-sided Mann-Whitney test or ANOVA would be performed. The colocalization data were analysed with ComDet. (https://github.com/UU-cellbiology/ComDet) For the data in Supplementary Fig. 26, the relevant analysis was performed with Python, since GraphPad Prism does not support ART-ANOVA.

dSTORM data were analysed using published ImageJ plug-ins, with an automation code integrating these independent libraries. The drift correction, image reconstruction and morphology analysis were performed by a mean shift algorithm^73^, ThunderSTORM^72^ and morphology library^74^, respectively. To avoid blurred images caused by microscope focusing stabilization, the first 200 frames were excluded from further analysis. Localizations were identified using ThunderSTORM, and drift correction was applied using the mean shift algorithm. The drift-corrected localizations were then filtered with a localization merge step, followed by a density filter to remove false-positive signals. These processed localizations were used to reconstruct the super-resolved images. Morphological analysis was conducted to obtain area and circularity measurements. The maximum Feret diameter was used to represent the size of super-resolved aggregates.

### Reporting summary

Further information on research design is available in the Nature Portfolio Reporting Summary linked to this article.

## Supplementary information

SUPPLEMENTARY INFO (PDF version)

Reporting Summary

Transparent Peer Review file

## Source data

Source data file

## Data Availability

All data necessary to evaluate the claims in this paper are present in the main text or the supplementary materials. Source data are provided with this paper.

## Code availability

The relevant codes for analysis are available from https://github.com/YPZ858/DF-single-molecule-couting- (single-molecule counting) and https://github.com/YPZ858/Super-res-code/issues (super-resolution imaging). The permanent version of all code is deposited on Zenodo: https://doi.org/10.5281/zenodo.21904517^75^and https://doi.org/10.5281/zenodo.21904440 [https://doi.org/10.5281/zenodo.21904440]^76^.
