# Supplementary material for: Imaging nanoscopic co-assemblies of α-synuclein and Tau in human samples
Source: Nat Commun. 2026 Sep 18;17:10951. doi: 10.1038/s41467-026-77728-1 (PMC13680374; doi:10.1038/s41467-026-77728-1)
Supplement: Supplementary file 1 [file 41467_2026_77728_MOESM1_ESM.pdf]

Imaging nanoscopic co-assemblies of α-synuclein and Tau in human samples

Yu P. Zhang[a,b,*], Amber Wallis[c], Clara Obensa Bertroli[c], Cherida Zhang[a,b], Melanie Burke[a,b], Florence Layburn[a,b], Evgeniia Lobanova[a,b], Martin Furlepa[a,d,e], Shekhar Kedia[a,b], Yuhao Cui[a,b], Prasanna Suresh[a,b], Woo Suk Yang[a,b,f], Annelies Quaegebeur[d], Tim Bartels[c], Caroline H. Williams-Gray[d] and David Klenerman[a,b,*]

[a] Department of Chemistry, University of Cambridge, Lensfield Road, Cambridge CB2 1EW, United Kingdom

[b] UK Dementia Research Institute at Cambridge, Cambridge CB2 0XY, United Kingdom

[c] UK Dementia Research Institute at University College London, London, W1T 7NF, United Kingdom

[d] Department of Clinical Neurosciences, University of Cambridge, Cambridge CB2 0PY, United Kingdom

[e] Manchester Centre for Clinical Neurosciences, Salford Royal NHS Foundation Trust, Manchester Academic Health Science Centre, Manchester, M6 8HD, United Kingdom

[f] St. Thomas' Hospital London, London SE1 7EH, United Kingdom

*Corresponding authors: Yu P. Zhang (yz566@cam.ac.uk) and David Klenerman (dk10012@cam.ac.uk)

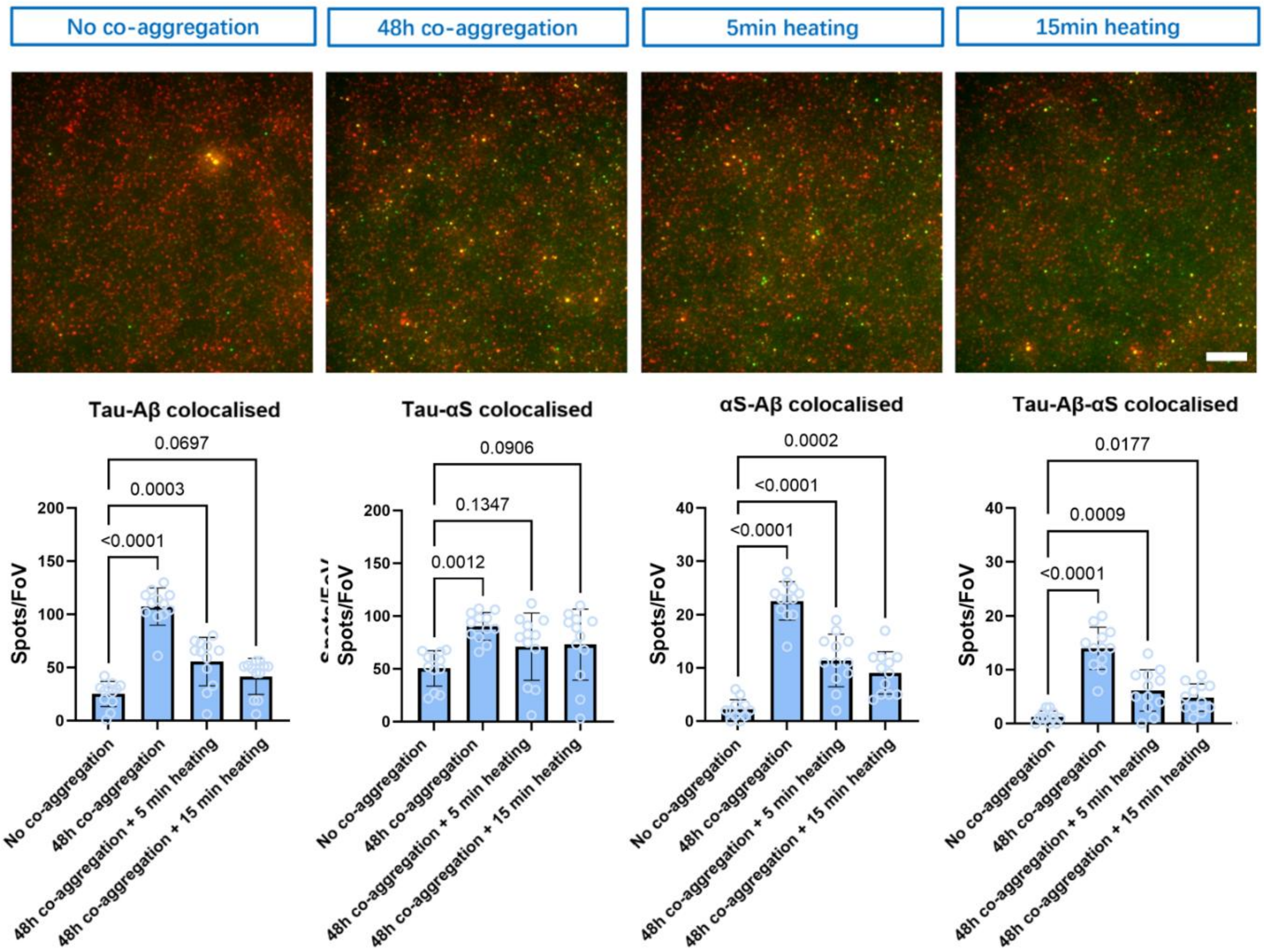

Supplementary Figure 1: Quantification of colocalised signals from single-molecule imaging of Tau–αS–Aβ co-assemblies. The number of colocalised molecules significantly increased upon co-aggregation, consistent with the results shown in Fig. 1. Scale bar: 5 µm. (Data are expressed as mean ± SD. n = 12 from 12 FoVs). Ordinary One-way ANOVA with post-hoc multiple-comparison correction was used for statistical test. Source data are provided as a Source Data file.

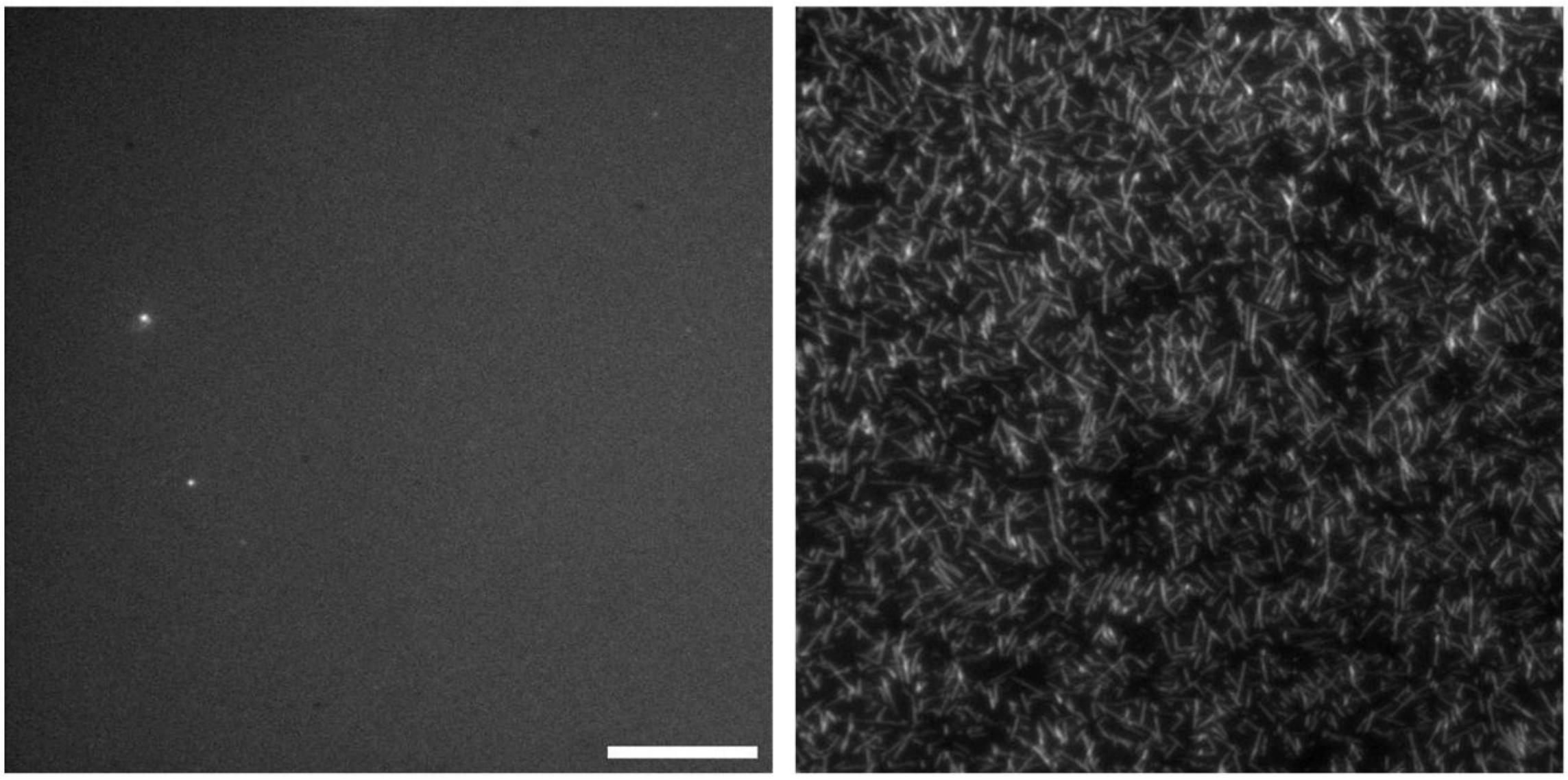

Supplementary Figure 2: Amytracker 520 imaging of co-assemblies. The unheated sample incubated for 48 h under co-aggregation condition (left) showed minimal Amytracker 520 signal, indicating that the majority of the co-assemblies lack β-sheet–rich structures. In contrast, the mature αS fibrils (right) showed strong signal with Amytracker staining. Scale bar: 10 µm.

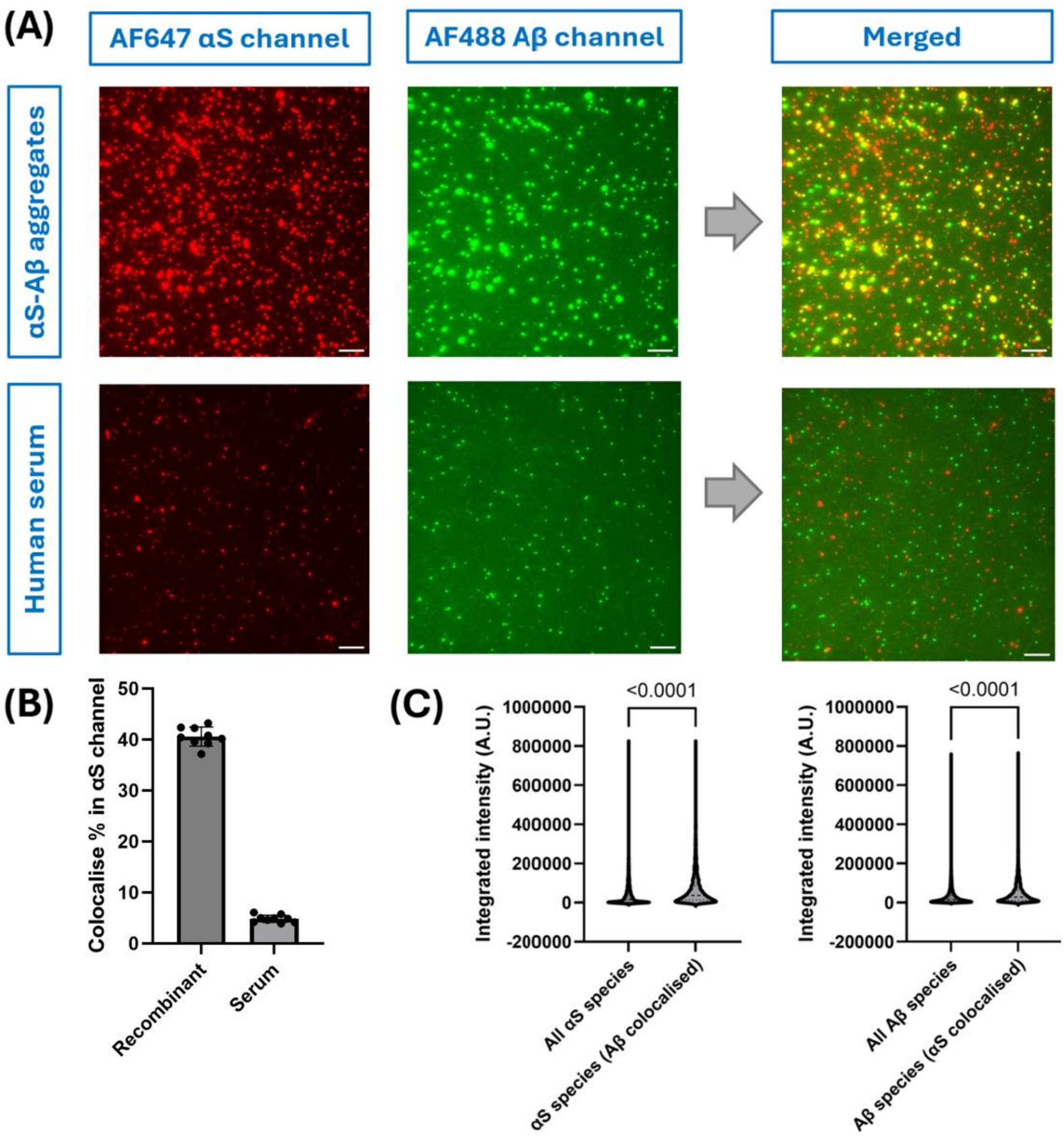

Supplementary Figure 3: Quantification of dual-colour single-molecule images of αS-Aβ co-assemblies in recombinant and serum samples with representative images. (A). Representative images of dual-colour single-molecule images of αS-Aβ co-assemblies in recombinant and serum samples. In both conditions, Syn211 antibodies were used as capture. The Red (AF647) signal was from the Syn211 detection antibody and the green(AF488) signal was from the 6E10 antibody. The co-localised signal was observed in recombinant samples but not serum. The molecules with co-localised signals tend to be the larger species. Scale bar: 5µm. **(B).** The colocalisation rate of αS-Aβ in recombinant co-assemblies and human serum. (Data are expressed as mean ± SD. n = 9 from 9 FoVs) **(C).** Intensity comparison between all detected αS/Aβ-positive molecules and colocalised molecules, showing higher intensity in the latter. (n=8654 for all αS species, n=4996 for αS species colocalised with Aβ, n=11669 individual aggregates, for all Aβ species, n=4996 individual aggregates for Aβ species colocalised with αS). Source data are provided as a Source Data file.

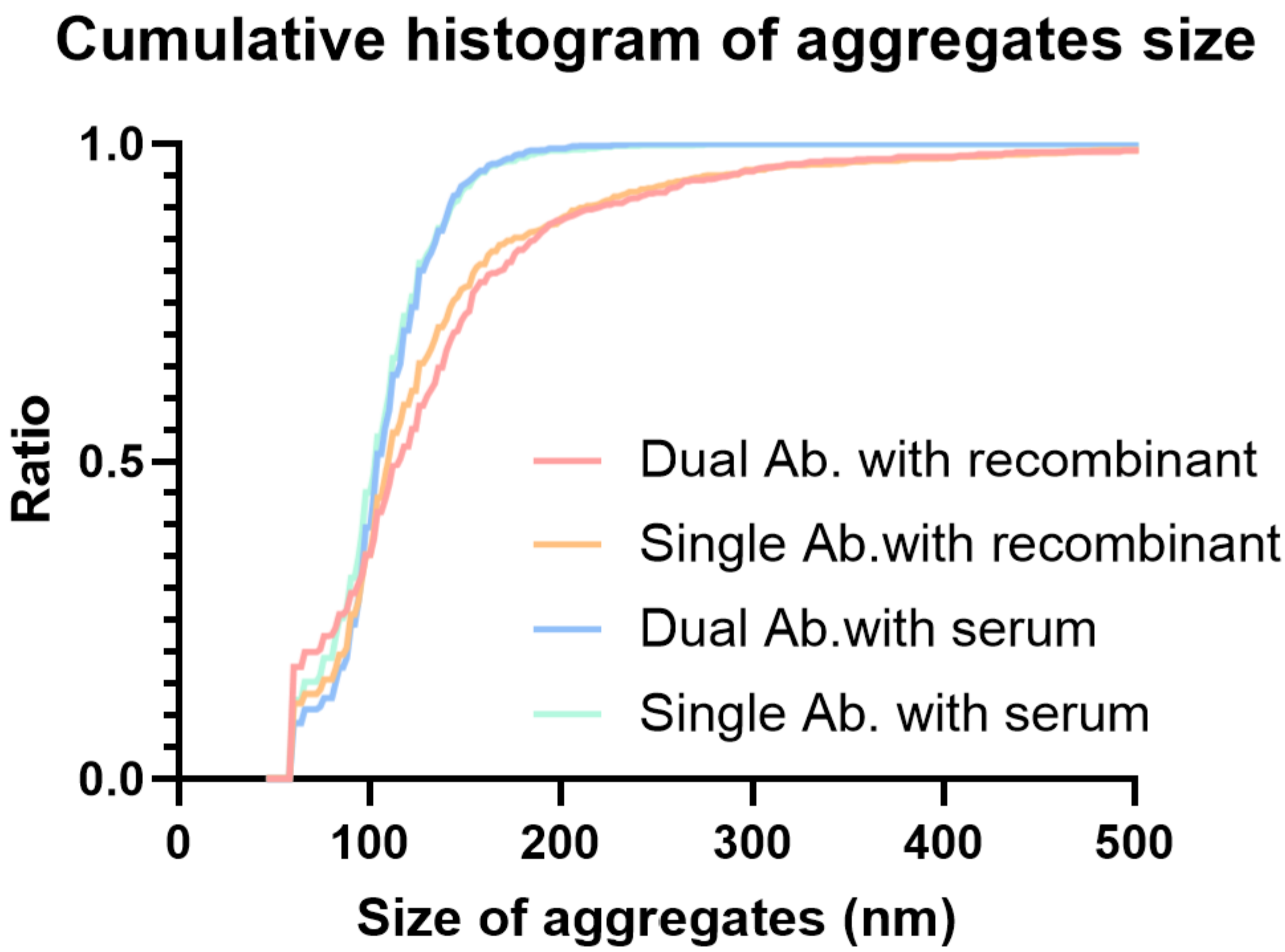

Supplementary Figure 4: Cumulative histogram of aggregates size, derived from data shown in Fig. 2 E. There is no distribution differences between two imaging conditions with recombinant samples. A distribution difference was observed with serum samples, where dual-antibody imaging condition yields a higher portion of larger aggregates. Source data are provided as a Source Data file.

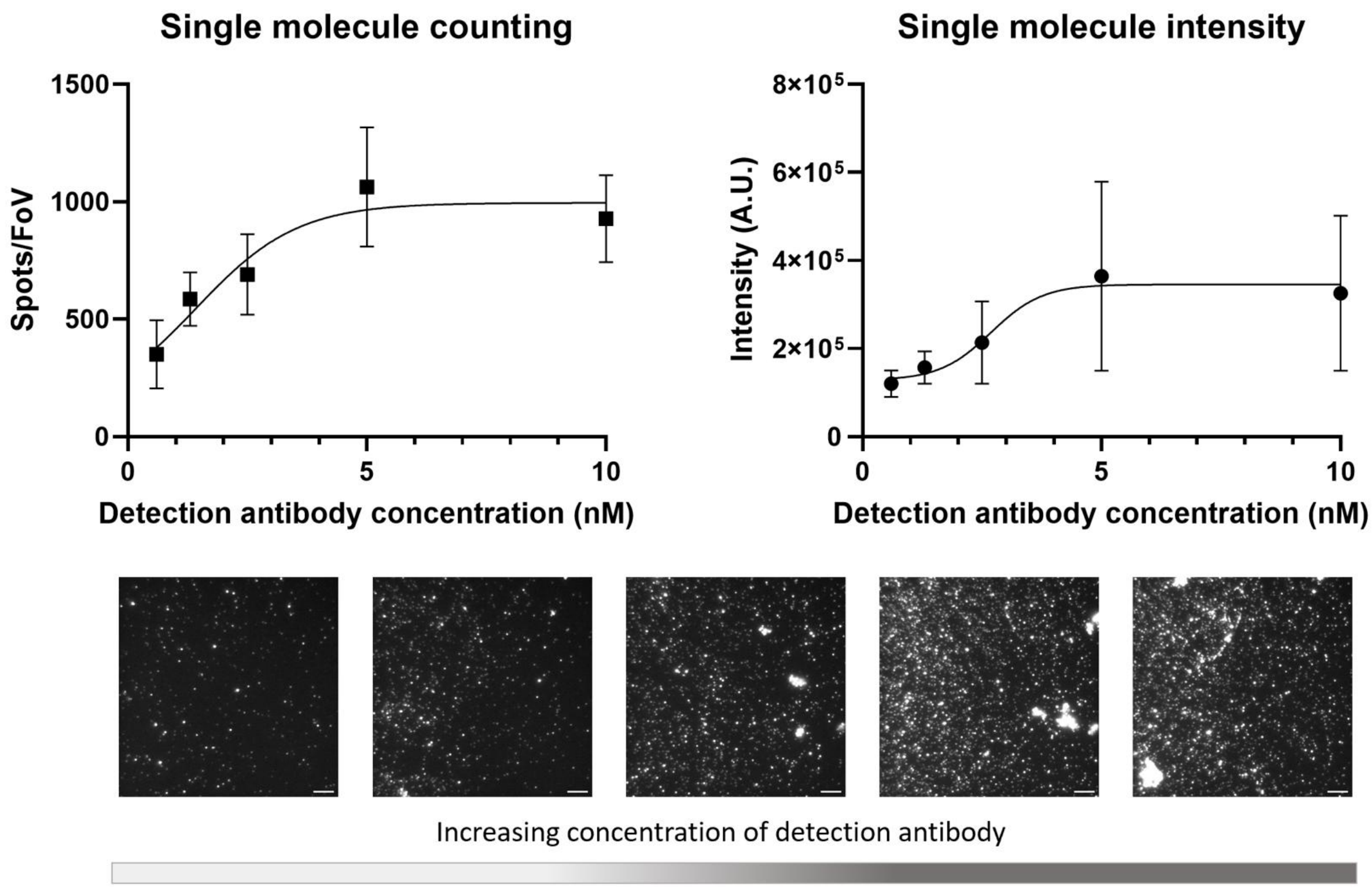

Supplementary Figure 5: The fluorescent signal dependence of detection antibody concentration in serum. The increasing antibody concentration can improve the signal level until saturation. Upper panel is quantified single-molecule counting from different conditions. Lower panel is quantified intensity from different conditions. Syn-211 antibody was used as both capture and detection, similar to Fig. 2. However, the pooled serum sample here is different from the one used in the Fig. 2. (Data are expressed as mean ± SD. n=12 from 12 FoVs taken from each sample) Fitting parameter: Sigmoidal, 4PL, X is log(concentration). Scale bar: 5µm. Source data are provided as a Source Data file.

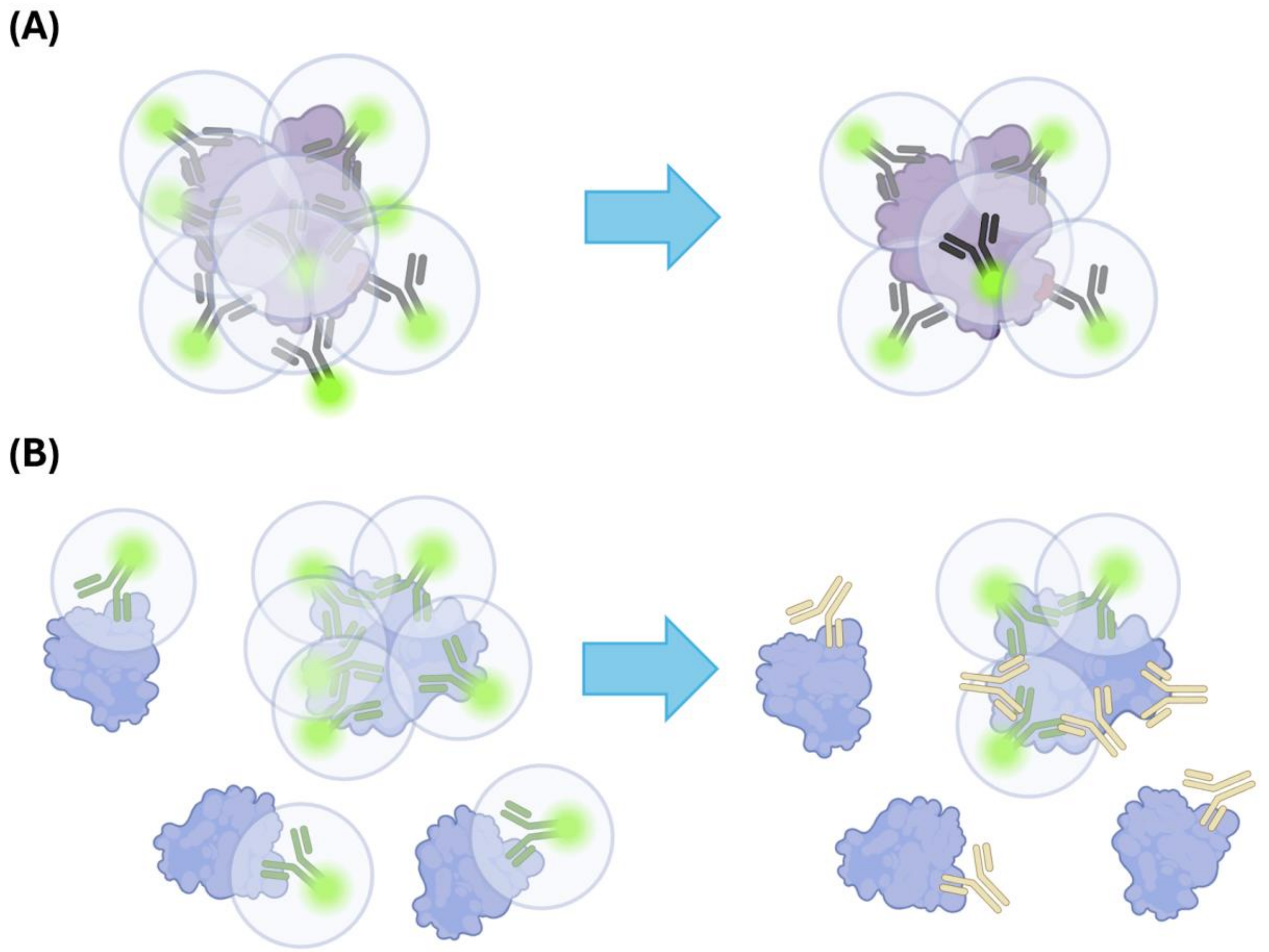

Supplementary Figure 6: Effects of dual-antibody labeling on aggregate intensity and size. (A). For recombinant aggregates with abundant binding sites, dual-antibody labelling results in reduced fluorescence intensity per molecule without affecting the measured aggregate size. **(B).** For smaller aggregates with limited binding sites, competition between αS and Aβ antibodies leads to reduced labelling by the primary detection antibody. This results in decreased fluorescence intensity but an apparent increase in the average size distribution, as only the relatively larger, antibody-rich aggregates remain detectable. The figure created via BioRender. Zhang, Y. (2026) https://BioRender.com/e63sj4f.

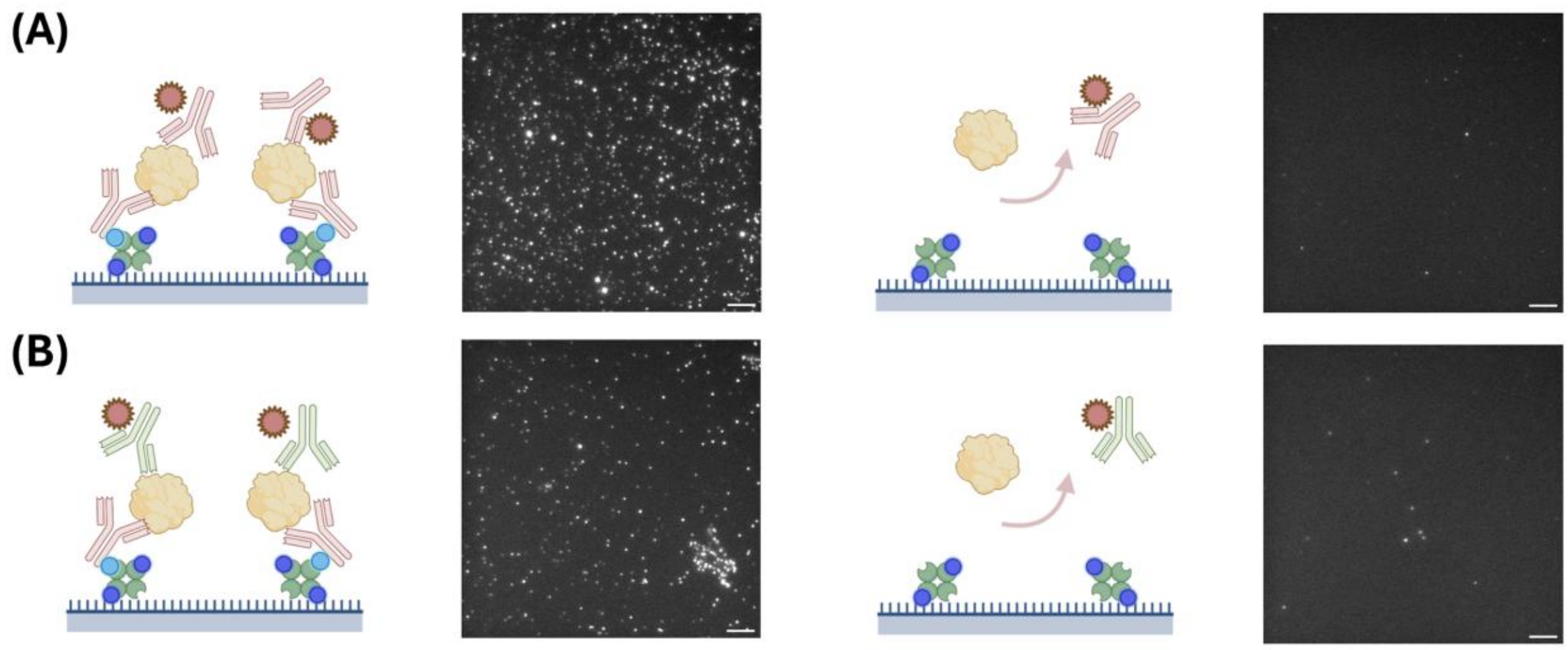

Supplementary Figure 7: Schematic of control capture experiment. Briefly, when no capture antibody is present on the surface, a low signal should be expected, reflecting the fact that signals arise from the capture antibody instead of the non-specific sample-surface interaction. **(A).** Representative image of αS aggregates in serum. Syn211 antibody was used as both capture and detection. **(B).** Representative image of αS-Aβ co-assemblies in serum. Syn211 antibody was used as capture. 6E10 antibody was used for the detection. bar: 5µm. The figure created via BioRender. Zhang, Y. (2026) https://BioRender.com/e63sj4f.

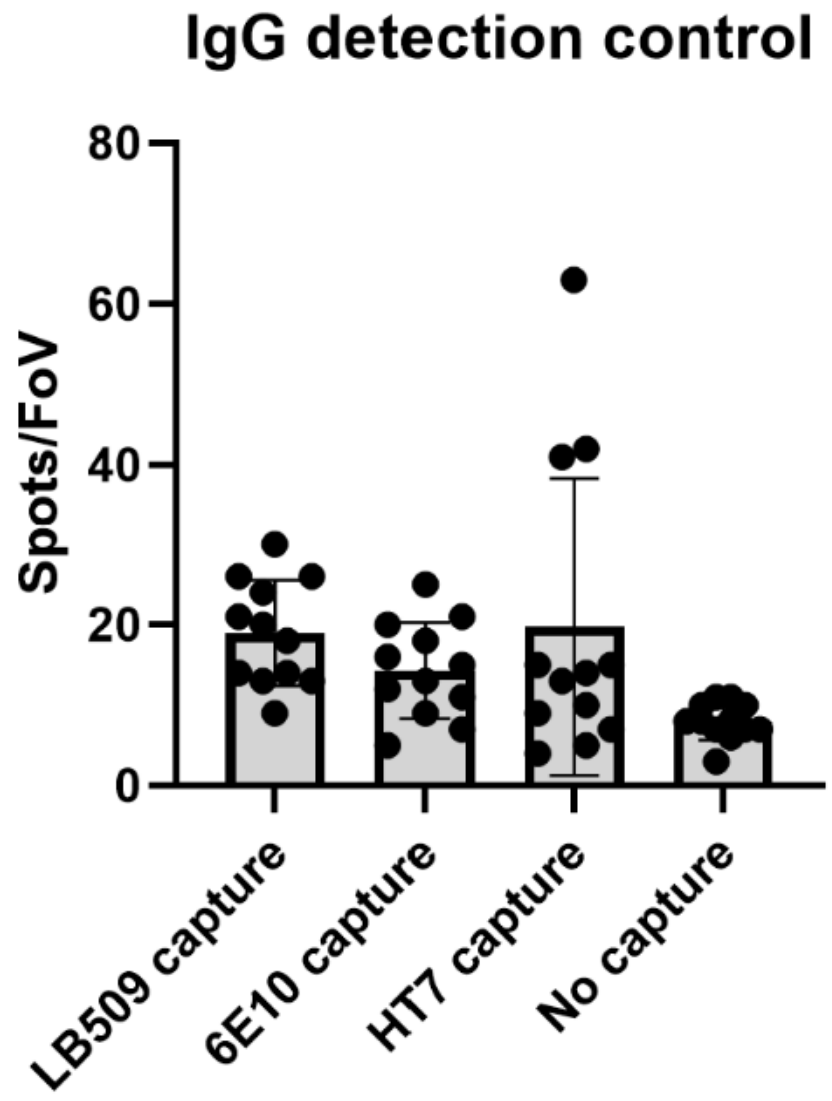

Supplementary Figure 8: Single-molecule counting of IgG technical controls. Briefly, the pooled brain samples were applied to wells coated with different capture antibodies. An IgG isotype control (AF647) was used for detection. A low level of signal was observed, indicating that the non-specific antibody-samples interaction is not significant. (Data are expressed as mean ± SD, n=12 from 12 FoVs) Source data are provided as a Source Data file.

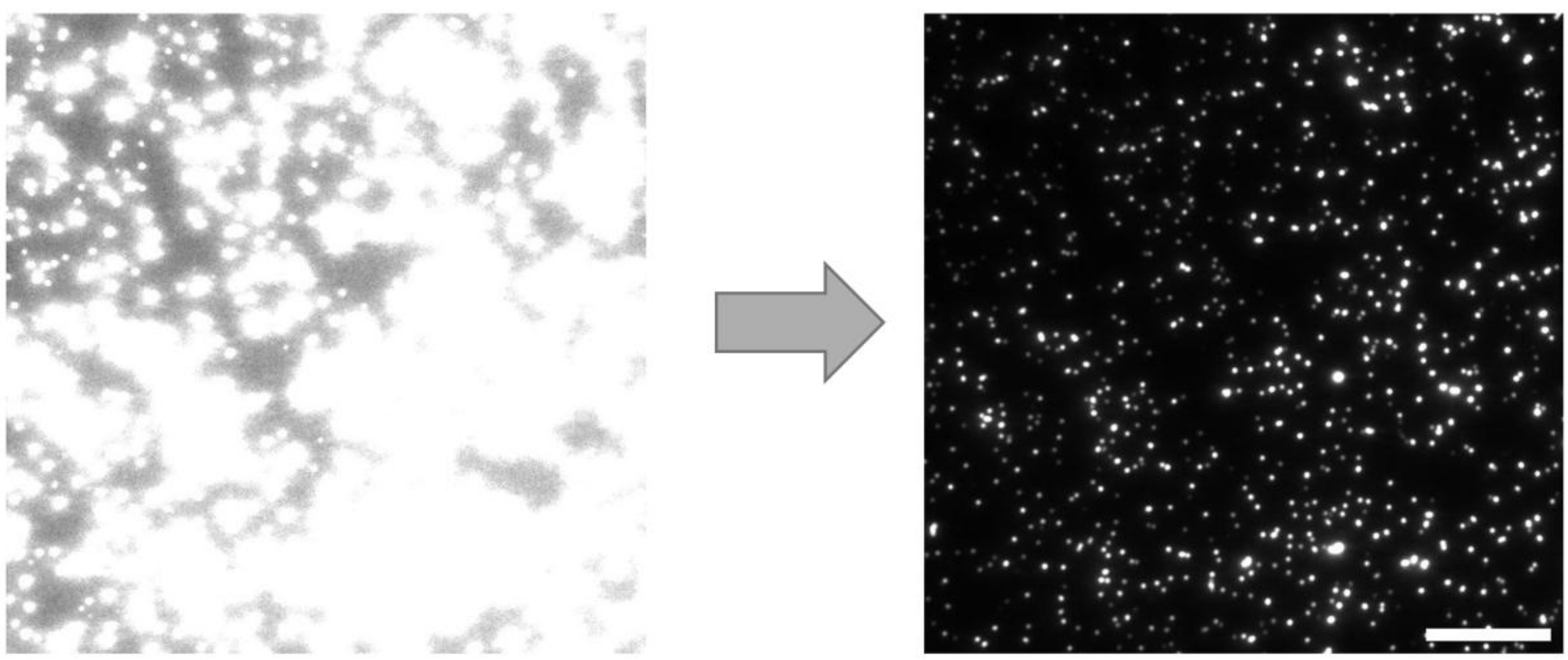

Supplementary Figure 9: Tau aggregates with adjusted contrast. HT7 antibody was used for both capture and detection. These species are much brighter than co-assemblies and look over-exposure when identical contrast was applied. The adjusted figure better presents the single molecules. Scale bar: 10μm.

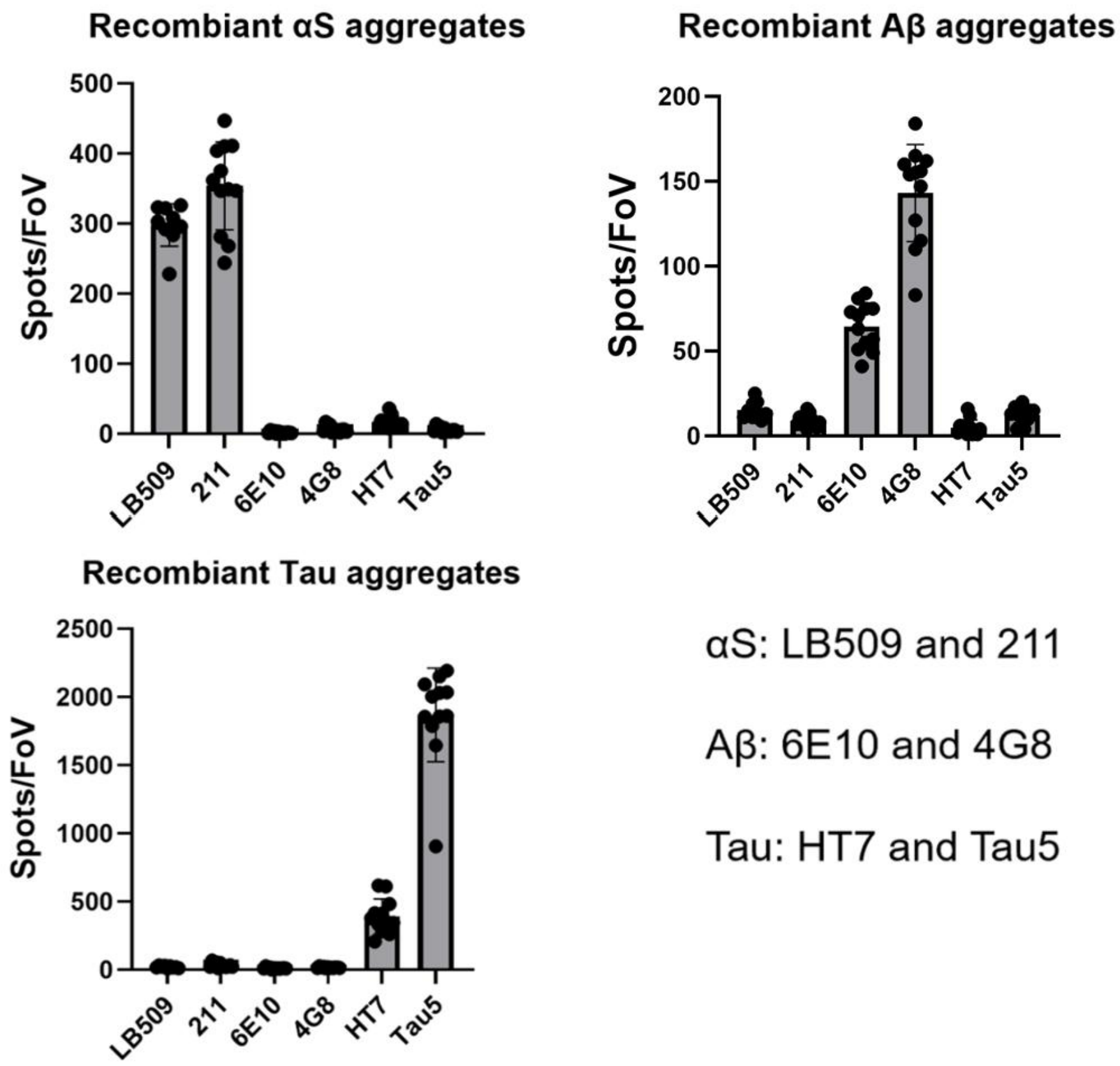

Supplementary Figure 10: Antibody cross-talk measurement with recombinant protein aggregates. Briefly, 3 different aggregates (αS, Aβ, Tau) were immobilised on the surface with Syn211, 6E10 and HT7 antibodies, respectively. 6 different detection antibodies were used as the detection to assess the cross-talk. Only correct antibodies tend to generate signals. (Data are expressed as mean ± SD. n=9, from 9 Fov for sets with LB509 detection. n = 12, 12 FoV for the other sets). Source data are provided as a Source Data file.

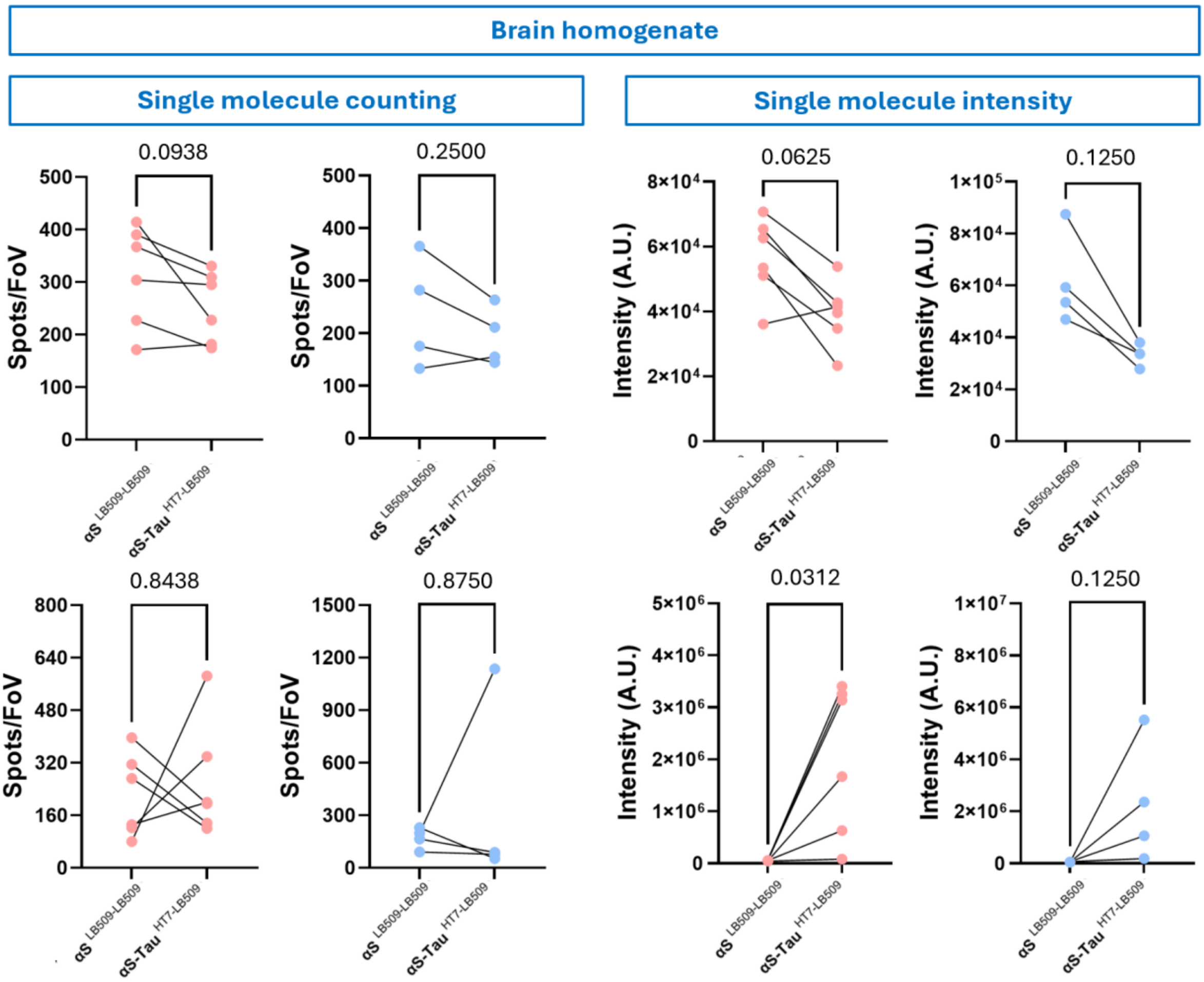

Supplementary Figure 11: Paired comparison between αS and Tau species present in PD and control group. In the PD group, 5 out of 6 samples show more $\alpha S^{LB509\text{-}LB509}$ counts than $\alpha S\text{-}Tau^{HT7\text{-}LB509}$. In the control group, 3 out of 4 samples demonstrate the same pattern. No clear pattern was observed between $\alpha S\text{-}Tau^{LB509\text{-}HT7}$ and $Tau^{HT7\text{-}HT7}$. (B) Paired comparisons of single-molecule intensity between αS-Tau co-assemblies and general species of αS and Tau, with separate analysis for PD and control groups. In the PD group, 5 out of 6 samples show higher $\alpha S^{LB509\text{-}LB509}$ intensity than $\alpha S\text{-}Tau^{HT7\text{-}LB509}$. In the control group, 4 out of 4 samples demonstrate the same pattern. Meanwhile, all samples have a higher $Tau^{HT7\text{-}HT7}$ intensity than $\alpha S\text{-}Tau^{LB509\text{-}HT7}$. However, no statistical significance was observed in the control group due to the small sample size and high sample-to-sample variation. Paired Wilcoxon test was applied for statistics. Source data are provided as a Source Data file.

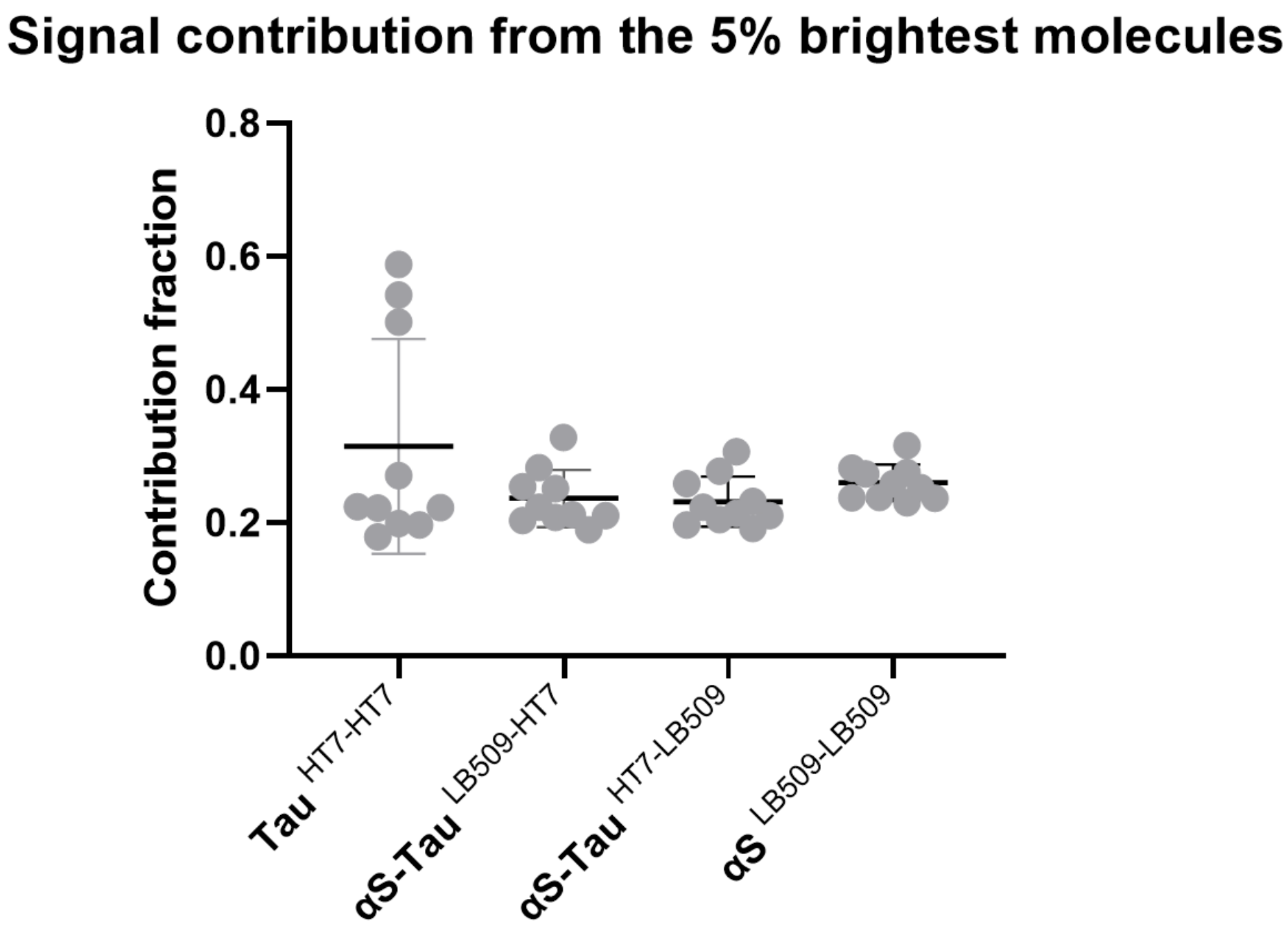

Supplementary Figure 12: Contribution of the top 5% brightest single molecules to the total signal. Each data point represents an individual sample. Across all detected species, this signal contribution ranges from approximately 25% to 40%, highlighting the significant influence of bright molecules on the overall signal and reflecting the inherent heterogeneity of the aggregates. (Data are expressed as mean ± SD. N=10 from all 10 brain homogenate samples) Source data are provided as a Source Data file.

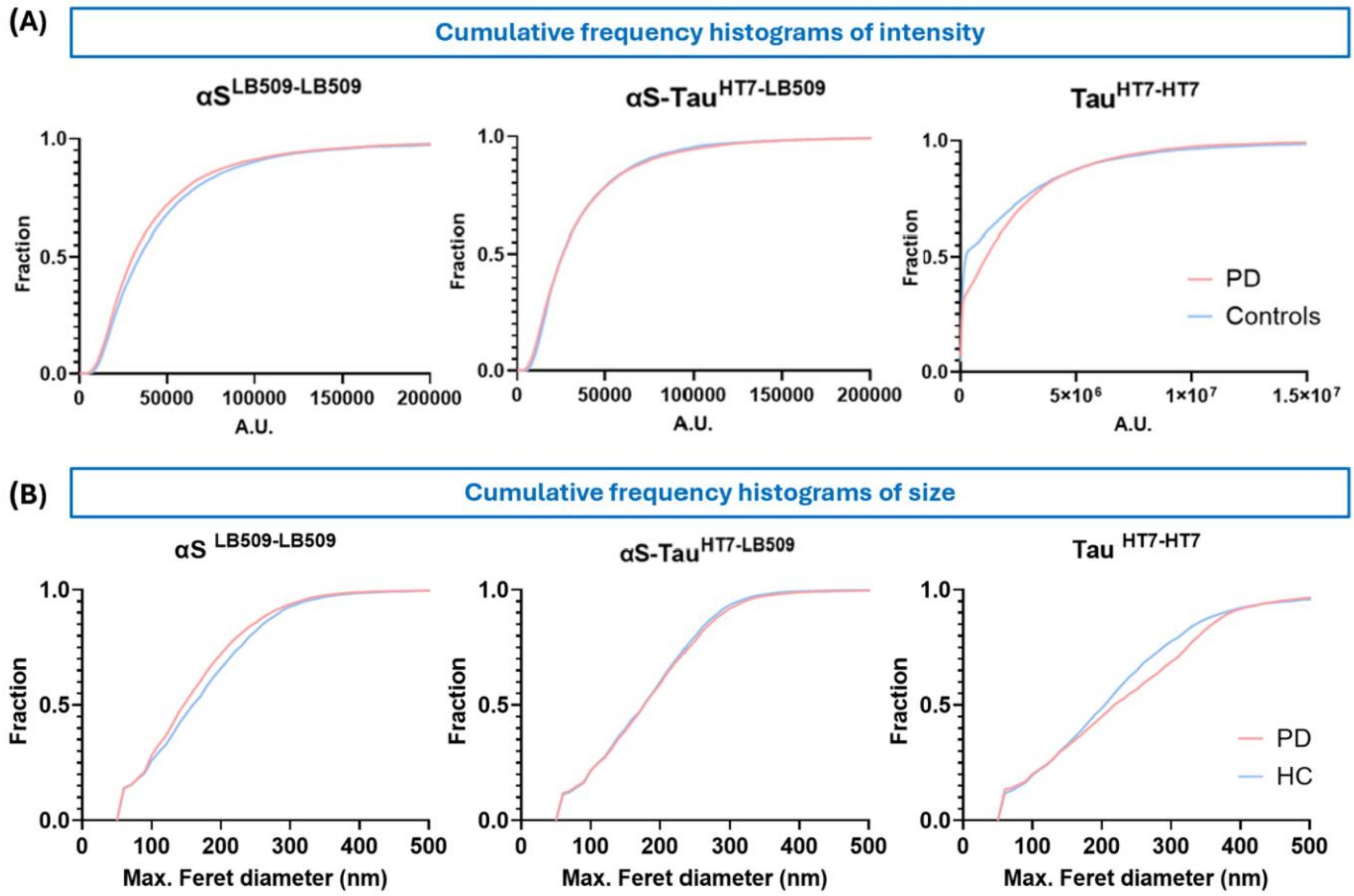

Supplementary Figure 13: Cumulative histogram of single molecule intensities and size. (A) The PD samples have more dim $\alpha S^{LB509\text{-}LB509}$ (A.U. < 1.3×10^5) and more bright $Tau^{HT7\text{-}HT7}$ molecules (A.U. > 4×10^6). No difference was observed for $\alpha S\text{-}Tau^{HT7\text{-}LB509}$. The histogram for $\alpha S\text{-}Tau^{LB509\text{-}HT7}$ was excluded due to high variability in single-molecule counts within the PD group, where half the samples had threefold higher counts compared to the other half (Fig. 4A). Since the sample size was relatively small and this trend was not evident in the control group, this analysis was omitted. (B). Averaged cumulative histogram of size between PD and control group. PD samples contained more small $\alpha S^{LB509\text{-}LB509}$ molecules (100 nm – 300 nm) and more bigger $Tau^{HT7\text{-}HT7}$ molecules (200 nm – 400 nm). Source data are provided as a Source Data file.

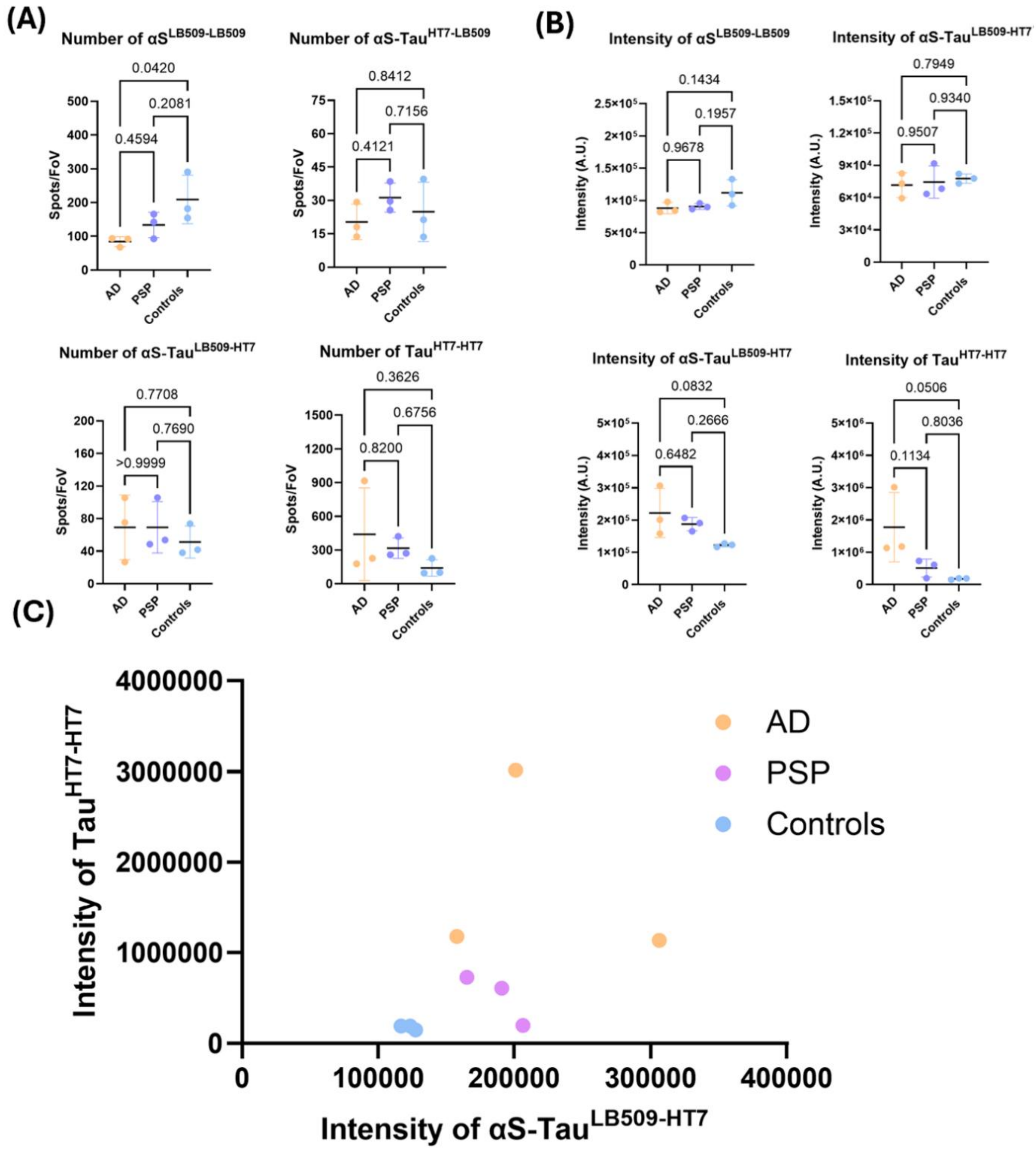

Supplementary Figure 14: Single-molecule quantification of protein assemblies in AD, PSP, and control brain homogenate. (A) Single-molecule counts of different protein assemblies. Overall, little difference was observed, except for increased $\alpha S^{LB509–LB509}$ counts in AD compared with controls (Ordinary one-way ANOVA). (B) Single-molecule intensities of protein assemblies. No significant differences were observed across groups. (C) Combined analysis of $\alpha S–Tau^{LB509–HT7}$ and $Tau^{HT7–HT7}$ intensities allows clear distinction among AD, PSP, and control samples. Data are expressed as mean ± SD. N=3 brains for each sample group. Source data are provided as a Source Data file.

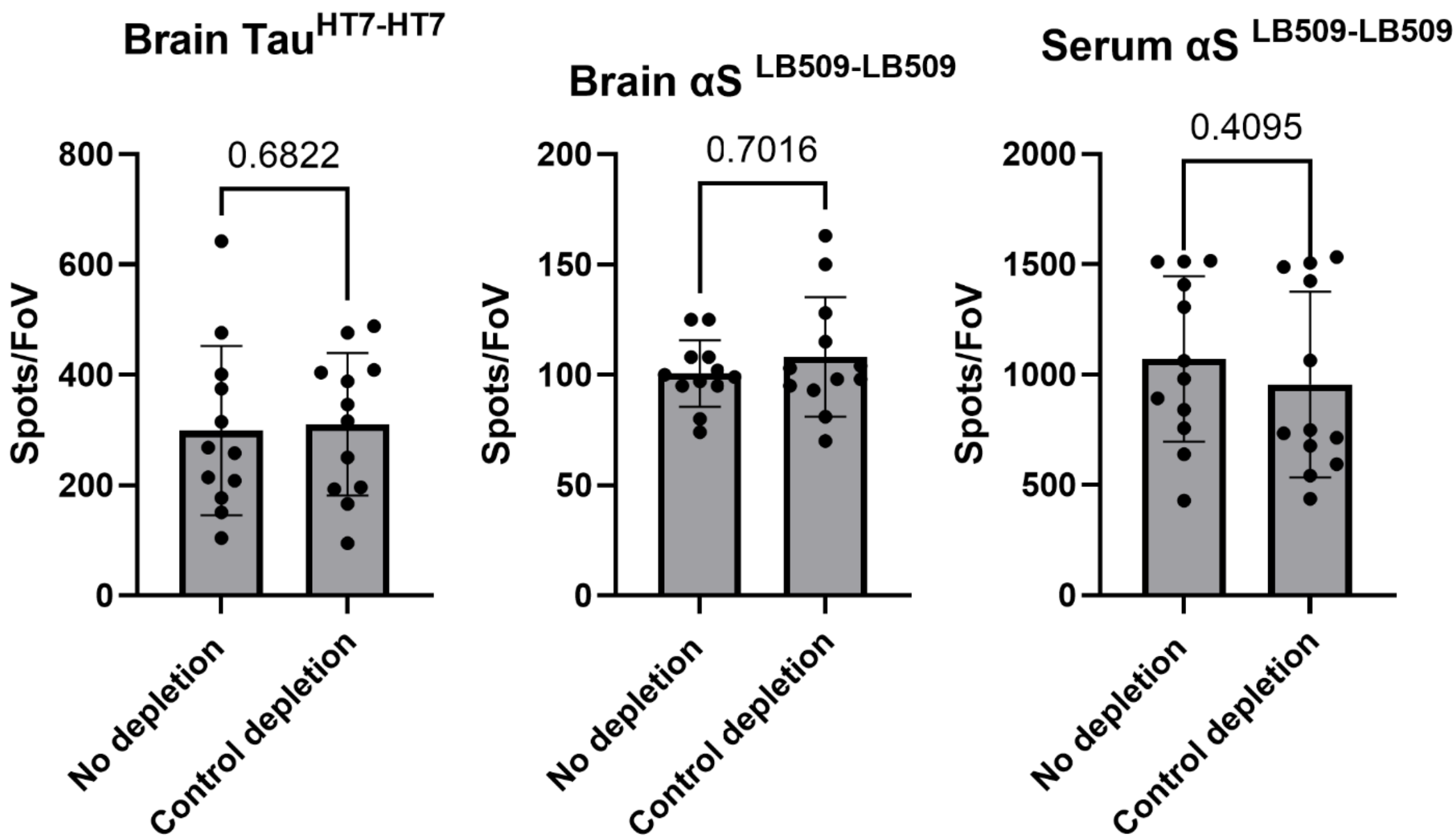

Supplementary Figure 15: Immunodepletion control experiment to validate specificity. Beads coupled with IgG were used to assess non-specific adsorption during precipitation. No significant reduction in signal was observed. (Data are expressed as mean ± SD. n=12 from 12 FoVs). Non-parametric Mann-Whitney test was used. Source data are provided as a Source Data file.

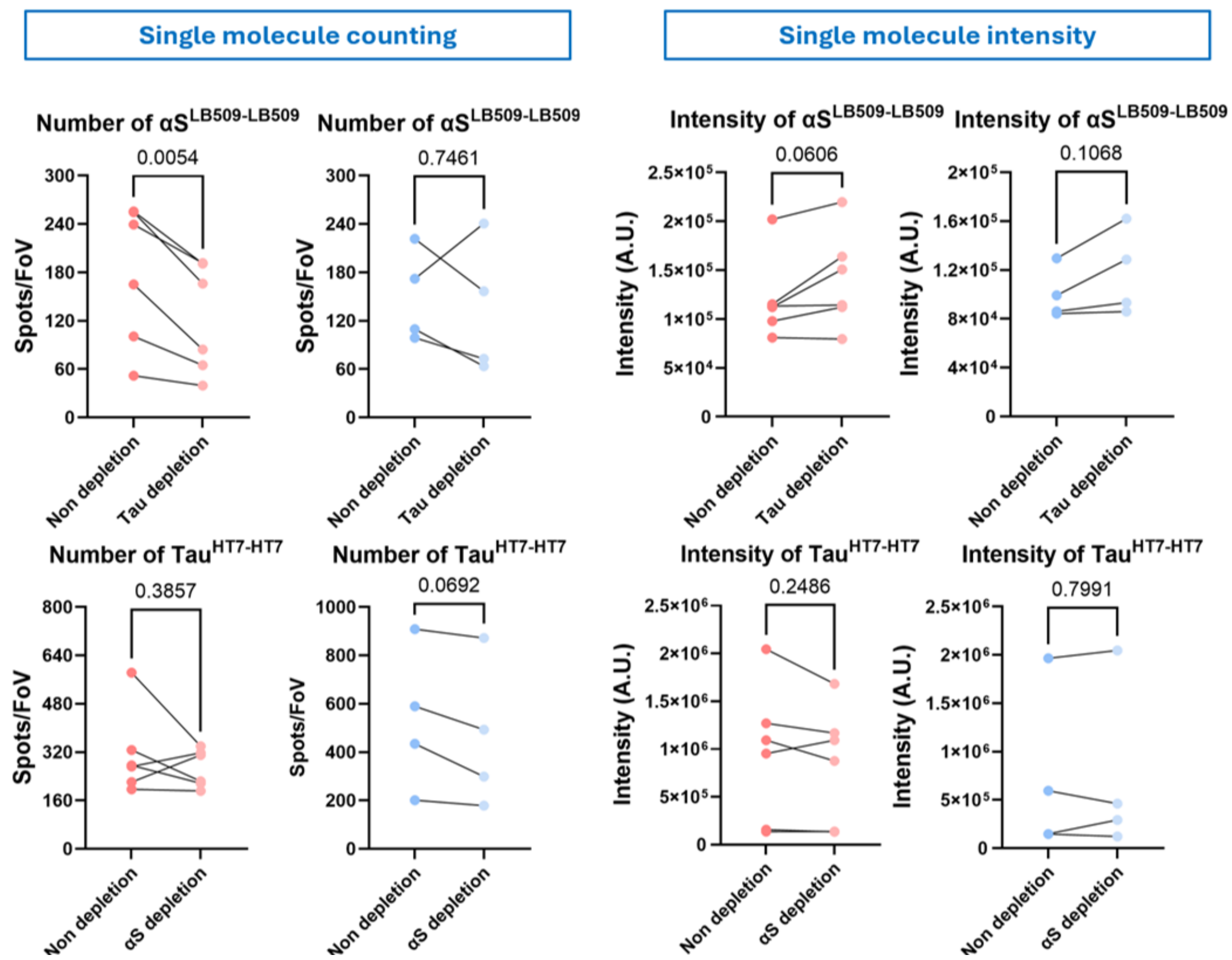

Supplementary Figure 16: Quantification of αS and Tau aggregates in brain homogenate following immunodepletion. Tau depletion reduced the number of αS aggregates in all PD samples and in 3 of 4 control samples. It also increased the intensity of αS aggregates in 5 of 6 PD samples and in all control samples. In contrast, αS depletion produced less consistent changes in signal. Paired Wilcoxon test was applied for statistics. Source data are provided as a Source Data file.

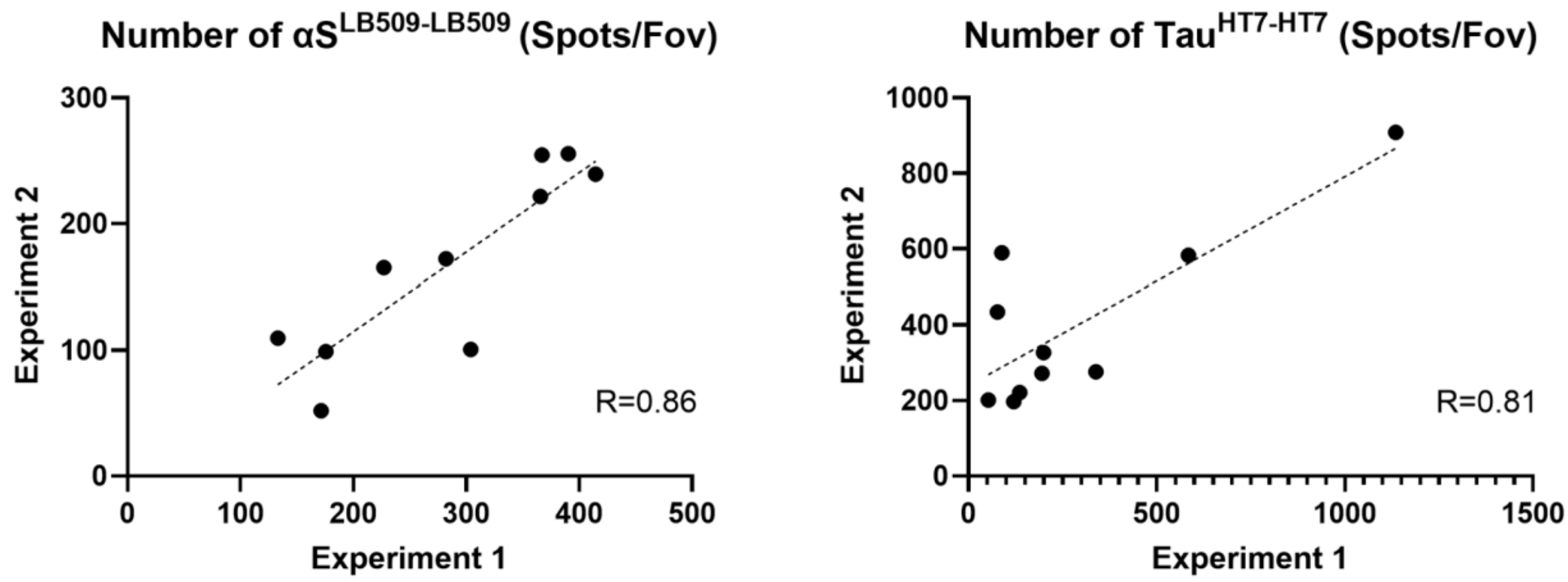

Supplementary Figure 17: Correlation between two independent measurements of protein aggregates in brain homogenate. The second measurement, conducted 15 months after the first, showed a strong correlation with the initial measurement. This measurement is coupled with proteinase K digestion and immunodepletion, which were not performed in experiment 1. Source data are provided as a Source Data file.

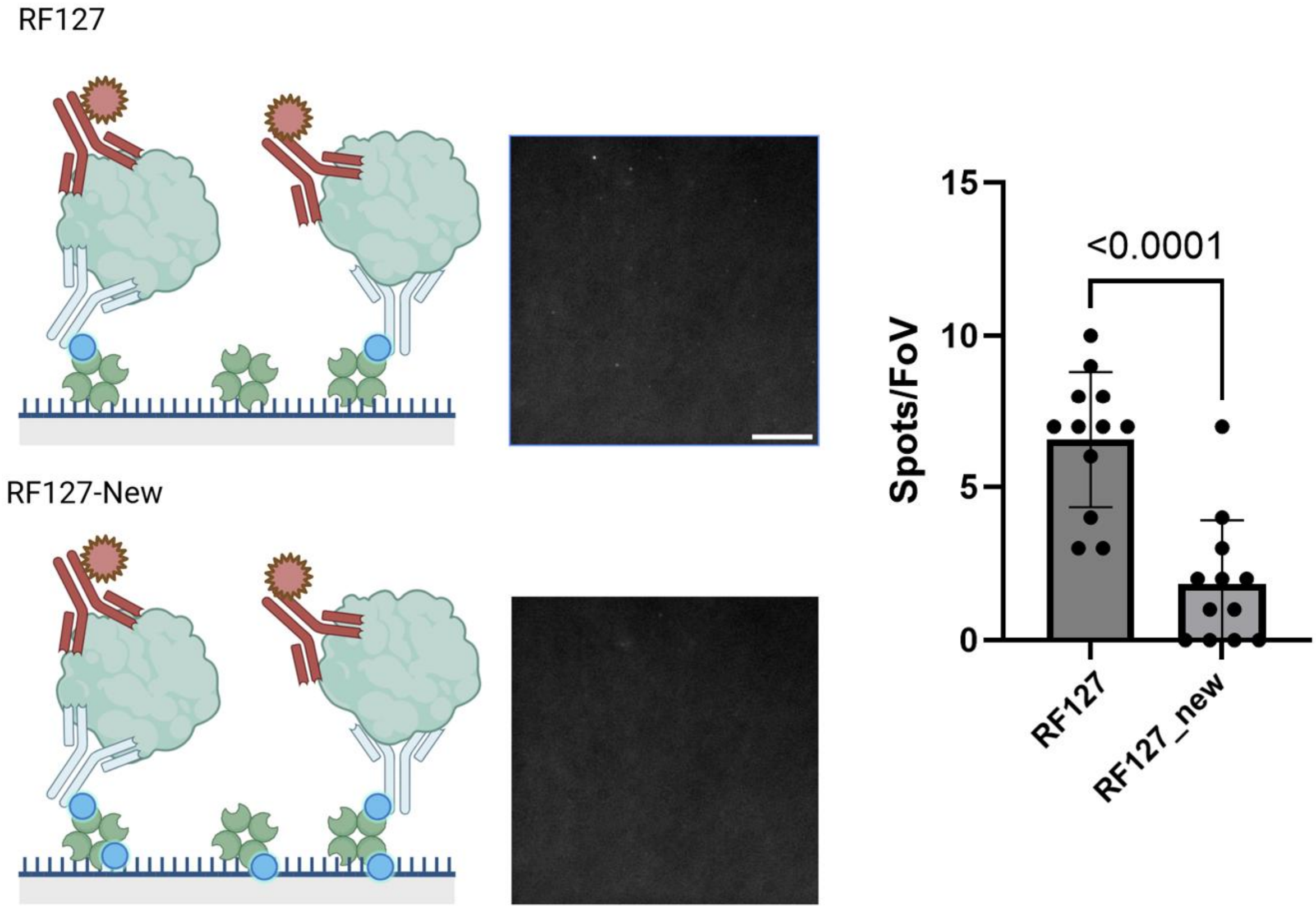

Supplementary Figure 18: Improved surface coating for SiMPull. In the conventional RF127 method, NeutrAvidin was directly coated onto a Rain-X–treated surface to provide specific binding sites. The passivation was achieved following later F-127 coating. We found that passivation can be further improved by using biotinylated F-127. In this optimised approach, the Rain-X–coated surface was treated with a mixture of F-127 and biotinylated F-127 to achieve selective passivation. While both methods effectively reduce non-specific binding (<10 spots/FoV for blank samples, e.g., sample diluent for soluble brain fraction), the new method further reduces non-specific binding by ~60%, which is critical when detecting very low signal levels. (Data are expressed as mean ± SD. n=12 from 12 FoVs). Non-parametric Mann-Whitney test was used. The figure created via BioRender. Zhang, Y. (2026) https://BioRender.com/e63sj4f. Source data are provided as a Source Data file.

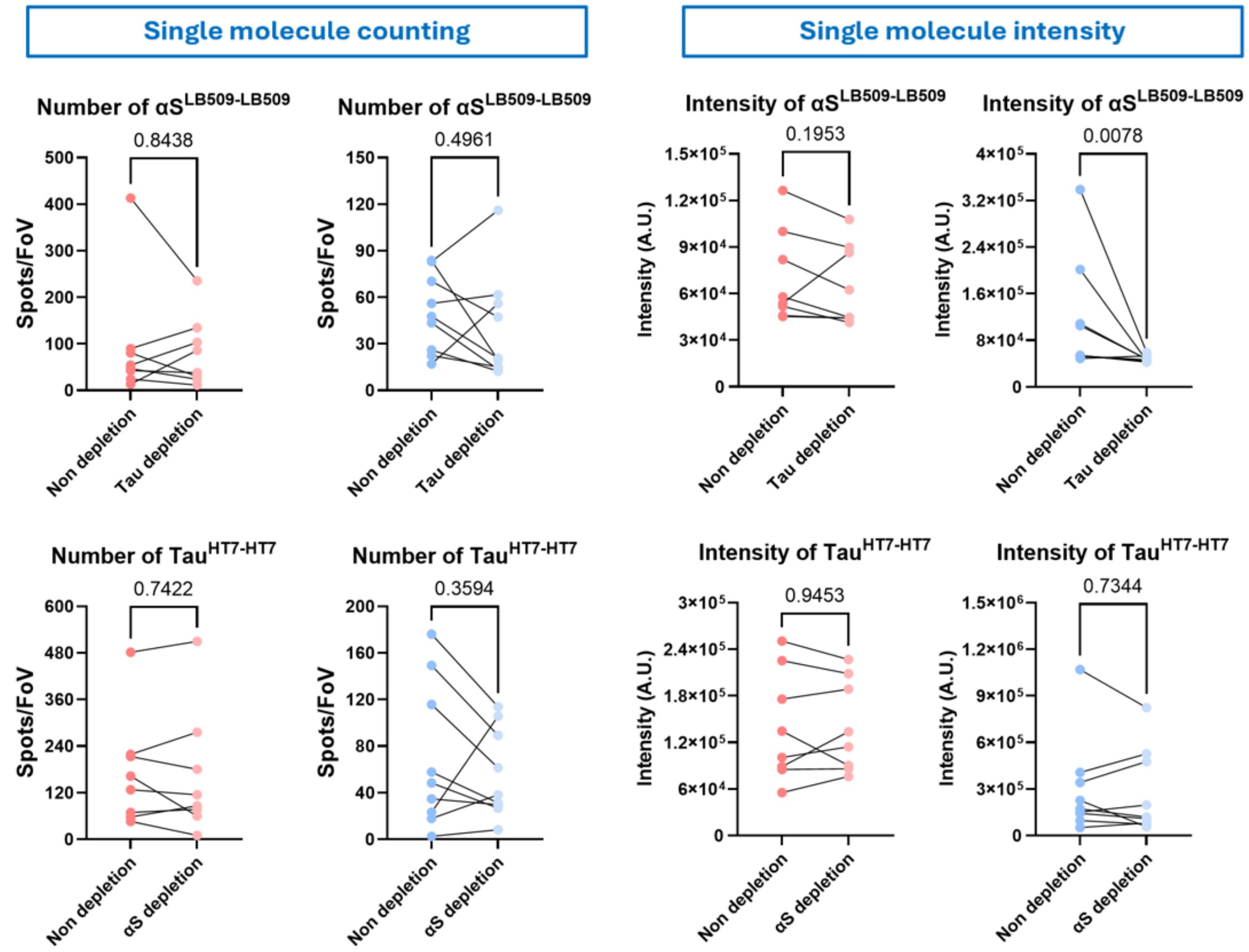

Supplementary Figure 19: Quantification of αS and Tau aggregates in the soluble fraction of brain-derived proteins following immunodepletion. No clear differences were observed in most measurements, except for a reduction in $\alpha S^{LB509\text{–}LB509}$ intensity after Tau depletion in control samples. Unlike brain homogenate, approximately 30% of samples showed an increased number of aggregates after depletion in both PD and control groups, indicating that immunodepletion does not substantially alter protein aggregates in the soluble fraction. Paired Wilcoxon test was applied for statistics. Source data are provided as a Source Data file.

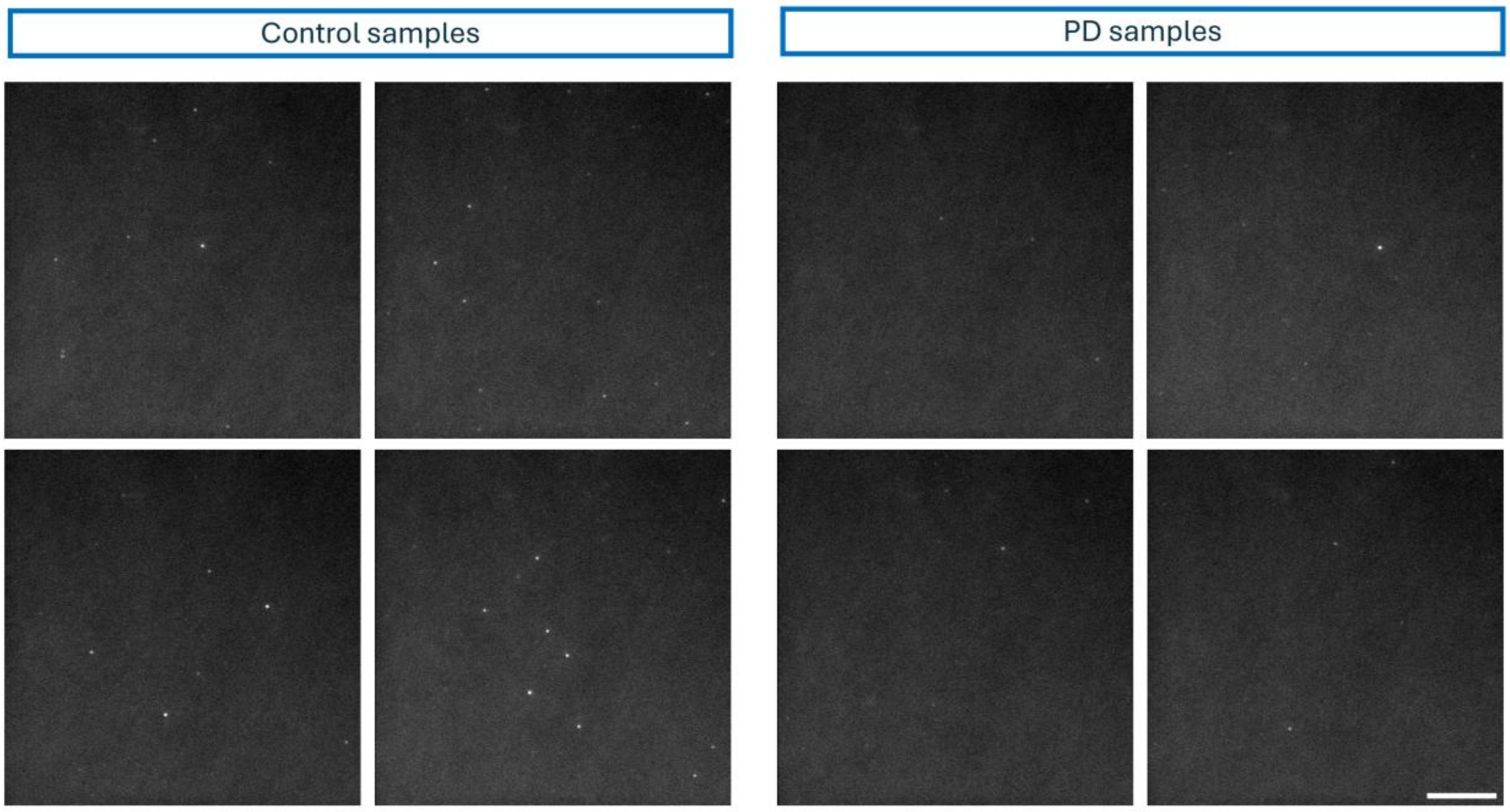

Supplementary Figure 20: Representative images of αS-Tau$^{HT7\text{-}LB509}$ in soluble fraction of brain proteins. Each image corresponds to a different sample. Although the overall signal is weak, the improved surface passivation substantially reduces non-specific binding, enabling sensitive quantification even at low concentrations.

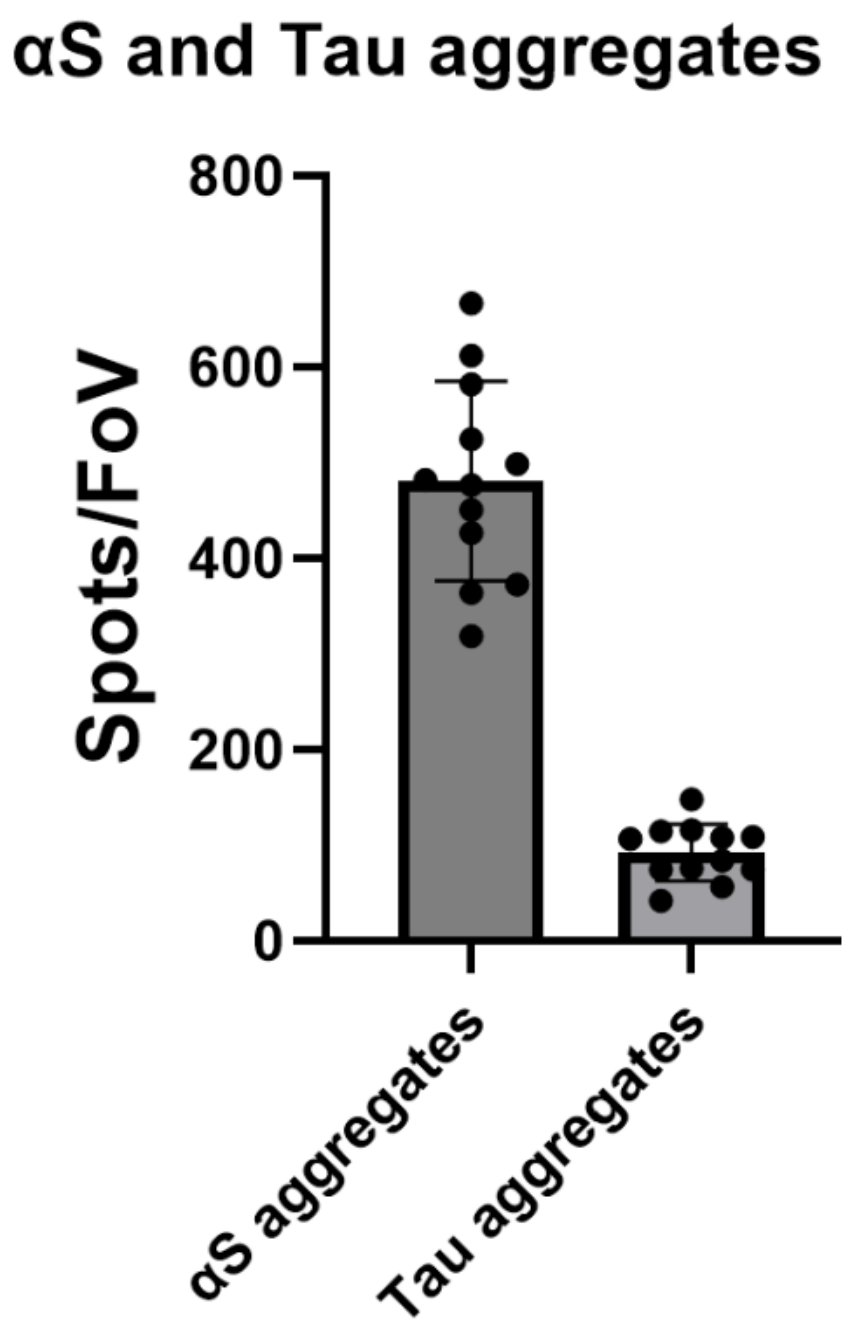

Supplementary Figure 21: Number of detected αS and Tau aggregates in human serum. αS aggregates were detected with Syn211 antibody as both capture and detection. Tau aggregates were detected with HT7 antibody as both capture and detection. (Data are expressed as mean ± SD. n =12, 12 FoVs) Source data are provided as a Source Data file.

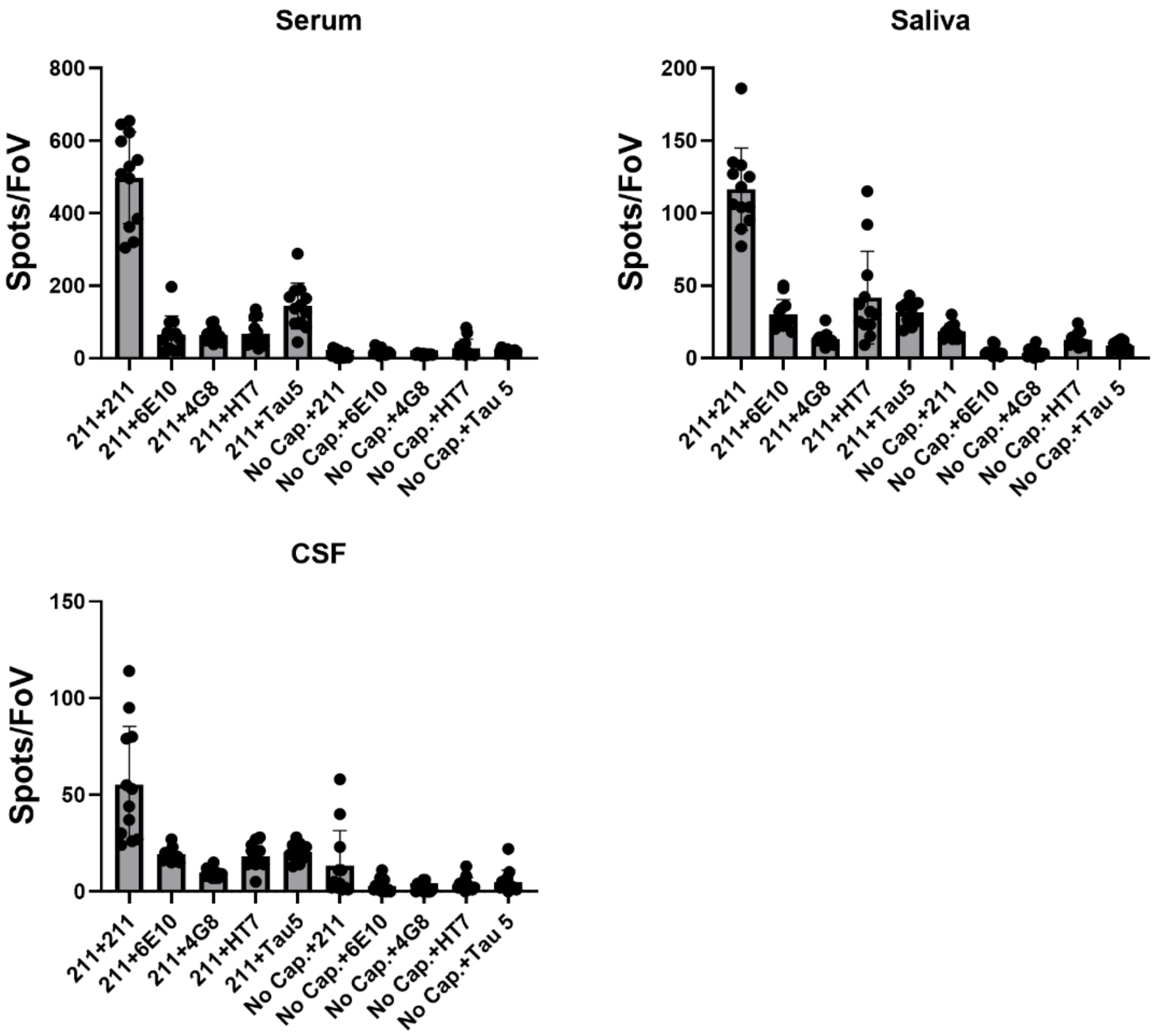

Supplementary Figure 22: Capture control experiments with different pooled biofluids. These experiments were conducted to verify the specificity of the capture antibody. Signal was observed only in the presence of a functioning capture antibody, confirming that detection was dependent on the antibody-antigen interaction. (Data are expressed as mean ± SD. n =12, 12 FoVs) Source data are provided as a Source Data file.

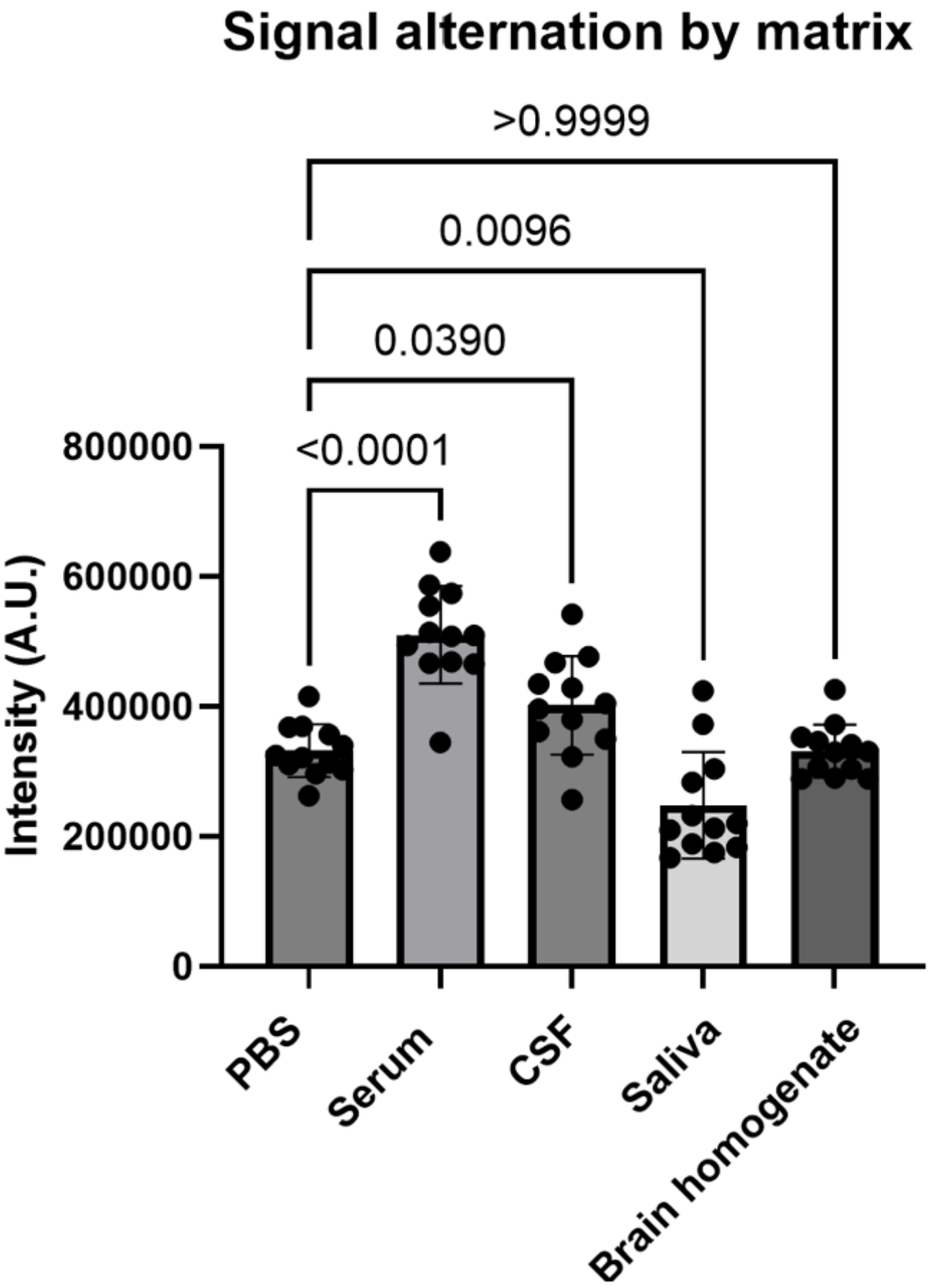

Supplementary Figure 23: Signal alteration by the matrix in biofluids. Briefly, the αS aggregates were immobilised on the surface with detection antibodies attached. Different biofluids were added afterwards to assess how the matrix would alter the fluorescent signal. Brain homogenate showed the least alternation among all biofluids. (Data are expressed as mean ± SD. n =12, 12 FoVs) Ordinary one-way ANOVA with post-hoc multiple-comparison correction. Source data are provided as a Source Data file.

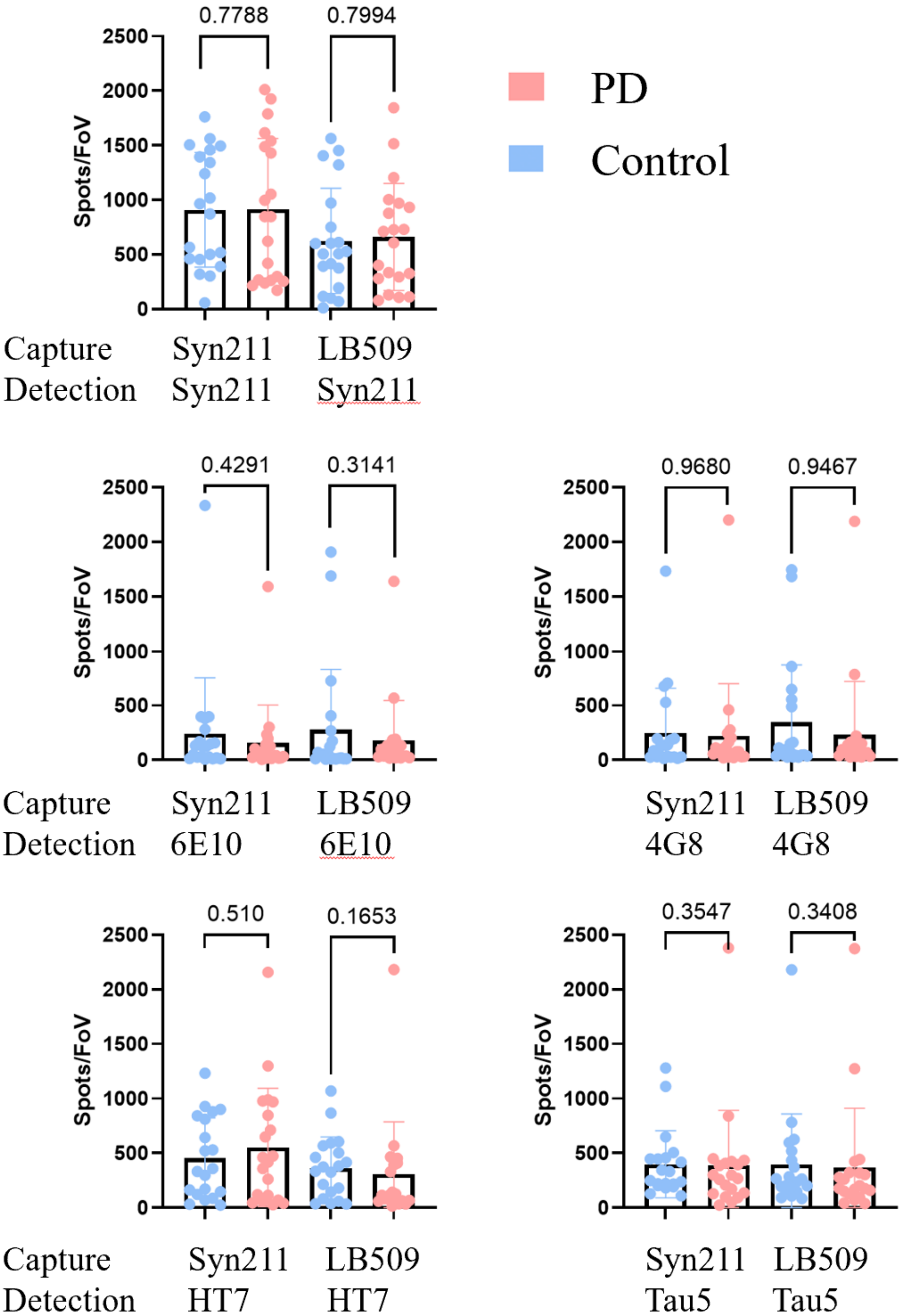

Supplementary Figure 24: Single-molecule counting of serum samples with different antibody pairs. 20 PD and 20 control samples were involved. The ratio data presented in Fig.7A was derived from this data set. Data are expressed as mean ± SD. Non-parametric Mann-Whitney test was used. Source data are provided as a Source Data file.

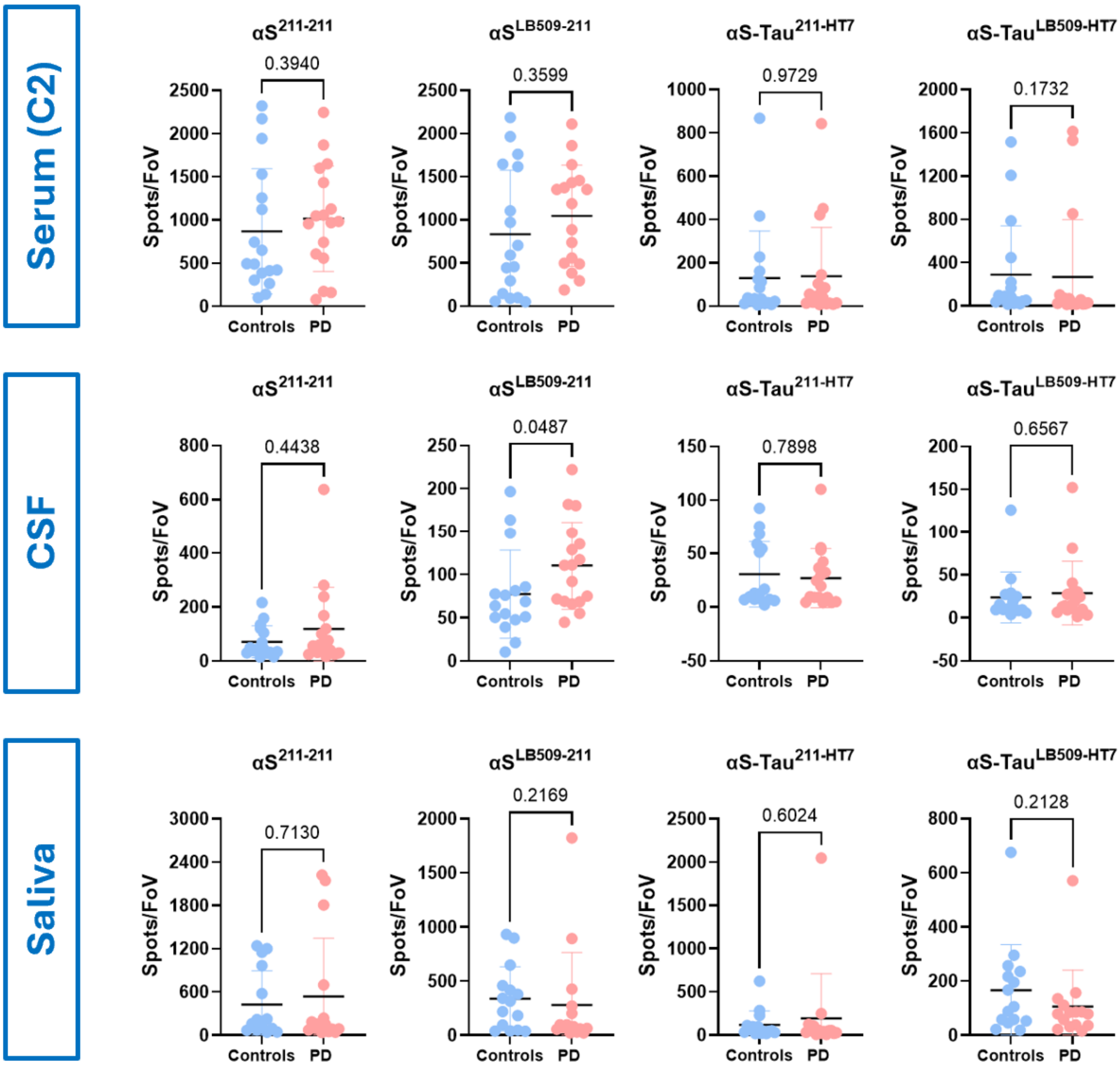

Supplementary Figure 25: Single-molecule counting of serum (cohort 2), CSF and saliva samples with different antibody pairs. 17 PD and 17 control samples were involved in serum cohort. 17 PD and 16 control samples were involved in CSF cohort. 15 PD and 15 control samples were involved in Saliva cohort. The ratio data presented in Fig.7 was derived from this data set. Data are expressed as mean ± SD. Non-parametric Mann-Whitney test was used. Source data are provided as a Source Data file.

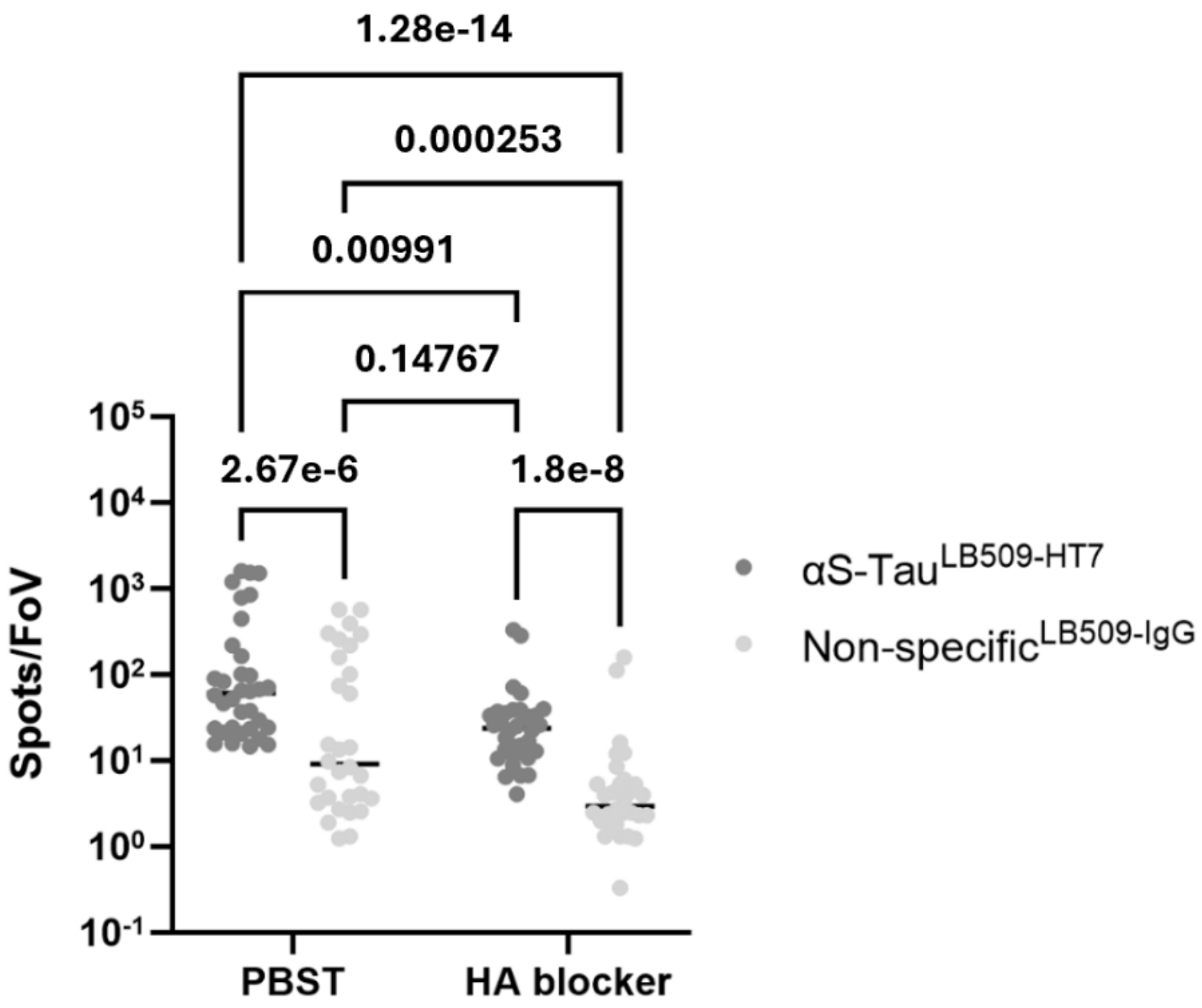

Supplementary Figure 26: Single-molecule counting comparison between signals obtained with IgG isotype control and HT7 detection. Overall, HT7 antibody produces significantly higher signals than the IgG isotype control without or without the presence of HA blocker. (s.d. N=34 both PBST group and HA blocker group). The Aligned Rank Transform (ART) ANOVA was performed with Aligned Rank Transform Contrasts (ART-C) post hoc multiple comparisons and Tukey's adjustment. Source data are provided as a Source Data file.

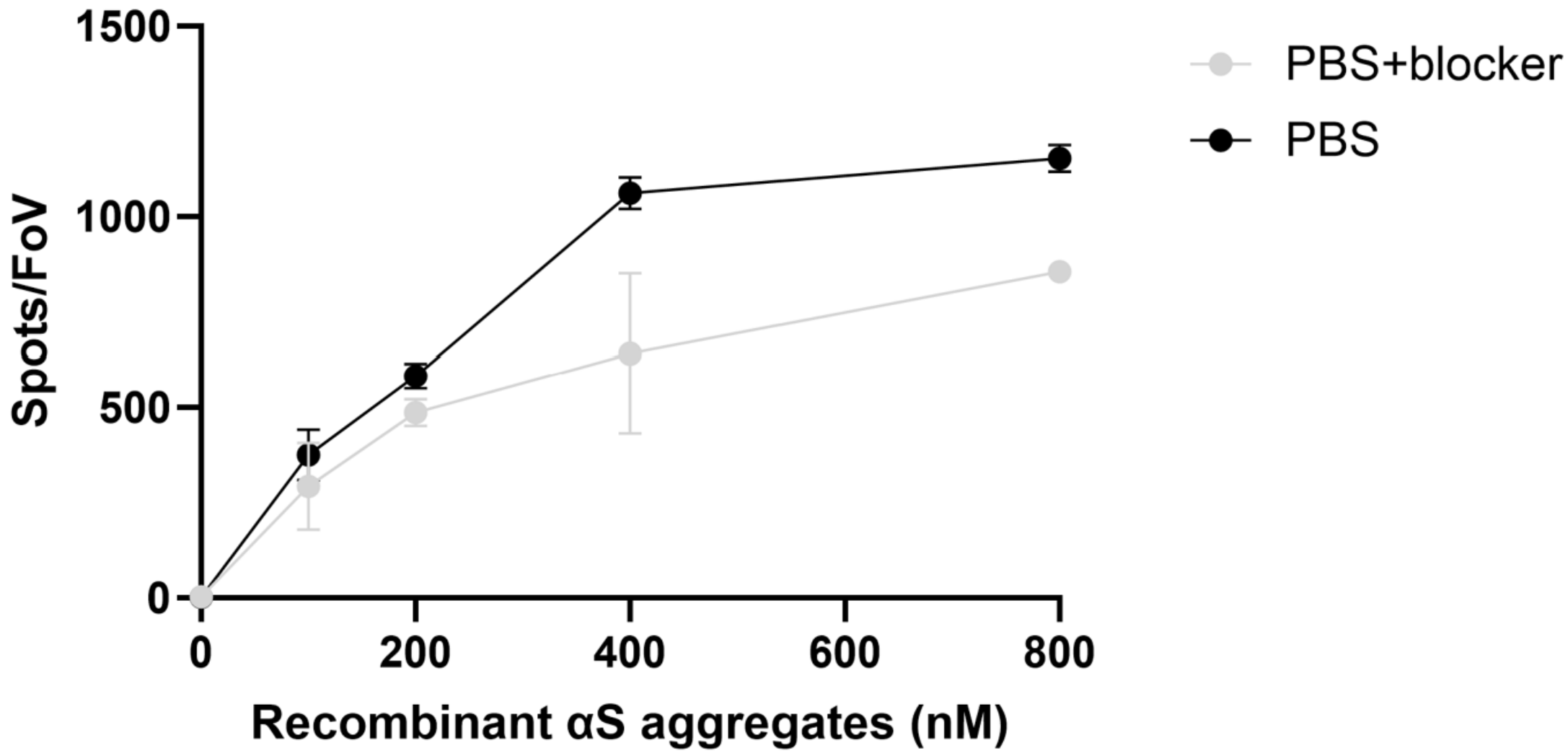

Supplementary Figure 27: SiMPull signal comparison using different diluents. The addition of HA blocker reduces the specific binding of recombinant αS. As this experiment uses a single-protein system, non-specific protein binding is negligible. (Data are expressed as mean ± SD. n=12 from 12 FoVs) Source data are provided as a Source Data file.

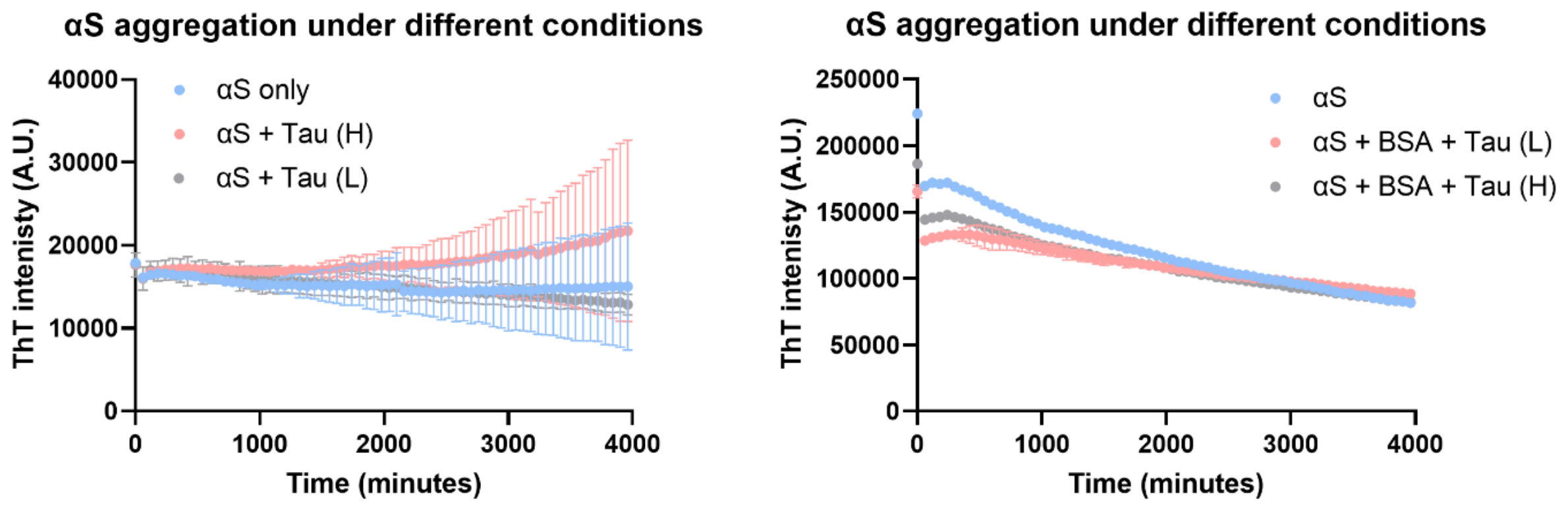

Supplementary Figure 28: ThT fluorescence assay of αS aggregation under different conditions. Higher Tau concentration (0.05 mg/ml) enhances αS aggregation. However, BSA shows apparent ThT fluorescence, which may interfere with the assay. (Data are expressed as mean ± SD. n=3 from 3 measurements) Source data are provided as a Source Data file.

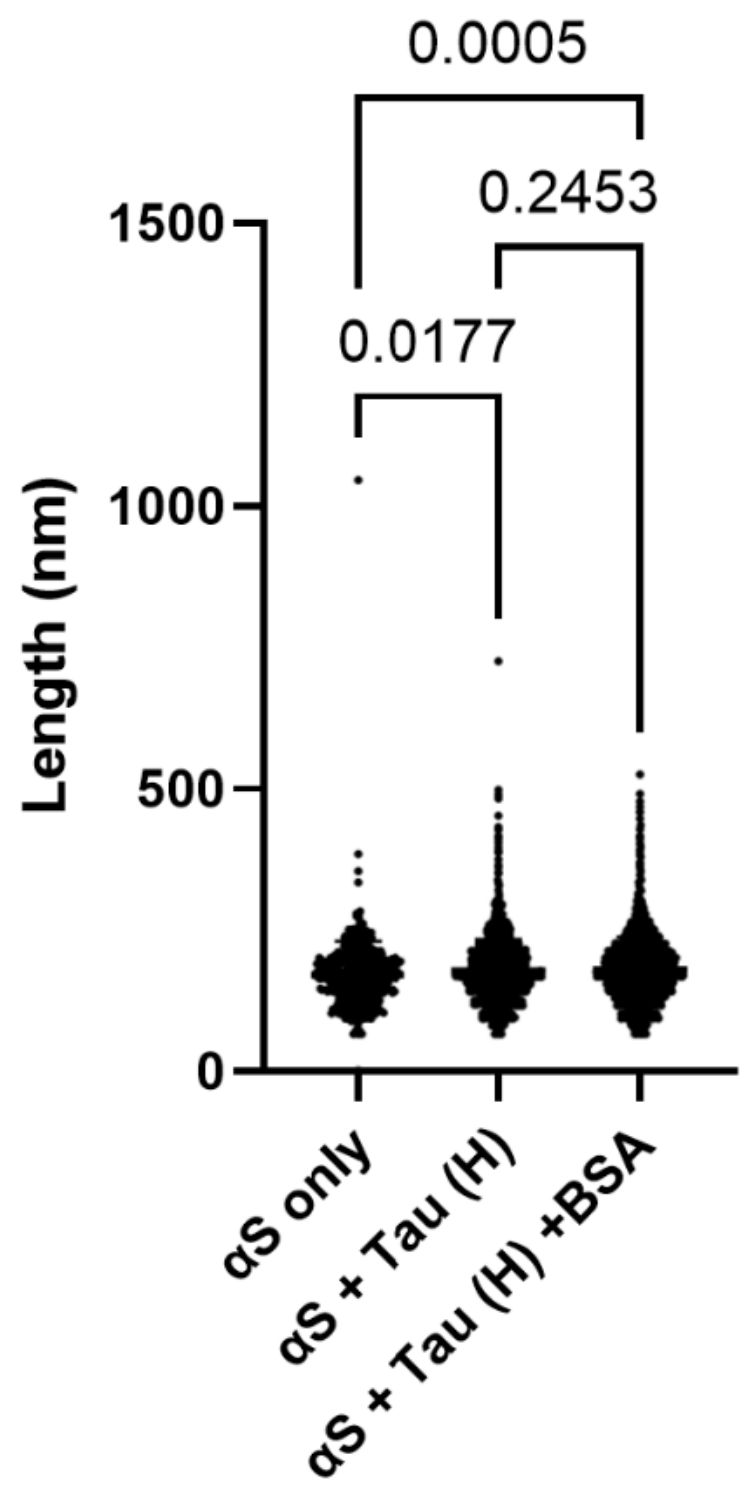

Supplementary Figure 29: Single-molecule size of αS aggregates formed under αS-only, αS + Tau (H) and αS + Tau (H) + BSA conditions. αS aggregates formed in the presence of Tau (H) were significantly larger than those formed under αS-only conditions. Addition of BSA did not alter this trend. A Kruskal–Wallis test was used for statistical analysis due to large n number. (n=589 individual aggregates for αS-only, n=2410 individual aggregates for αS + Tau (H), n= 3711 individual aggregates for αS + Tau (H) + BSA).

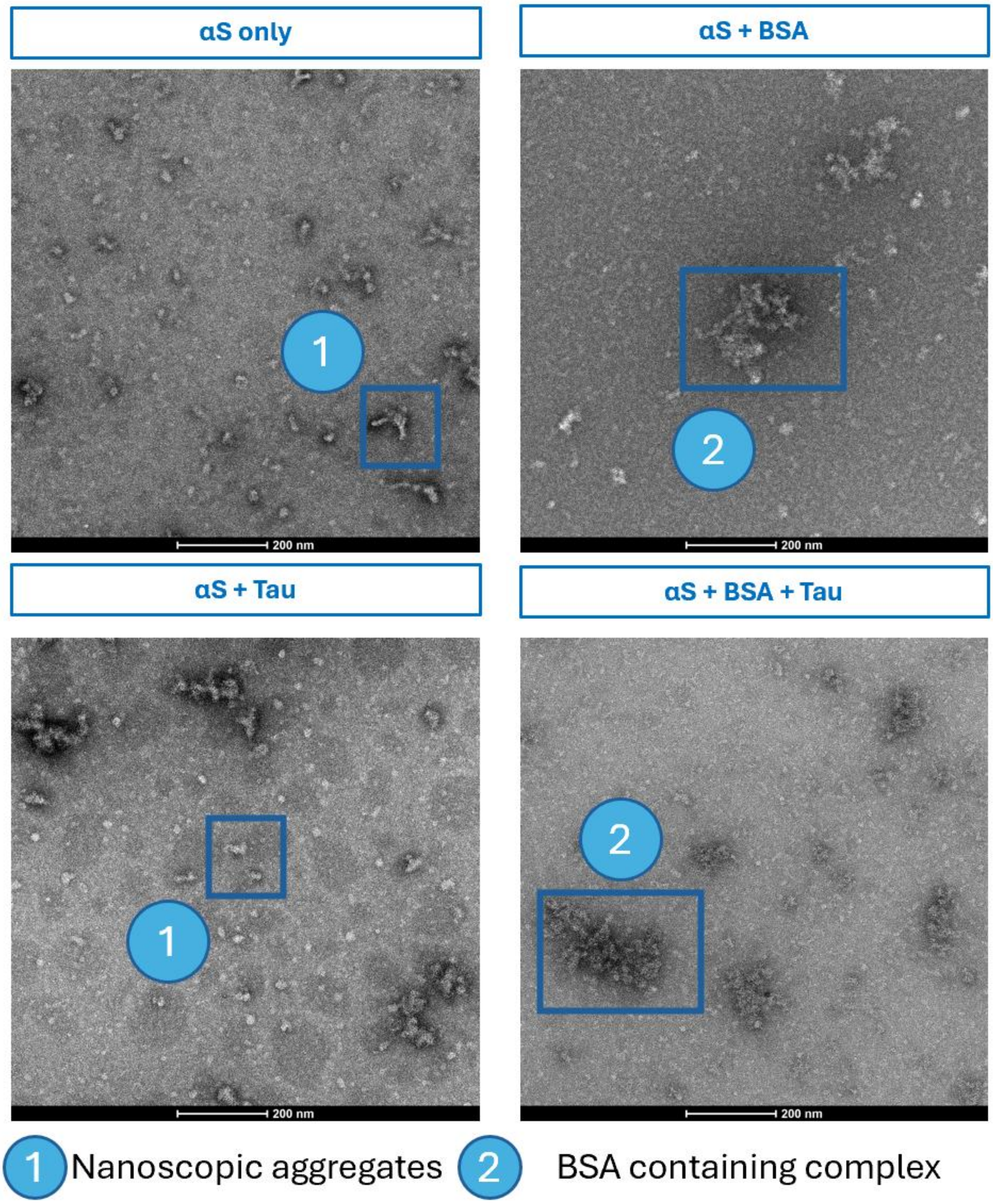

Supplementary Figure 30: TEM images of protein assemblies formed under different conditions. At lower αS concentrations, aggregates form without clear long-fibrillar structures. Tau interacts with αS to produce cluster-like assemblies containing filament-like subunits. The addition of BSA promotes the formation of larger, amorphous aggregates.

Supplementary Table 1 antibodies used in the work

Target species	Antibody name	Source
	Biotinylated Syn-211	sc-12767, santa cruz biotech, with modification*
αS	AF647 Syn-211	sc-12767 AF647, santa cruz biotech
	Biotinylated LB509	807710, BioLegend
	AF647 LB509	sc-58480 AF647, santa cruz biotech
	Biotinylated 6E10	803008, BioLegend
	AF647 6E10	803021, BioLegend
Aβ	AF488 6E10	803013, BioLegend
	AF647 4G8	800718, BioLegend
	Biotinylated HT7	MN1000B,Invitrogen
Tau	AF647 HT7	51-5916-42, Invitrogen
	AF647 Tau5	AHB0042, Invitrogen with modification*
	Biotinylated IgG	MG115, Invitrogen
IgG control	AF647 IgG	MOPC-21 ,AB_2539542, Invitrogen

Supplementary Table 2 Diluent and incubation time for different samples

Sample name	Diluent	Incubation time
Recombinant αS and αS-Aβ	PBST	30 min
Human serum (cohort1)	PBST (1:15)	90 min
Human serum (cohort2)	PBST (1:10)	90 min
Human serum (cohort2 + HA blocker)	PBST + 1% TRU block (OAMA04082),(1:10)	90 min
Human Brain homogenate	ELISA Diluent (1:2)*	120 min
Human Brain (Soluble)	ELISA Diluent (1:5)*	120 min
Human CSF	PBST (1:2)	120 min
Human Saliva	PBST (1:10)	90 min

ELISA diluent was purchased from Abcam. (ab193972)

Supplementary Table 3 Demographic and clinical characteristics of the serum sample donors (cohort 1)

	Control	PD	*p* value
Sample size	20	20	1
Age (years)	65.0 ± 10.0	65.7 ± 6.1	0.4
Sex (% male)	45%	60%	0.8
Disease duration (years)	-	0.8 ± 0.4	-
Hoehn & Yahr	-	1.7 ± 0.6	-
MDS-UPDRS III	-	27.0 ± 11.5	-
MDS-UPDRS Total	-	47.3 ± 21.8	-

Values are mean ± SD unless otherwise stated. Missing values were excluded. P values: Welch's t-test for age and Fisher's exact test for sex.

Supplementary Table 4 Demographic and clinical characteristics of the serum sample donors (cohort 2)

	Control	PD	*p* value
Sample size	17	17	1
Age (years)	66.2 ± 8.2	64.9 ± 7.2	0.6
Sex (% male)	53%	76%	0.2
Disease duration (years)	-	1.0 ± 1.0	-
Hoehn & Yahr	-	1.9 ± 0.3	-
MDS-UPDRS III	-	31.4 ± 9.6	-
MDS-UPDRS Total	-	51.4 ± 20.6	-

Values are mean ± SD unless otherwise stated. Missing values were excluded. P values: Welch's t-test for age and Fisher's exact test for sex.

Supplementary Table 5 Demographic and clinical characteristics of the saliva samples

	Control	PD	*p* value
Sample size	15	15	1
Age (years)	68.8 ± 6.3	67.0 ± 5.1	0.4
Sex (% male)	47%	60%	0.5
Disease duration (years)	-	1.6 ± 2.0	-
Hoehn & Yahr	-	1.8 ± 0.6	-
MDS-UPDRS III	-	29.3 ± 10.0	-
MDS-UPDRS Total	-	50.7 ± 18.9	-

Values are mean ± SD unless otherwise stated. Missing values were excluded. P values: Welch's t-test for age and Fisher's exact test for sex.

Supplementary Table 6 Demographic and clinical characteristics of the CSF samples

	Control	PD	*p* value
Sample size	16	17	1
Age (years)	71.7 ± 7.6	67.7 ± 8.1	0.2
Sex (% male)	56%	59%	0.7
Disease duration (years)	-	2.2 ± 1.4	-
Hoehn & Yahr	-	2.0 ± 0.4	-
MDS-UPDRS III	-	32.3 ± 12.1	-
MDS-UPDRS Total	-	51.6 ± 19.2	-

Values are mean ± SD unless otherwise stated. Missing values were excluded. P values: Welch's t-test for age and Fisher's exact test for sex.

Supplementary Table 7 Demographic and clinical characteristics of the PD and control brain donors (homogenate cohort)

	Control	PD	*p value*
Sample size	4	6	-
Age at death (years)	54.0 ± 17.6	79.7 ± 8.2	0.05
Sex (% male)	75%	67%	1.00
Post-mortem interval (hours)	47.0 ± 29.0	59.5 ± 37.3	0.57
Lewy body Braak stage	0.0 ± 0.0	5.8 ± 0.4	0.007
Tau tangle Braak stage	0.3 ± 0.5	2.3 ± 0.6	0.04
Disease duration (years)	-	18.1 (n=1)	-

Values are mean ± SD unless otherwise stated. Missing values were excluded. P values: Welch's t-test for age and post-mortem interval, Fisher's exact test for sex, and Mann-Whitney U test for Braak stages.

Supplementary Table 8 Demographic and clinical characteristics of the PD and control brain donors (soluble cohort)

	Control	PD	*p value*
Sample size	9	8	-
Age at death (years)	82.1 ± 11.5	80.4 ± 7.7	0.72
Sex (% male)	56%	38%	0.64
Post-mortem interval (hours)	46.1 ± 39.2	74.0 ± 35.0	0.14
Lewy body Braak stage	-	4.6 ± 0.5	-
Tau tangle Braak stage	-	5.4 ± 0.7	-
Disease duration (years)	-	22.8 ± 11.6	-

Values are mean ± SD unless otherwise stated. Missing values were excluded. P values: Welch's t-test for age and post-mortem interval, Fisher's exact test for sex, and Mann-Whitney U test for Braak stages.

Supplementary Table 9 Demographic and clinical characteristics of the AD, PSP and control brain donors (tauopathy homogenate cohort)

	Control	AD	PSP	*p value*
Sample size	3	3	3	-
Age at death (years)	67.3 ± 1.2	75.0 ± 5.6	74.3 ± 5.9	0.17
Sex (% male)	67%	0%	33%	0.68
Post-mortem interval (hours)	44.7 ± 40.1	33.7 ± 21.5	61.7 ± 32.6	0.59
Lewy body Braak stage	-	-	-	-
Tau tangle Braak stage	-	6.0 ± 0.0	4.3 ± 0.6	-
Disease duration (years)	-	6.0 ± 1.4	5.7 ± 1.2	-

Values are mean ± SD unless otherwise stated. Missing values were excluded. P values: one-way ANOVA for age and post-mortem interval and an exact 3 × 2 test for sex.
