# Supplementary material for: Imaging nanoscopic co-assemblies of α-synuclein and Tau in human samples
Source: Nat Commun. 2026 Sep 18;17:10951. doi: 10.1038/s41467-026-77728-1 (PMC13680374; doi:10.1038/s41467-026-77728-1)
Supplement: Supplementary file 2 [file 41467_2026_77728_MOESM2_ESM.pdf]

nature portfolio

Corresponding author(s): David Klenerman, Yu P. Zhang

Last updated by author(s): 27/07/2026

Reporting Summary

Nature Portfolio wishes to improve the reproducibility of the work that we publish. This form provides structure for consistency and transparency in reporting. For further information on Nature Portfolio policies, see our Editorial Policies and the Editorial Policy Checklist.

Statistics

For all statistical analyses, confirm that the following items are present in the figure legend, table legend, main text, or Methods section.

n/a	Confirmed	
☐	☒	The exact sample size (n) for each experimental group/condition, given as a discrete number and unit of measurement
☐	☒	A statement on whether measurements were taken from distinct samples or whether the same sample was measured repeatedly
☐	☒	The statistical test(s) used AND whether they are one- or two-sided<br>*Only common tests should be described solely by name; describe more complex techniques in the Methods section.*
☒	☐	A description of all covariates tested
☐	☒	A description of any assumptions or corrections, such as tests of normality and adjustment for multiple comparisons
☐	☒	A full description of the statistical parameters including central tendency (e.g. means) or other basic estimates (e.g. regression coefficient) AND variation (e.g. standard deviation) or associated estimates of uncertainty (e.g. confidence intervals)
☐	☒	For null hypothesis testing, the test statistic (e.g. F, t, r) with confidence intervals, effect sizes, degrees of freedom and P value noted<br>*Give P values as exact values whenever suitable.*
☒	☐	For Bayesian analysis, information on the choice of priors and Markov chain Monte Carlo settings
☒	☐	For hierarchical and complex designs, identification of the appropriate level for tests and full reporting of outcomes
☒	☐	Estimates of effect sizes (e.g. Cohen's d, Pearson's r), indicating how they were calculated

Our web collection on statistics for biologists contains articles on many of the points above.

Software and code

Policy information about availability of computer code

Data collection	MicroManager (Micro-Manager 2.0.0) open source software was used for data collection (imaging)
Data analysis	A customized FIJI(ImageJ) macro was used for single molecule counting/intensity analysis (https://github.com/YPZ858/DF-single-molecule-couting-). A Matlab script was used for super resolution imaging analysis (https://github.com/YPZ858/Super-res-code/issues). Statistical tests was performed with Graph Pad prism 9.0.

For manuscripts utilizing custom algorithms or software that are central to the research but not yet described in published literature, software must be made available to editors and reviewers. We strongly encourage code deposition in a community repository (e.g. GitHub). See the Nature Portfolio guidelines for submitting code & software for further information.

Data

Policy information about availability of data

All manuscripts must include a data availability statement. This statement should provide the following information, where applicable:

- Accession codes, unique identifiers, or web links for publicly available datasets
- A description of any restrictions on data availability
- For clinical datasets or third party data, please ensure that the statement adheres to our policy

The source data is provided with the paper. Codes used for analysis are also publicly available. (see methodology section)

Research involving human participants, their data, or biological material

Policy information about studies with human participants or human data. See also policy information about sex, gender (identity/presentation), and sexual orientation and race, ethnicity and racism.

Reporting on sex and gender	No analysis was performed on sex or gender. The data on sex of all study participants were collected based on self-report and presented in the maintext. Participants without neurological disease were statistically sex-matched to the patients with disease.
Reporting on race, ethnicity, or other socially relevant groupings	No race, ethnicity, or other socially relevant variables were included in our study.
Population characteristics	Participants with Parkinson's disease were assessed using the Movement Disorder Society Unified Parkinson's Disease Rating Scale (MDS-UPDRS). Parkinson's disease stage was determined using the Hoehn and Yahr scale.
Recruitment	Patients with idiopathic Parkinson's disease were recruited through the Parkinson's Disease Research Clinic at the John Van Geest Centre for Brain Repair, University of Cambridge/Cambridge University Hospitals NHS Trust, UK. Age and sex-matched participants without neurological disease were also recruited from the NIHR Cambridge Bioresource (http://www.cambridgebioresource.org.uk). For the soluble brain fraction, samples were collected under the protocol RA032611/1 (REC reference 18/LO/0721, "Queen Square Brain Bank for Neurological Disorders," University College London) at University College London.We selected a homogeneous group of PD patients together with age and gender-matched controls where possible.
Ethics oversight	Blood, saliva and CSF samples: Ethical approval was obtained from the East of England - Essex Research Ethics Committee (16/EE/0445), and East of England - Cambridge Central Research Ethics Committee (03/303, and 15/EE/0270). Informed consents was provided by all participants. The study protocol was also approved by the regional ethics committee at the University of Gothenburg.<br>Post-mortem brain donors: the post-mortem work was approved by the London—Bloomsbury Research Ethics Committee; 16/LO/0508. The brain samples have been voluntarily donated without any compensation and with the ethics allowing to release the associated anonymised, non-identifiable clinical and pathological metadata. For the soluble brain fraction, samples were collected under the protocol RA032611/1 (REC reference 18/LO/0721, "Queen Square Brain Bank for Neurological Disorders," University College London).

Note that full information on the approval of the study protocol must also be provided in the manuscript.

Field-specific reporting

Please select the one below that is the best fit for your research. If you are not sure, read the appropriate sections before making your selection.

- [x] Life sciences
- [ ] Behavioural & social sciences
- [ ] Ecological, evolutionary & environmental sciences

For a reference copy of the document with all sections, see nature.com/documents/nr-reporting-summary-flat.pdf

Life sciences study design

All studies must disclose on these points even when the disclosure is negative.

Sample size	No statistical tests were preformed to determine the sample of brain samples. The sample size of human serum (37 PD + 37 HC), saliva (15 PD + 15 HC) and CSF (17 PD + 16 HC) was determined by our previous research, where 10 PD and 10 HC samples were enough to generate statistical significance. (https://academic.oup.com/brain/article/145/2/632/6355020?login=true)
Data exclusions	No data were excluded.
Replication	Number of replicates are indicated in figure description.
Randomization	Imaging was performed automatically with a custom-written script for unbiased measurements.
Blinding	Blinding was not performed with the discovery cohort of human serum or recombinant proteins. Other tests were done in the blinded manner.

Reporting for specific materials, systems and methods

We require information from authors about some types of materials, experimental systems and methods used in many studies. Here, indicate whether each material, system or method listed is relevant to your study. If you are not sure if a list item applies to your research, read the appropriate section before selecting a response.

Materials & experimental systems

n/a	Involved in the study
☐	☒ Antibodies
☒	☐ Eukaryotic cell lines
☒	☐ Palaeontology and archaeology
☒	☐ Animals and other organisms
☒	☐ Clinical data
☒	☐ Dual use research of concern
☒	☐ Plants

Methods

n/a	Involved in the study
☒	☐ ChIP-seq
☒	☐ Flow cytometry
☒	☐ MRI-based neuroimaging

Antibodies

Antibodies used	The details of antibodies used can be found in the SI. Briefly:<br>Biotinylated Syn-211 sc-12767, santa cruz biotech, with modification*<br>AF647 Syn-211 sc-12767 AF647, santa cruz biotech Biotinylated<br>LB509 807710, BioLegend<br>AF647 LB509 sc-58480 AF647, santa cruz biotech<br>Biotinylated 6E10 803008 BioLegend<br>AF647 6E10 803021 BioLegend<br>AF488 6E10 803013 BioLegend<br>AF647 4G8 800718, BioLegend<br>Biotinylated HT7 MN1000B Invitrogen<br>AF647 HT7 51-5916-42, Invitrogen<br>AF647 Tau5 AHB0042, Invitrogen with modification*<br>Biotinylated IgG MG115, Invitrogen<br>AF647 IgG MOPC-21 ,AB_2539542, Invitrogen
Validation	These antibodies are commercially available and well-tested. References:<br>Syn-211 : Emin, Derya, et al. "Small soluble α-synuclein aggregates are the toxic species in Parkinson's disease." Nature communications 13.1 (2022): 5512.<br>LB509, 6E10: Furlepa, Martin, et al. "Single-molecule characterization of salivary protein aggregates from Parkinson's disease patients: a pilot study." Brain Communications 6.3 (2024): fcae178.<br>Tau 5 and HT7: Meredith Jr, Jere E., et al. "Characterization of novel CSF Tau and ptau biomarkers for Alzheimer's disease." PloS one 8.10 (2013): e76523.

Plants

Seed stocks	*Report on the source of all seed stocks or other plant material used. If applicable, state the seed stock centre and catalogue number. If plant specimens were collected from the field, describe the collection location, date and sampling procedures.*
Novel plant genotypes	*Describe the methods by which all novel plant genotypes were produced. This includes those generated by transgenic approaches, gene editing, chemical/radiation-based mutagenesis and hybridization. For transgenic lines, describe the transformation method, the number of independent lines analyzed and the generation upon which experiments were performed. For gene-edited lines, describe the editor used, the endogenous sequence targeted for editing, the targeting guide RNA sequence (if applicable) and how the editor was applied.*
Authentication	*Describe any authentication procedures for each seed stock used or novel genotype generated. Describe any experiments used to assess the effect of a mutation and, where applicable, how potential secondary effects (e.g. second site T-DNA insertions, mosiacism, off-target gene editing) were examined.*
