# Supplementary material for: Imaging nanoscopic co-assemblies of α-synuclein and Tau in human samples
Source: Nat Commun. 2026 Sep 18;17:10951. doi: 10.1038/s41467-026-77728-1 (PMC13680374; doi:10.1038/s41467-026-77728-1)
Supplement: Supplementary file 3 [file 41467_2026_77728_MOESM3_ESM.pdf]

nature portfolio

Peer Review File

Imaging nanoscopic co-assemblies of α-synuclein and Tau in human samples

Corresponding Author: Dr Yu Zhang

This manuscript has been previously reviewed at another journal. This document only contains information relating to versions considered at Nature Communications.

This file contains all reviewer reports in order by version, followed by all author rebuttals in order by version.

Version 0:

Reviewer comments:

Reviewer #1

(Remarks to the Author)
Most of my concerns have been addressed; however, I have a remaining point regarding the interpretation of Figure S26. The authors state that HT7 signals are higher than the IgG control regardless of HA blocker presence. However, the signal differential (αS-Tau vs. IgG) appears more robust with the HA blocker than without it ($p < 0.0001$ vs. $p = 0.0019$).
A two-way ANOVA should be used to test for a significant interaction between these variables. If the blocker indeed improves the 'true signal' (HT7 minus IgG) by reducing background noise, it would validate its necessity in the SiMPull protocol. This, in turn, suggests that the non-significant PD/control ratio shown in Figure 8A may be a more accurate reflection of the biological data.

Reviewer #2

(Remarks to the Author)
The authors have done a lot of work to address the reviewers' concerns. The testing of extra samples to confirm that initial observations were not statistical artifacts and the addition of new assays to further probe the properties of the co-assemblies (and extra controls to account for potential artifacts) considerably improve the manuscript. These changes make the work much more technically sound. The discussion is also much improved, clarifying how to understand the phenomenology of the co-assemblies studied here.

In my view, the biggest weakness of the manuscript remains the question of the significance of the co-assemblies and their properties, which the current datasets are not best suited for addressing. Although there are some differences between the co-assemblies in disease vs healthy controls, it's unclear if they have any role in disease: the presence of large numbers of co-assemblies in healthy controls might mean that they are not actually relevant to disease, or perhaps the slight differences in the properties of the diseased vs healthy co-assemblies reflect some structural evolution induced as a consequence of disease, or maybe the differences reflect populations with different neurotoxicity. The phenomenological characterizations of small co-assemblies presented here clearly have value and should be published, as there has not been much work on co-assemblies in this size regime, but without more mechanistic information that can clarify their role in disease, this manuscript might be better suited to a more topical journal.

Some minor revisions to make:
* In the introduction, I would suggest changing "the hallmark" to "hallmark", as αS aggregates are not the singular characteristic defining Parkinson's disease.
* In many of the figures in both the main text and SI, histograms are used when it would be better to show more complete distributional data, as the authors have already done in some figures (like Figs. 4-8).
* A great many of the figures in the SI do not indicate how many samples are involved in the measurement, and what the error bars represent.

Reviewer #3

(Remarks to the Author)
The authors have clearly improved the manuscript and addressed the reviewer's comments from the previous version. The manuscript can be accepted for publication after addressing these minor issues.

1. Briefly, Tau-enhanced αS aggregation could be suppressed by the addition of BSA, while αS–Tau co-assembly formation remained unchanged. TEM imaging revealed that αS and Tau form complexes with filamentous subunits, whereas BSA forms larger, amorphous complexes with αS and Tau". What is the rationale behind choosing BSA as a protein in studying the inhibitory interaction between αS and Tau?
2. Moreover, the size distribution should be calculated to come to the conclusion that indeed BSA forms larger amorphous complexes with αS and Tau.

Version 1:

Reviewer comments:

Reviewer #1

(Remarks to the Author)
This revision of Fig. S26 is confusing.
1)The interpretation provided in the rebuttal stated that the addition of the HA blocker reduced the overall signal intensity and eliminated the significance between the αS–Tau signal and the αS–IgG control. However, the figure legends of Figure S26 stated that HT7 antibody produces significantly higher signals than the IgG isotype control, irrespective of the presence of HA blocker. These conclusions are inconsistent and discrepant to each other.
2) The statistical analysis shown in Figure S26 seems confusing as well. For example, the post hoc analysis showed that the p value between αS–Tau signal and the αS–IgG control is 0.9750 in the presence of HA blocker under the two-way ANOVA analysis. However, these two groups showed a post hoc p value of <0.0001 under the one-way ANOVA analysis. I understand that different statistical methods were used, however, such huge difference is of concern. For the two-way ANOVA analysis, please provide F values and P values for the detector (HT7 bs IgG) effect, the Blocker (PBS vs. HA) effect, and detector x blocker interaction effect in addition to the post hoc analysis, and examine the post hoc analysis carefully.

Version 2:

Reviewer comments:

Reviewer #1

(Remarks to the Author)
This revision has addressed the raised issue well.

Open Access This Peer Review File is licensed under a Creative Commons Attribution 4.0 International License, which permits use, sharing, adaptation, distribution and reproduction in any medium or format, as long as you give appropriate credit to the original author(s) and the source, provide a link to the Creative Commons license, and indicate if changes were made.
In cases where reviewers are anonymous, credit should be given to 'Anonymous Referee' and the source.
The images or other third party material in this Peer Review File are included in the article's Creative Commons license, unless indicated otherwise in a credit line to the material. If material is not included in the article's Creative Commons license and your intended use is not permitted by statutory regulation or exceeds the permitted use, you will need to obtain permission directly from the copyright holder.
To view a copy of this license, visit https://creativecommons.org/licenses/by/4.0/

REVIEWER COMMENTS

Reviewer #1 (Remarks to the Author):

Most of my concerns have been addressed; however, I have a remaining point regarding the interpretation of Figure S26. The authors state that HT7 signals are higher than the IgG control regardless of HA blocker presence. However, the signal differential (αS-Tau vs. IgG) appears more robust with the HA blocker than without it ($p < 0.0001$ vs. $p = 0.0019$).
A two-way ANOVA should be used to test for a significant interaction between these variables. If the blocker indeed improves the 'true signal' (HT7 minus IgG) by reducing background noise, it would validate its necessity in the SiMPull protocol. This, in turn, suggests that the non-significant PD/control ratio shown in Figure 8A may be a more accurate reflection of the biological data.

We thank the reviewer for this helpful suggestion. We agree that the comparison in Fig. S26 is better evaluated by testing the interaction between antibody signal and blocking condition (two-way ANOVA), rather than by relying only on separate one-way comparisons. We have therefore performed a two-way ANOVA to examine whether the HA blocker significantly improves the specific αS–Tau signal, defined by the difference between the HT7 signal and the corresponding IgG control.

This additional analysis led us to revise our interpretation. Contrary to the apparent trend suggested by the separate one-way comparisons, the two-way ANOVA did not support the conclusion that the HA blocker enhances the true αS–Tau signal. Instead, when PBS was used as the diluent, the αS–Tau signal remained significantly higher than the IgG control, supporting specific detection. However, the addition of the HA blocker reduced the overall signal intensity and eliminated this significance. This suggests that the HA blocker does not simply reduce background noise, but also suppresses the detectable αS–Tau signal, most likely because of excessive blocking under these conditions.

This interpretation is further supported by the signal-reduction effect observed in Fig. S.27. Therefore, we do not consider the HA blocker to improve the accuracy of αS–Tau detection in this specific SiMPull assay. Rather, the PBS condition better preserves the specific signal while maintaining an acceptable IgG background. We have revised the manuscript accordingly (line 579-600) to clarify that the non-significant PD/control ratio in Fig. 8A is not a consequence of HA-blocker-mediated signal improvement but reflects the assay condition that best preserves detectable αS–Tau co-assemblies.

Reviewer #2 (Remarks to the Author):

The authors have done a lot of work to address the reviewers' concerns. The testing of extra samples to confirm that initial observations were not statistical artifacts and the addition of new assays to further probe the properties of the co-assemblies (and extra controls to account for potential artifacts) considerably improve the manuscript. These changes make the work much more technically sound. The discussion is also much improved, clarifying how to understand the phenomenology of the co-assemblies studied here.

In my view, the biggest weakness of the manuscript remains the question of the significance of the co-assemblies and their properties, which the current datasets are not best suited for addressing. Although there are some differences between the co-assemblies in disease vs healthy controls, it's unclear if they have any role in disease: the presence of large numbers of co-assemblies in healthy controls might mean that they are not actually relevant to disease, or perhaps the slight differences in the properties of the diseased vs healthy co-assemblies reflect some structural evolution induced as a consequence of disease, or maybe the differences reflect populations with different neurotoxicity. The phenomenological characterizations of small co-assemblies presented here clearly have value and should be published, as there has not been much work on co-assemblies in this size regime, but without more mechanistic information that can clarify their role in disease, this manuscript might be better suited to a more topical journal.

We thank the reviewer for this thoughtful assessment. Our current datasets should not be interpreted as assigning a single disease-driving mechanism to αS–Tau co-assemblies, and we have substantially revised the Discussion to make this point explicit. In particular, we avoid presenting these co-assemblies as uniformly pathogenic. Instead, we emphasise that their frequent detection in both disease and control samples argues against a simple disease-specific interpretation and supports what we consider a central conclusion of the study: nanoscopic co-assemblies are heterogeneous entities whose biological consequences are likely context-dependent. (e.g. extracellular vs intracellular)

We also wish to clarify that obtaining definitive "mechanistic insight" for these species is challenging at the current stage of the field. The co-assemblies detected here are nanoscopic, low-abundance, structurally heterogeneous, and present in complex human-derived samples. They cannot currently be isolated as a single molecular species, assigned to a defined cellular origin, or directly linked to toxicity, propagation, or protection without first resolving their subpopulations. Therefore, assigning one mechanistic role to all detected αS–Tau co-assemblies would be premature and

potentially misleading. In this context, the absence of a single mechanistic assignment is not simply a limitation of the study but reflects the biological and technical complexity of the assemblies themselves.

We have therefore reframed the significance of the work more clearly. Rather than claiming that αS–Tau co-assemblies have one uniform role in disease, we now also present the study as establishing a technically controlled framework for detecting and comparing previously inaccessible human-derived nanoscopic co-assemblies across brain fractions and biofluids. This framework reveals co-assembly subpopulations with distinct epitope exposure, protease resistance, sample-fraction dependence, and disease-associated differences. These observations provide an essential foundation for future mechanistic studies, because such studies first require reliable detection, quantification, and comparison of the relevant species in human samples.

We have further revised the Discussion to state more explicitly that future work will need to isolate or enrich specific co-assembly subfractions and determine whether individual populations are toxic, protective, compensatory, or secondary products of disease-associated structural remodelling. The whole Discussion has therefore been re-arranged following the reviewer's advice, with a stronger emphasis on heterogeneity, context dependence, and the technical challenge of moving from phenomenological characterisation to mechanistic assignment in human-derived nanoscopic protein assemblies.

Some minor revisions to make:
* In the introduction, I would suggest changing “the hallmark” to “hallmark”, as αS aggregates are not the singular characteristic defining Parkinson's disease.
* In many of the figures in both the main text and SI, histograms are used when it would be better to show more complete distributional data, as the authors have already done in some figures (like Figs. 4-8).
* A great many of the figures in the SI do not indicate how many samples are involved in the measurement, and what the error bars represent.

We have revised all the technical issues.

Reviewer #3 (Remarks to the Author):

The authors have clearly improved the manuscript and addressed the reviewer's comments from the previous version. The manuscript can be accepted for publication after addressing these minor issues.

1. Briefly, Tau-enhanced αS aggregation could be suppressed by the addition of BSA, while αS–Tau co-assembly formation remained unchanged. TEM imaging revealed that αS and Tau form complexes with filamentous subunits, whereas BSA

forms larger, amorphous complexes with αS and Tau". What is the rationale behind choosing BSA as a protein in studying the inhibitory interaction between αS and Tau?

We thank the reviewer for raising this important point. BSA was selected not as a disease-specific interaction partner, but as a model albumin/background protein to test whether Tau-enhanced αS aggregation is maintained in a protein-rich environment. Albumin is highly relevant in this context because human serum albumin is the most abundant protein in serum and CSF and has been reported to exhibit chaperone-like activity toward aggregation-prone proteins. Previous work has also shown that albumin can directly interact with αS and modulate its aggregation in a pH- and ionic-strength-dependent manner, with HSA at serum concentrations significantly slowing αS aggregation. (DOI: 10.1021/acs.jpcb.9b02381) In addition, BSA shares substantial sequence similarity with HSA and has previously been used to study albumin-mediated effects on αS fibrillization under near-physiological buffer conditions. (doi: 10.1002/jmr.699.)

Therefore, our purpose in adding BSA was to introduce an abundant, soluble, non-amyloidogenic albumin-like protein that could mimic one aspect of the crowded protein environment encountered in biological fluids, rather than to imply that BSA itself is a specific pathological regulator of αS or Tau. This experiment allowed us to distinguish two related but separable processes: Tau-mediated acceleration of αS fibrillization and αS–Tau co-assembly formation. We found that BSA suppressed Tau-enhanced αS aggregation, while αS–Tau co-assembly formation remained largely unchanged. This suggests that Tau-driven acceleration of αS fibrillization is sensitive to the surrounding protein environment, whereas αS–Tau association can still occur under these conditions. We have revised the manuscript accordingly (line 619-628)

TEM imaging further supported this interpretation. αS and Tau formed complexes containing filamentous subunits, whereas the addition of BSA shifted the morphology toward larger amorphous complexes containing αS and Tau. These findings suggest that BSA alters the assembly pathway or higher-order organization of αS and Tau, rather than simply preventing their interaction. We have revised the manuscript to clarify this rationale and to avoid implying that BSA was chosen as a disease-specific inhibitor.

2. Moreover, the size distribution should be calculated to come to the conclusion that indeed BSA forms larger amorphous complexes with αS and Tau.

We replaced intensity quantification in the figure S29 to size quantification following the advice.

Reviewer #1 (Remarks to the Author):

This revision of Fig. S26 is confusing.
1)The interpretation provided in the rebuttal stated that the addition of the HA blocker reduced the overall signal intensity and eliminated the significance between the αS–Tau signal and the αS–IgG control. However, the figure legends of Figure S26 stated that HT7 antibody produces significantly higher signals than the IgG isotype control, irrespective of the presence of HA blocker. These conclusions are inconsistent and discrepant to each other.
2) The statistical analysis shown in Figure S26 seems confusing as well. For example, the post hoc analysis showed that the p value between αS–Tau signal and the αS–IgG control is 0.9750 in the presence of HA blocker under the two-way ANOVA analysis. However, these two groups showed a post hoc p value of <0.0001 under the one-way ANOVA analysis. I understand that different statistical methods were used, however, such huge difference is of concern. For the two-way ANOVA analysis, please provide F values and P values for the detector (HT7 bs IgG) effect, the Blocker (PBS vs. HA) effect, and detector x blocker interaction effect in addition to the post hoc analysis, and examine the post hoc analysis carefully.

We thank the reviewer for pointing this inconsistency. We have corrected the legend in Fig. S26 and re-examined the statistics.

Specifically, the difference between one-way ANOVA and two-way ANOVA (p value obtained from αS–Tau vs. IgG with HA blocker: 0.9750 vs. <0.0001) is due to inappropriate statistical assumptions. The distribution of our dataset is highly skewed; therefore a non-parametric one-way ANOVA was used in the first version (p<<0.0001). However, the two-way ANOVA in our analysis software (Graph pad) does not offer non-parametric options, and the p=0.9750 is obtained based on Gaussian assumptions. When we did the log transform for the raw data, and re-apply the two-way ANOVA with Gaussian assumption, results are getting more like the one-way ANOVA. This confirmed the origin of the inconsistency pointed out by the reviewer. The details of 3 different tests are shown below:

One way ANOVA (Non-parametric):

Dunn's multiple comparisons test	Mean rank diff.	Significant?	Summary	Adjusted P Value
αS-Tau (HA-) vs. αS-Tau (HA+)	23.09	No	ns	0.0617
αS-Tau (HA-) vs. IgG (HA-)	33.30	Yes	**	0.0020
αS-Tau (HA-) vs. IgG (HA+)	64.83	Yes	****	<0.0001
αS-Tau (HA+) vs. IgG (HA-)	10.21	No	ns	>0.9999
αS-Tau (HA+) vs. IgG (HA+)	41.74	Yes	****	<0.0001
IgG (HA-) vs. IgG (HA+)	31.53	Yes	**	0.0060

Two-way ANOVA (Gaussian):

Source of Variation	% of total variation	P value	P value summary	Significant?
Interaction	1.671	0.1235	ns	No
Row Factor (HA)	8.444	0.0007	***	Yes
Column Factor (Detector)	3.196	0.0340	*	Yes

ANOVA table	SS (Type III)	DF	MS	F (DFn, DFd)	P value
Interaction	170337	1	170337	F (1, 124) = 2.404	P=0.1235
Row Factor (HA)	860790	1	860790	F (1, 124) = 12.15	P=0.0007
Column Factor (Detector)	325750	1	325750	F (1, 124) = 4.598	P=0.0340
Residual	8784803	124	70845		
Total	10193776	127			

Tukey's multiple comparisons test	Predicted (LS) Mean diff.	95.00% CI of diff.	Below threshold?	Summary	Adjusted P Value
IgG (HA-) vs αS-Tau (HA-)	-174.2	-347.8 to -0.5654	Yes	*	0.0489
αS-Tau (HA+):vs αS-Tau (HA-)	-237.4	-405.5 to -69.32	Yes	**	0.0020
IgG (HA+) vs αS-Tau (HA-)	-265.4	-439.1 to -91.80	Yes	***	0.0007
αS-Tau (HA+) vs IgG (HA-)	-63.24	-236.9 to 110.4	No	ns	0.7787
IgG (HA+) vs IgG (HA-)	-91.23	-270.2 to 87.74	No	ns	0.5473
IgG (HA+) vs.αS-Tau (HA+)	-27.99	-201.6 to 145.6	No	ns	0.9750

Two-way ANOVA (with Log transformation of raw data):

Source of Variation	% of total variation	P value	P value summary	Significant?
Interaction	0.1607	0.5807	ns	No
Row Factor (HA)	15.05	<0.0001	****	Yes
Column Factor (Detector)	19.95	<0.0001	****	Yes

ANOVA table	SS (Type III)	DF	MS	F (DFn, DFd)	P value
Interaction	0.1257	1	0.1257	F (1, 124) = 0.3067	P=0.5807
Row Factor (HA)	11.77	1	11.77	F (1, 124) = 28.72	P<0.0001
Column Factor (Detector)	15.60	1	15.60	F (1, 124) = 38.07	P<0.0001
Residual	50.82	124	0.4098		
Total	78.22	127			

Tukey's multiple comparisons test	Predicted (LS) Mean diff.	95.00% CI of diff.	Below threshold?	Summary	Adjusted P Value
IgG (HA-) vs αS-Tau (HA-)	0.6369	0.2193 to 1.054	Yes	***	0.0007
αS-Tau (HA+):vs αS-Tau (HA-)	0.5449	0.1405 to 0.9492	Yes	**	0.0035
IgG (HA+) vs αS-Tau (HA-)	1.307	0.8897 to 1.725	Yes	****	<0.0001

αS-Tau (HA+) vs IgG (HA-)	-0.09202	-0.5096 to 0.3256	No	ns	0.9397
IgG (HA+) vs IgG (HA-)	0.6705	0.2400 to 1.101	Yes	***	0.0005
IgG (HA+) vs.αS-Tau (HA+)	0.7625	0.3449 to 1.180	Yes	****	<0.0001

However, the Log-transformed raw data is still not Gaussian (e.g. the IgG counting for HA- has two populations), an Aligned Rank Transform (ART) ANOVA was used for statistical tests. We also added additional data to make sure αS-Tau and IgG sets have same n number. In the previous version, 4 samples (2PD, 2HC) were not examined for IgG non-specific binding. With the complete dataset and new statistic model, we have:

Group	n	Mean	Median
αS-Tau(HA-)	34	278.42	61.26
IgG (HA-)	34	92.60	7.96
αS-Tau(HA+)	34	40.99	23.92
IgG (HA+)	34	11.75	2.96

Effect	df1	df2	F value	p value
Detector, HT7(αS-Tau) vs IgG	1	132	3.9853	**0.04796**
Blocker, HA- vs HA+	1	132	9.0803	**0.00310**
Detector × Blocker	1	132	1.7796	0.18449

Comparison	F value	Unadjusted p	Tukey-adjusted p
αS-Tau (HA-) vs IgG, (HA-)	28.2058	4.50e-7	**2.67e-6**
αS-Tau (HA+) vs IgG, (HA+)	40.4889	3.00e-9	**1.80e-8**
αS-Tau (HA-) vs αS-Tau (HA+)	10.0904	0.00186	**0.00991**
IgG (HA-) vs IgG (HA+)	17.8821	4.36e-5	**0.000253**
αS-Tau (HA-) vs IgG (HA+)	91.0046	1.00e-16	**1.28e-14**
IgG (HA-) vs αS-Tau (HA+)	4.5555	0.03466	0.14767

From this rank-based ART analysis, we can see that αS-Tau signals were significantly higher than IgG controls overall, and HA blocker significantly reduced the overall serum SiMPull readout. Multiple comparisons showed that αS-Tau remained significantly higher than IgG both without and with HA blocker. However, because the Detector × Blocker interaction was not significant, we do not conclude that HA blocker selectively enhances the true αS–Tau signal relative to IgG background. Instead, we interpret HA blocking as increasing assay stringency by reducing serum-associated background while also attenuating part of the positive signal.

From a biology aspect, we agree with the reviewer that the PBST-only serum signal should not be interpreted as unequivocal evidence of true αS–Tau assembly abundance. The relatively high IgG-control signal in a subset of PBST-only serum samples indicates that serum-associated nonspecific binding or matrix effects can also contribute to the readout. However, because HT7 signals remained significantly higher than IgG controls under both PBST-only and HA-blocked conditions, we do not interpret the PBST-only signal as purely false positive either. Instead, we now interpret the PBST-only signal as a mixture of target-dependent αS–Tau signal and serum-associated background/matrix effects.

We have therefore revised the manuscript wording to avoid overstatement. (line 565-627) We now describe the result as "detectable" or "apparent" αS–Tau SiMPull signal under defined assay conditions, rather than as a direct measurement of total αS–Tau assembly abundance in serum. We also clarify that HA blocking increases assay stringency by reducing serum-associated background, but it also attenuates part of the positive signal and therefore introduces a sensitivity trade-off. (supported by Fig. S27) This interpretation is consistent with the loss of disease-group separation after HA blocking, suggesting that the PBST-only disease-associated difference may have been influenced, at least in part, by serum matrix effects or nonspecific background.
